# Genome mining of novel rubiginones from *Streptomyces* sp. CB02414 and characterization of the post-PKS modification steps in rubiginone biosynthesis

**DOI:** 10.1186/s12934-021-01681-5

**Published:** 2021-10-02

**Authors:** Jingyan Zhang, Ying Sun, Yeji Wang, Xin Chen, Lu Xue, Jingjing Zhang, Xiangcheng Zhu, Yanwen Duan, Xiaohui Yan

**Affiliations:** 1 Xiangya International Academy of Translational Medicine, Central South University, Tongzipo Road, #172, Yuelu District, Changsha, 410013 Hunan China; 2 State Key Laboratory of Component-Based Chinese Medicine, Tianjin University of Traditional Chinese Medicine, Tianjin, China; 3 Hunan Engineering Research Center of Combinatorial Biosynthesis and Natural Product Drug Discovery, Changsha, Hunan China; 4 National Engineering Research Center of Combinatorial Biosynthesis for Drug Discovery, Changsha, Hunan China

**Keywords:** Genome mining, Rubiginones, Cytochrome P450 hydroxylase, Biosynthesis

## Abstract

**Background:**

Rubiginones belong to the angucycline family of aromatic polyketides, and they have been shown to potentiate the vincristine (VCR)-induced cytotoxicity against VCR-resistant cancer cell lines. However, the biosynthetic gene clusters (BGCs) and biosynthetic pathways for rubiginones have not been reported yet.

**Results:**

In this study, based on bioinformatics analysis of the genome of *Streptomyces* sp. CB02414, we predicted the functions of the two type II polyketide synthases (PKSs) BGCs. The *rub* gene cluster was predicted to encode metabolites of the angucycline family. Scale-up fermentation of the CB02414 wild-type strain led to the discovery of eight rubiginones, including five new ones (rubiginones J, K, L, M, and N). Rubiginone J was proposed to be the final product of the *rub* gene cluster, which features extensive oxidation on the A-ring of the angucycline skeleton. Based on the production profiles of the CB02414 wild-type and the mutant strains, we proposed a biosynthetic pathway for the rubiginones in CB02414.

**Conclusions:**

A genome mining strategy enabled the efficient discovery of new rubiginones from *Streptomyces* sp. CB02414. Based on the isolated biosynthetic intermediates, a plausible biosynthetic pathway for the rubiginones was proposed. Our research lays the foundation for further studies on the mechanism of the cytochrome P450-catalyzed oxidation of angucyclines and for the generation of novel angucyclines using combinatorial biosynthesis strategies.

**Supplementary Information:**

The online version contains supplementary material available at 10.1186/s12934-021-01681-5.

## Background

Angucyclines are aromatic polyketides with an angular tetracyclic benz[*a*]anthracene skeleton [1]. By virtue of their structural and functional diversity, angucyclines have greatly held the attention of chemists and biologists. Since the isolation of tetrangomycin from *Streptomyces rimosus* in 1965 [2], the number of angucyclines increased steadily, with more than 300 compounds discovered to date. The benz[*a*]anthracene scaffold of angucycline is formed by folding a decaketide chain, which is biosynthesized by the type II polyketide synthases (PKSs), to generate the UWM6 intermediate [3]. This common intermediate is then decorated by various post-PKS modifications, such as oxidations [4, 5], ring rearrangement/contraction [6], and glycosylations [7, 8], to form numerous angucyclines. The angucyclines have various biological activities, such as antibacterial, anticancer, antiviral, enzyme inhibition, and platelet-aggregation inhibition [9].

Rubiginones, a subgroup of the angucycline family, were first isolated from *Streptomyces griseorubiginosus* No. Q144-2 in a screening program for potentiators of vincristine (VCR) cytotoxicity [10]. Six components, i.e., rubiginones A_1_, A_2_, B_1_, B_2_, C_1_, and C_2_, were isolated from this strain. Rubiginone B_1_ was reported to potentiate the cytotoxicity of VCR by inhibiting VCR efflux [11]. Rubiginone A_2_ and B_2_ were also discovered from *Streptomyces* sp. SNA-8073 [12] and *Saccharopolyspora* sp. BCC 21906, respectively [13]. The Zeeck group isolated four new rubiginones, named rubiginone D_2_, H, I and 4-*O*-acetyl-rubiginone D_2_, from *Streptomyces* sp. Gö N1/5 [14]. Hayakawa et al. isolated four new rubiginone analogues, including hatomarubigins A, B, C, and D, from *Streptomyces* sp. 2238-SVT4. Rubiginone B_2_ was also isolated from *Streptomyces* sp. 2238-SVT4 and it was proposed to be a biosynthetic intermediate for the hatomarubigins [15].

Although the rubiginones have been identified for three decades and have been discovered from multiple actinomycetes, their biosynthetic gene clusters (BGCs) and biosynthetic pathways have not been characterized yet. In 2010, Kawasaki et al*.* characterized the *hrb* gene cluster for hatomarubigin biosynthesis in *Streptomyces* sp. 2238-SVT4, which consists of 30 open reading frames (ORFs) [16]. Heterologous expression of a part of the *hrb* gene cluster in *Streptomyces lividans* TK23 resulted in the production of hatomarubigins A, B, C, and rubiginone B_2_ [16]. In this study, we utilized the forward (from genes to metabolites) genome mining approach to characterize the two type II PKS BGCs in *Streptomyces* sp*.* CB02414 and correlated the *rub* gene cluster with the production of eight rubiginones, including five novel ones (rubiginones J, K, L, M, and N), from the CB02414 wild-type and gene-deletion mutants. Of the eight rubiginones, rubiginone J was identified as the final product of the *rub* gene cluster, and based on the structures of the isolated rubiginones, a biosynthetic pathway for the rubiginones was proposed.

## Results

### Bioinformatics analysis of the *Streptomyces* sp. CB02414 genome

*Streptomyces* sp. CB02414 was isolated from a soil sample collected on the beach of Dubai, United Arab Emirates. It was initially screened as a potential enediyne producer [17] and the presence of an enediyne BGC in the CB02414 genome (Accession number LIPF00000000.1) was confirmed by the antiSMASH analysis [18]. According to the antiSMASH result, CB02414 contains 27 BGCs, including three BGCs encoding nonribosomal peptides, six BGCs for terpenoids, three BGCs for siderophores and six BGCs for polyketides (Additional file 1: Table S1). Among the six polyketide BGCs, the two type II PKS gene clusters (cluster 5 and cluster 20) attracted our attention and were subjected to further bioinformatic analysis.

### Bioinformatics analysis of cluster 5 reveals a possible spore pigment BGC

**Fig. 1:**
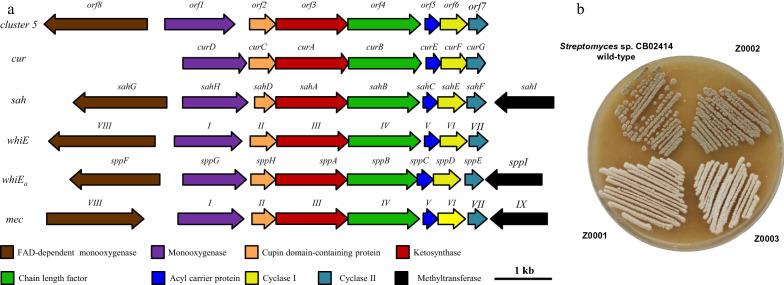
Comparison of cluster 5 in *Streptomyces* sp. CB02414 with other BGCs for spore pigment biosynthesis (**A**), and spores of CB02414 (wild-type) and mutants Z0001–Z0003 on the GYM agar plate (**B**)

Analysis of cluster 5 revealed eight ORFs that encode proteins with high sequence similarities (identity > 66%) to proteins from spore pigment BGCs, such as the *whiE* gene cluster from *Streptomyces coelicolor* A3(2) [19], the *whiEa* gene cluster from *Streptomyces avermitilis* [20], the *cur* gene cluster from *Streptomyces curacoi* [21], the *sah* gene cluster from *Streptomyces sahachiroi* ATCC 33158 [22], the *mec* gene cluster from *Streptomyces bottropensis* [23], the *whiESa* gene cluster from *Streptomyces aureofaciens* CCM3239 [24], and the *pksA* gene cluster from *Streptomyces collinus* DSM2012 (Accession number AF293354.1, unpublished data) (Fig. 1A). In addition to the high sequence similarity, cluster 5 displays identical gene organization to the *whiE* gene cluster. These results suggested that cluster 5 might be responsible for the biosynthesis of spore pigment in CB02414.

### Cluster 20 displays high similarity with the *hrb* gene cluster

Annotation of cluster 20 (the *rub* cluster) revealed a 44-kbp DNA fragment consisting of 38 putative ORFs, and most of the encoded proteins showed high similarity with proteins from the *hrb* gene cluster, which is responsible for the biosynthesis of the angucyclines hatomarubigins A, B, C, and D in *Streptomyces* sp. 2238-SVT4 [16] (Table 1). Therefore, we proposed that the *rub* gene cluster is responsible for the biosynthesis of natural products of the angucycline family.

**Table 1:** Proposed functions of proteins of the *rub* BGC in *Streptomyces* sp. CB02414

Protein	Size (aa)	Proposed function	Homologs	
			Protein/organism	Accession no. (similarity/identity %)
Rub(-1)	258	AfsR/SARP family regulator	MoaR2/*Streptomyces* sp. S4.7	QHY99565.1 (54/68)
RubA1	237	Regulator	HrbR3	BAJ07866.1 (54/66)
RubB1	235	Oxidoreductase	HrbS	BAJ07858.1 (48/61)
RubC	279	Type II toxin-antitoxin chaperone	HigA/*Pseudomonas fluorescens* F113	AEV65067.1 (51/65)
RubD1	489	Oxygenase	HrbR	BAJ07857.1 (70/78)
RubE	108	Cyclase	HrbB	BAJ07841.1 (70/78)
RubF1	424	Ketosynthase (KS_α_)	HrbC	BAJ07842.1 (80/89)
RubF2	406	Chain length factor (KS_β_)	HrbE	BAJ07844.1 (69/80)
RubF3	85	Acyl carrier protein	HrbM	BAJ07852.1 (59/67)
RubG	261	Ketoreductase	HrbO	BAJ07854.1 (80/87)
RubH	361	Aromatase	HrbP	BAJ07855.1 (67/79)
RubI1	678	Hydroxylase	HrbG	BAJ07846.1 (68/78)
RubB2	256	Oxidoreductase	HrbS	BAJ07858.1 (71/81)
RubJ	522	Decarboxylase	JadN/*Streptomyces venezuelae*	AAK01934.1 (86/92)
RubK	71	Acyl-CoA carboxylase subunit	C7987_1162/*Streptomyces venezuelae*	PWW46500.1 (69/77)
RubD2	322	Oxygenase	HrbH	BAJ07847.1 (77/85)
RubL	414	Transporter	HrbT	BAJ07859.1 (59/70)
RubI2	206	Hydroxylase	HrbW	BAJ07862.1 (52/68)
RubD3	221	Antibiotic biosynthesis monooxygenase	ADL21_13530/*Streptomyces albus*	KWT61349.1 (36/49)
RubM1	228	Methyltransferase	HrbF	BAJ07845.1 (44/58)
RubA2	201	TetR family regulator	BetI/*Streptomyces* sp. Go-475	AXE89736.1 (76/84)
RubB3	275	Oxidoreductase	HrbS	BAJ07858.1 (24/41)
RubN1	404	Cytochrome P450	NcmG/*Saccharothrix syringae*	ARS01480.1 (54/66)
RubM2	288	Methyltransferase	DWB77_01891	AYG79773.1 (48/58)
RubO	260	Alpha/beta hydrolase	DWB77_01892	AYG79774.1 (47/64)
RubA3	212	Lys family regulator	DWB77_01893	AYG79775.1 (58/69)
RubP1	254	Hypothetical protein	DWB77_01894	AYG79776.1 (59/71)
RubP2	373	Hypothetical protein	DWB77_01895	AYG79777.1 (70/78)
RubB4	488	Oxidoreductase	DWB77_01896	AYG79778.1 (57/65)
RubM3	291	Methyltransferase	DWB77_01897	AYG79779.1 (80/88)
RubP3	567	GH3 auxin-responsive promoter	DWB77_01898	AYG79780.1 (45/59)
RubP4	209	Hypothetical protein	DWB77_01899	AYG79781.1 (66/77)
RubQ	431	Acyltransferase	PauY24/*Streptomyces* sp. YN86	AIE54244.1 (44/57)
RubR	71	Ferredoxin	HbmFdx/*Streptomyces hygroscopicus*	AAY28236.1 (50/62)
RubN2	403	Cytochrome P450	SceD/*Streptomyces* sp. SD85	ANH11399.1 (47/63)
RubA4	225	TetR regulatory protein	AbmC/*Streptomyces koyangensis*	AVI57423.1 (50/62)
RubL2	493	MFS transporter	AbmD/*Streptomyces koyangensis*	AVI57425.1 (46/65)
RubM4	345	*O*-Methyltransferase	HrbU	BAJ07860.1 (60/74)
RubS	585	Acetyl-/propionyl-CoA carboxylase subunit alpha	JadJ/*Streptomyces venezuelae*	AAD37851.1 (86/91)
Rub(+ 1)	407	Methionine adenosyltransferase	SLA_0973/*Streptomyces laurentii*	BAU81924.1 (92/95)

^a^The *hrb* homologous proteins are from *Streptomyces* sp. 2238-SVT4

^b^The DWB77_01891 to DWB77_01899 proteins are from *Streptomyces hundungensis*

**Fig. 2:**
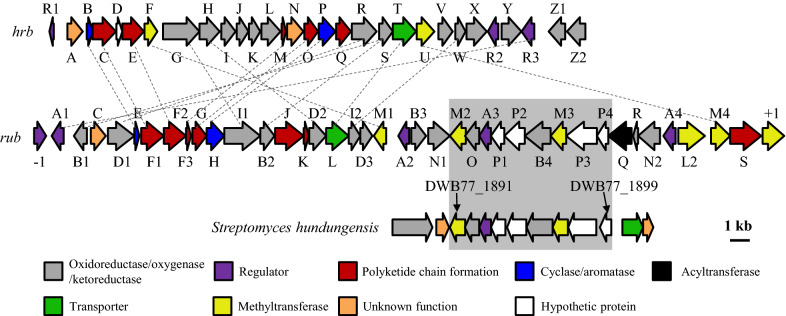
The organization of the *rub* gene cluster in *Streptomyces* sp. CB02414

The minimal PKS gene cassette (*rubF1*, *rubF2*, and *rubF3*) is located near the left boundary of the *rub* gene cluster. Interestingly, there is an 8.8-kbp DNA region consisting of nine ORFs (*rubM2* to *rubP4*) with identical organization to the fragment encoding proteins DWB77_01891 to DWB77_01899 from *Streptomyces hundungensis* (Fig. 2). The protein similarities between the nine ORFs from CB02414 and their respective homologues from *S. hundungensis* range from 45 to 80%, and the DNA sequence identity between these two fragments is 78%. AntiSMASH analysis of the *S. hundungensis* genome (Accession number CP032698.1) showed that the fragment containing proteins DWB77_1891 to DWB77_1899 is not located in a recognizable BGC. Moreover, the nine ORFs in the *rub* gene cluster do not exhibit similarities to proteins from the other known BGCs for angucycline or aromatic polyketide. Therefore, the 8.8-kbp DNA region in the *rub* gene cluster might arise from an insertion event caused by horizontal gene transfer or by transposition.

### Inactivation of the minimal PKSs in the two type II PKS gene clusters leading to different phenotypes

To investigate the functions of the two type II PKS BGCs in CB02414, we inactivated *orf3*/*orf4* (encoding ketosynthase and chain length factor, respectively) in cluster 5, to generate the mutant strain Z0001, and *rubF1*/*rubF2* to generate the mutant strain Z0002, respectively. In order to avoid the possible cross complementation between the two minimal PKS gene cassettes of cluster 5 and the *rub* cluster, we also constructed a double-deletion mutant Z0003, in which the genes encoding the two ketosynthases and the two chain length factors in cluster 5 and the *rub* cluster were inactivated together (Additional file 1: Fig. S1).

**Fig. 3:**
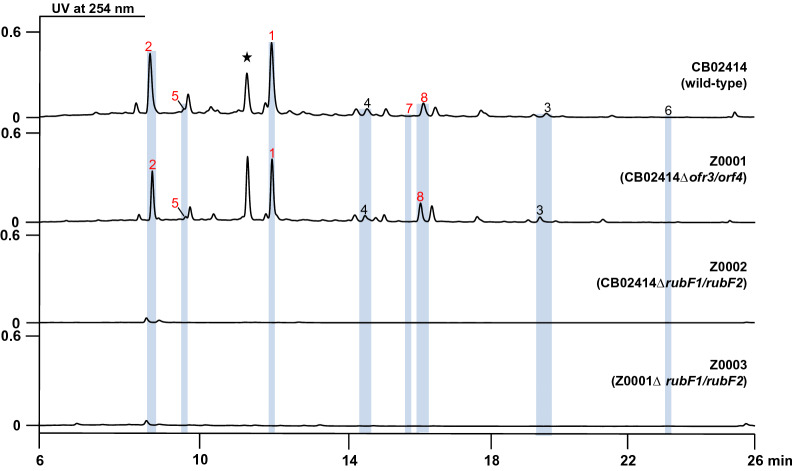
HPLC analysis of the rubiginone production in *Streptomyces* sp. CB02414 (wild-type) and the Z0001–Z0003 mutants

The CB02414 wild-type and the Z0002 mutant strains produced grey pigmentation on the GYM agar plate, while the color of the spores of Z0001 and Z0003 was pale yellow on the same plate (Fig. 1B). Comparison of the HPLC profiles of the CB02414 wild-type and the Z0001 mutant did not reveal noticeable difference, while most of the peaks at 254 nm (compounds **1**–**8**) disappeared in Z0002 and Z0003 (Fig. 3). These results clearly demonstrated that cluster 5 is involved in the formation of spore pigment in CB02414, and the *rub* gene cluster is responsible for the biosynthesis of compounds **1**–**8**.

### Large-scale fermentation and structural elucidation of the rubiginones from the CB02414 wild-type strain

**Fig. 4:**
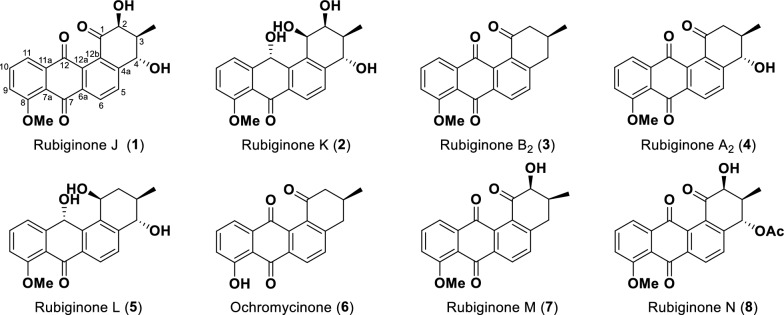
Rubiginones isolated from *Streptomyces* sp. CB02414

**Fig. 5:**
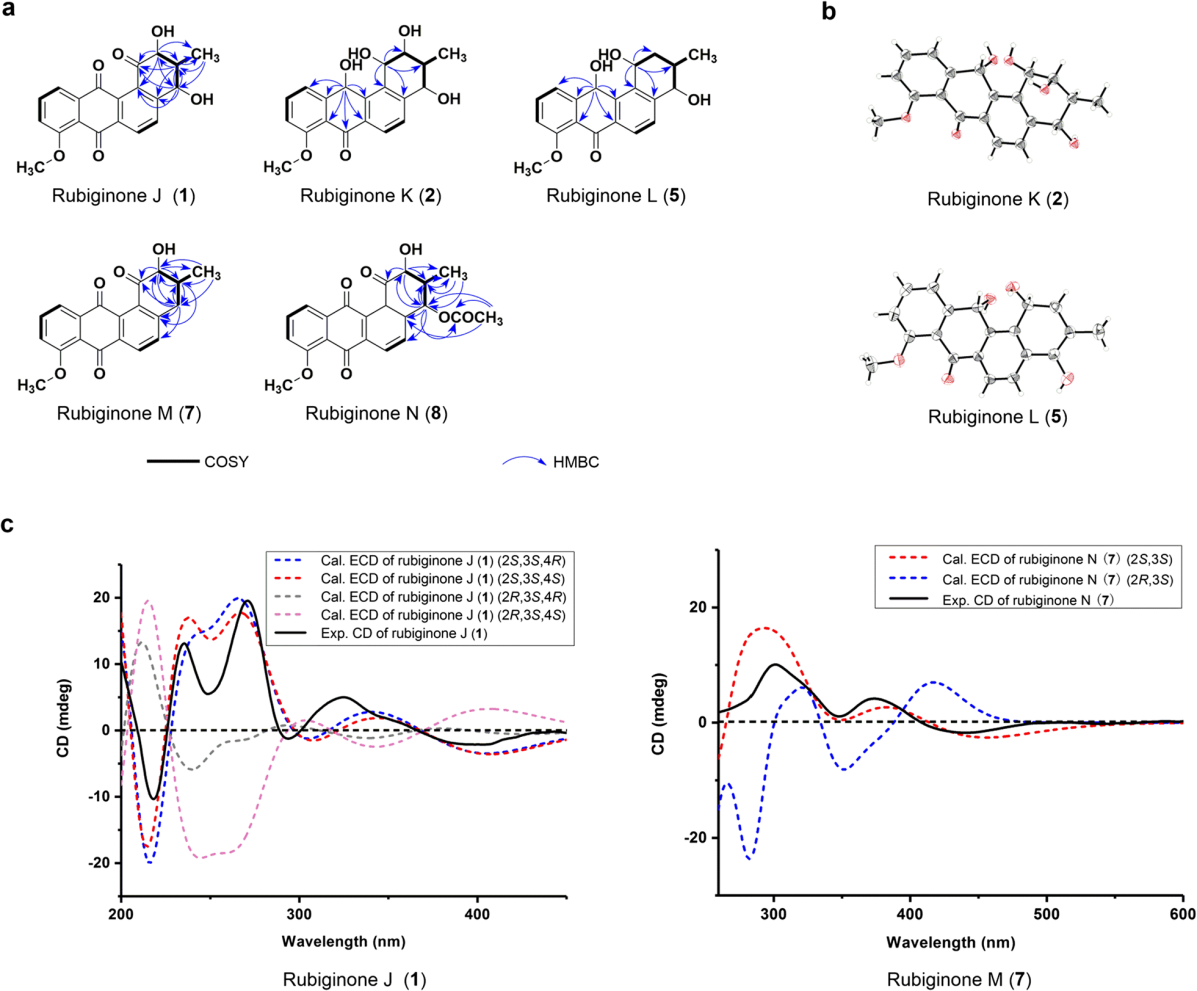
Structure elucidation of compounds **1**, **2**, **5**, **7** and **8**. **a** COSY and HMBC correlations of compounds **1**, **2**, **5**, **7** and **8**. **b** The X-ray Crystallographic study of compounds **2** and **5** (30% probability displacement ellipsoids). **c** The CD spectra of compounds **1** and **7**

The CB02414 wild-type strain was cultivated in three different media (medium B, C, and F), and the abundances of compounds **1**–**8** in these media were analyzed. Medium C was selected for large-scale fermentation because of the highest titers for compounds **1**–**8**. (Additional file 1: Fig. S2). Eight rubiginone analogues were isolated and characterized from an 8-L fermentation of the CB02414 wild-type strain, including five new compounds (compounds **1**, **2**, **5**, **7**, and **8**) whose structures were established by extensive spectral analysis (Fig. 4). The five new compounds were named rubiginones J (**1**), K (**2**), L (**5**), M (**7**), and N (**8**), respectively (Fig. 5). Rubiginones K and L are different from other known rubiginones, because their C-1 and C-12 positions have hydroxyl groups instead of carbonyl groups found in other rubiginones. The identities of rubiginone B_2_ (**3**) [10], rubiginone A_2_ (**4**) [10], and ochromycinone (**6**) [25] were determined by comparing their individual ^1^H and ^13^C NMR data with the NMR data from literature (Additional file 1: Figs. S3–S8). The C-3 methyl group in compounds **3**, **4**, and **6** has a β-configuration. Because this configuration arises from the early cyclization step of the angucycline skeleton biosynthesis, it is reasonable to speculate that the C-3 methyl group of all the rubiginones from CB02414 adopts a β-configuration. The peak marked with asterisk (Fig. 3) has different UV spectra from compound **1**, but it was converted quickly to compound **1** (< 1 h) during the isolation and purification steps, and we were not able to determine its structure (Additional file 1: Fig. S9).

Rubiginone J (**1**) was obtained as light-yellow needles. Its molecular formula C_20_H_16_O_6_ was established upon analysis of the HR-ESI–MS peak at *m/z* 353.1022 [M+H]^+^ (calculated for the [M+H]^+^ ion at 353.1020) (Additional file 1: Fig. S10). Rubiginone J has the same molecular formula as rubiginone D_2_, but the NMR data and NOESY correlation indicted that the C-4 hydroxyl group of the former compound has an α-configuration, while that of the latter compound has a β-configuration. The relative structure of compound **1** was determined by the ^1^H–^1^H COSY, HSQC and HMBC data (Additional file 1: Figs. S11–S17; Table S2). The absolute configuration of compound **1** was further supported by circular dichroism (CD) and electronic circular dichroism (ECD) analysis (Fig. 5c; Additional file 1: Figs. S18–S21).

Rubiginone K (**2**) was obtained as brown oil. The molecular formula C_20_H_20_O_6_ was established upon analysis of the HR-ESI–MS peak at *m/z* 357.1334 [M+H]^+^ (calculated for [M+H]^+^ ion at 357.1333) (Additional file 1: Fig. S10). The UV absorption of rubiginone K was significantly different from rubiginone J (**1**) (Additional file 1: Fig. S22). The ^13^C-NMR data showed that compound **2** possesses two hydroxyl groups at C-7 and C-12, which is different from the two carbonyl groups at C-7 and C-12 in compound **1**. The absolute structure of rubiginone K was determined by ^1^H–^1^H COSY, HSQC, HMBC and NOESY analysis, as well as the X-ray Crystal diffraction (CDCC Deposit number: 2093018) analysis (Fig. 5b; Additional file 1: Figs. S23–S29; Tables S3, S4).

Rubiginone L (**5**) was obtained as brownish-red oil. Its molecular formula C_20_H_20_O_5_ was established upon analysis of the HR-ESI–MS peak at *m/z* 341.1390 [M+H]^+^ (calculated for [M+H]^+^ ion at 341.1384) (Additional file 1: Fig. S30). The UV spectrum of rubiginone L (Additional file 1: Fig. S22) is similar to rubiginone K. Compared to rubiginone K, rubiginone L lacks the C-2 hydroxyl group. The structure of rubiginone L was determined by ^1^H–^1^H COSY, HSQC and HMBC, as well as the X-ray Crystal diffraction (CDCC Deposit number: 2093031) analysis (Fig. 5b; Additional file 1: Figs. S31–S36; Tables S5, S6).

Rubiginone M (**7**) was obtained as brown powder. The molecular formula C_20_H_16_O_5_, was established upon analysis of the HR-ESI–MS peak at *m/z* 337.1072 [M+H]^+^ (calculated for [M+H]^+^ ion at 337.1071) (Additional file 1: Fig. S30). The molecular formula of rubiginone M is the same as compound **4**. However, from the ^1^H, ^13^C-NMR and 2D-NMR data (Additional file 1: Figs. S37–S43), it is clear that rubiginone M has a hydroxyl group at C-2 (*δ*_H_ 4.84; *δ*_C_ 78.22) position, while in compound **4** the hydroxyl group is at the C-4. The (*2S*, *3S*) configuration of rubiginone M was supported by the CD and ECD analysis (Fig. 5c; Additional file 1: Figs. S44–S45).

Rubiginone N (**8**) was obtained as brown powder. The molecular formula C_22_H_18_O_7_ was established upon analysis of the HR-ESI–MS peak at *m/z* 395.1132 [M+H]^+^ (calculated for [M+H]^+^ ion at 395.1125) (Additional file 1: Fig. S30). Rubiginone N has the same molecular formula as the known compound 4-*O*-acetyl-rubiginone D_2_ [14]. According to the NOESY spectrum (Additional file 1: Fig. S52), the hydrogen atom on C-3 of rubiginone N has correlation with the hydrogen atom at C-4, indicating that the hydroxyl group at C-4 of rubiginone N has an α configuration, while 4-*O*-acetyl-rubiginone D_2_ possesses a β configuration at C-4. The absolute structure of rubiginone N was further determined by ^1^H-^1^H COSY, HSQC and HMBC analysis (Additional file 1: Figs. S46–S51). Rubiginone N is an acetylation derivative of rubiginone J.

### Characterization of the two cytochrome P450 hydroxylases and the *O*-methyltransferase

The two cytochrome P450 hydroxylase-encoding genes, *rubN1* and *rubN2*, and the gene encoding a putative *O*-methyltransferase, *rubM4*, were individually inactivated by in-frame deletion [26], to generate mutant strains Z0004 (i.e., CB02414Δ*rubN1*), Z0005 (i.e., CB02414Δ*rubN2*), and Z0008 (i.e., CB02414Δ*rubM4*) (Additional file 1: Fig. S53). The respective genes were cloned into the pSET152 plasmid under the ermE^*^ promoter and introduced into the respective gene-deletion mutants for complementation, to generate mutant strains Z0006 (for *rubN1* complementation), Z0007 (for *rubN2* complementation), and Z0009 (for *rubM4* complementation).

**Fig. 6:**
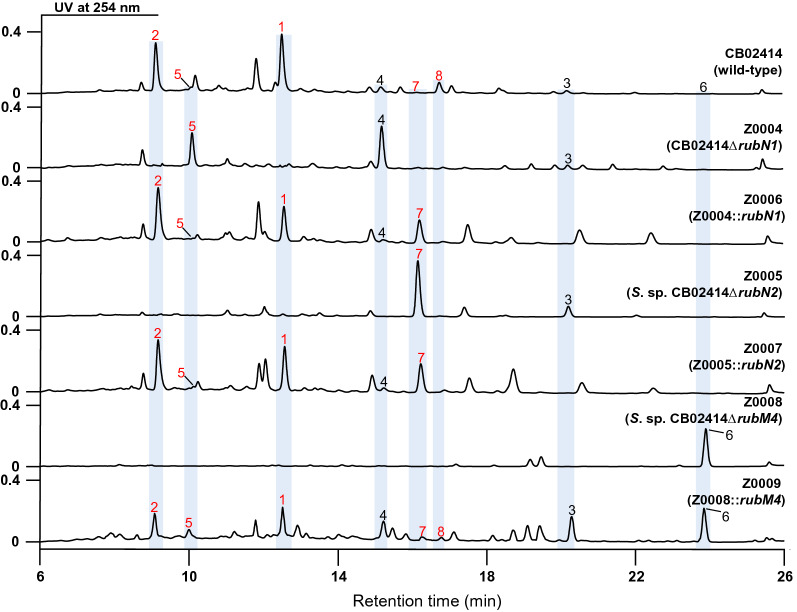
HPLC analysis of rubiginone production in *Streptomyces* sp. CB02414 (wild-type) and the Z0004–Z0007 mutants

The Z0004 mutant produced compounds **3**, **4**, **5**, and **6**, and the production of compounds **1**, **2**, **7**, and **8** was abolished. All the produced compounds in Z0004 lack the hydroxyl group at C-2, but compounds **4** and **5** still possess the C-4 hydroxyl group. When *rubN1* was introduced into Z0004, production of all the eight rubiginones was restored in the complementation strain Z0006. These results suggested that RubN1 is responsible for the introduction of the β-hydroxyl group at C-2. Similarly, the Z0005 mutant only produced compounds **3**, **6**, and **7**, which lack the C-4 hydroxyl group, and the production of the other five compounds (rubiginones J, K, A_2_, L, and N) that have the C-4 hydroxyl group was abolished. The *rubN2* complementation strain Z0007 was able to produce all the eight rubiginones. Therefore, RubN2 catalyzes the C-4 oxidation step to form a hydroxyl group with an α-configuration (Fig. 6).

The *rubM4*-deletion mutant Z0008 only produced compound **6** which contains a C-8 hydroxyl group instead of the C-8 methoxy group in the other seven compounds. In the *rubM4* complementation strain Z0009, production of all the eight rubiginones was restored. From these results, it is clear that RubM4 is responsible for the methylation of the C-8 hydroxyl group in compound **6**. Since compound **6** is the only rubiginone product of Z0008, the C-8 methylation reaction should occur in the early stage of the post-PKS tailoring steps in rubiginone biosynthesis (Fig. 6).

### Proposed biosynthetic pathway for the rubiginones in CB02414

**Fig. 7:**
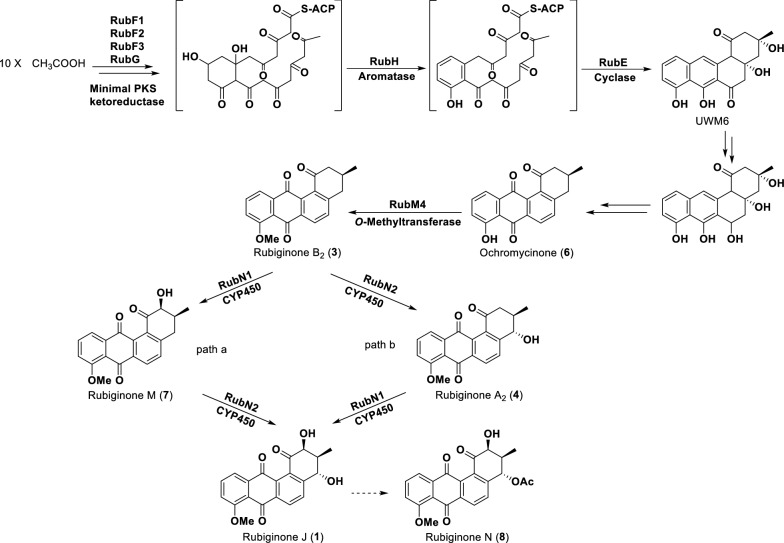
Proposed biosynthetic pathway for the rubiginones in *Streptomyces* sp. CB02414

Thanks to the early biosynthetic studies on typical angucyclines, exemplified by urdamycin, landomycin, simocyclinone, and jadomycin, biosynthesis of the benz[*a*]anthracene skeleton was well elucidated [9]. The post-PKS tailoring reactions are the major factors that generate the structural diversity of angucyclines. In the *rub* gene cluster of CB02414, the presence of two cytochrome P450 hydroxylases and one *O*-methyltransferase was key to understanding the biosynthesis of the rubiginones. Based on the structures of the eight rubiginones isolated from the CB02414 wild-type and mutants, we proposed a biosynthetic pathway for these compounds (Fig. 7).

The four proteins RubF1, RubF2, RubF3, and RubG, which represent ketoacyl synthase, chain length factor, acyl carrier protein, and a PKS-associated ketoreductase, respectively, are common in all aromatic polyketide BGCs. The aromatase RubH and cyclase RubE are homologous to other counterparts in type II PKS gene clusters [9]. It was proposed that the six proteins mentioned above are sufficient to produce UWM6, the common biosynthetic intermediate for angucyclines [9] After a few modification steps, UWM6 is converted to ochromycinone (**6**), the earliest intermediate isolated in this study. The methyltransferase RubM4 catalyzes the methylation of C-8 hydroxyl group to form rubiginone B_2_ (**3**), which is used as a substrate by the cytochrome P450 hydroxylases RubN1 and RubN2, to generate rubiginone J. There are two possible pathways for the two P450-catalyzed conversions from rubiginone B_2_ to rubiginone J: (i) in path a, RubN1 catalyzes the C-2 hydroxylation to form rubiginone M, which is used as a substrate by RubN2 to introduce the C-4 β-hydroxyl group to produce rubiginone J; (ii) in path b, rubiginone B_2_ is oxidized by RubN2 to generate compound **4**, followed by the RubN1-catalyzed transformation of compound **4** to rubiginone J. From the production profiles of the CB02414 wild-type and mutant strains, it seems that both path a and path b are applicable in CB02414 (Fig. 7). Compound **8** is an acetyl derivative of compound **1**, and it is a minor product of the CB02414 wild-type strain. It is not clear whether the acetylation of compound **1** is a spontaneous or an enzymatic reaction.

## Discussion

Actinobacteria are a rich resource of bioactive natural products, many of which have been extensively used in the clinical setting. However, most of the antibiotics used today were discovered 50 years ago and the rapid emergence of antibiotic resistance requires the discovery of new natural products with novel mode of action. Recent advances in high-throughput strain prioritization [17], next generation genome sequencing, and bioinformatics analysis [18, 27–30] have disclosed that actinobacteria possess a huge potential in producing novel secondary metabolites. Most of the BGCs in actinobacteria are silent under routine laboratory fermentation conditions. Therefore, linking BGCs to their encoded natural products (also known as the forward genome mining approach) is an important step in the discovery of novel microbial natural products. Many natural products have been identified by using the forward genome mining strategy [31].

Angucyclines have broad biological activities, including antitumor, antimicrobial, enzyme inhibition. Therefore, their discovery and understanding their structure–activity relationship has been a focus of natural product chemists. Although hundreds of members have been characterized in the past 60 years, the number of angucyclines is still growing quickly. According to our recent survey, more than 200 novel angucycline natural products have been reported in the last decade, most of which are isolated from *Streptomyces* spp. Biosynthetic studies of angucyclines are always intriguing, because of the diverse modifications brought by the post-PKS tailoring enzymes, such as oxidoreductases and glycosyltransferases [32, 33]. Compared with the complex oxidative rearrangement reactions and decorations, biosynthesis of the angucycline backbones is proposed to occur via two different biosynthetic routes, using the benz[*a*]anthracene intermediate and the anthracyclinone intermediate, respectively. The benz[*a*]anthracene intermediate is involved in the formation of landomycin A, chrysomycin A, ravidomycin V, and kinamycin D [9]. The anthracycline intermediate was observed in the biosynthesis of angucyclines PD116198 and BE7585A [9].

More than ten rubiginones have been isolated from *S. griseorubiginosus* Q144-2, *Saccharopolyspora* sp. BCC 21906, *Streptomyces* sp. Gö N1/5, *Streptomyces* sp. SNA-8073, and *Streptomyces* sp. KMC004. Besides the well-known biological activities for angucyclines, such as cytotoxicity, antibacterial, and platelet aggregation inhibition, the rubiginones are able to potentiate the cytotoxicity of vincristine (VCR) against VCR-resistant cancer cell lines. Although many rubiginones have been isolated to date, their BGCs and biosynthetic pathways were not reported. The *hrb* gene cluster was cloned and characterized in 2010 and the biosynthetic pathway for hatomarubigins was proposed. However, the post-PKS modifications of the hatomarubigins occur mainly on the D-ring. In this study, we analyzed the genome of *Streptomyces* sp. CB02414 and characterized the *rub* gene cluster that is responsible for the biosynthesis of the eight rubiginones isolated from CB02414. The rubiginones isolated from CB02414 feature different oxidative modifications on the A-ring. Based on the rubiginones produced by the CB02414 wild-type and mutant strains, we were able to propose a plausible biosynthetic pathway for the rubiginones. In this pathway, the two cytochrome P450 hydroxylases RubN1 and RubN2 introduce the α-hydroxyl group at C-2 and the β-hydroxyl group at C-4, respectively. Our attempts to overexpress *rubN1* and *rubN2* in *Escherichia coli* BL21 (DE3) failed to produce soluble proteins, thus hindering the kinetics studies of these two enzymes. We also introduced *rubN1* and *rubN2* into different *Streptomyces* hosts, including *Streptomyces lividans* TK24 and *Streptomyces albus* J1074, and tried the biotransformation of compound **3**, **4**, and **7** in the resulted strains, but no conversion was observed (data not shown). It is possible that the fed rubiginones was not able to penetrate the cell membrane of the heterologous hosts, and the biotransformation could not occur without the substrates.

It is interesting that we were able to obtain the crystals for rubiginones K and L, which helped us to establish the configurations of the hydroxyl groups or methyl group at the C-2, C-3, and C-4 of the isolated rubiginones. We also conducted crystallization of the other isolated rubiginones using different conditions, but no crystal was obtained. Considering that rubiginones K and L both possess hydroxyl groups at C-1 and C-12, this structural feature may facilitate the crystallization process. Rubiginones K and L are not stable and their C-1 and C-12 hydroxyl groups are oxidized into ketones during the purification procedure, to generate rubiginones J and A_2_, respectively. The photo-induced oxidation of C-1 hydroxyl group in rubiginones was reported before [34], we believed that the oxidation of C-12 hydroxyl group may follow a similar mechanism as the C-1 hydroxyl group. Rubiginone K was produced as a major metabolite in the CB02414 wild-type and rubiginone L was a major product of the Z0004 mutant, but it remains unclear how these two rubiginones are biosynthesized and whether they are shunt products of the rubiginone biosynthetic pathway.

## Conclusions

In this study, we first analyzed the two type II PKS gene clusters in *Streptomyces* sp. CB02414 and identified their functions through gene-inactivation experiments. We isolated eight rubiginones, including five new ones, from the CB02414 wild-type strain. Their structures were determined by the combination of HR-ESI–MS, 1D and 2D NMR, X-ray crystal diffraction, CD test, and ECD calculations. We investigated the functions of two cytochrome P450 hydroxylases (RubN1 and RubN2) in the *rub* cluster of CB02414 and confirmed that they are responsible for the introduction of the hydroxyl groups at C-2 and C-4 of rubiginones, respectively. Based on the production profiles in the CB02414 wild-type and the gene-deletion mutant strains, we proposed a biosynthetic pathway for the rubiginones. Our study enlarges the rubiginone family of natural products and lays the foundation for the generation of novel rubiginones using the combinatory biosynthesis strategy. Moreover, this study exemplifies the power of the genome mining strategy in the targeted discovery of novel microbial natural products.

## Methods

### General experimental procedures

HRMS spectra were analyzed on an LTQ-ORBITRAP-ETD instrument (Thermo Scientific, MA, USA). NMR spectra were recorded on the Varian spectrometers (400/500/600 MHz) (Brucker, Ettlingen, Germany). CD spectroscopy was measured by using JASCO (J-185, Tokyo, Japan) at room temperature (25 °C). Crystal data using Cu Kα radiation were acquired on a Rigaku APEX-II XtaLAB PRO MM007HF diffractometer at 100 K. Optical Rotatory Dispersion (ORD) spectrum was used to determine the optical activity of compounds (Rudolph Research Analytical, Autopol IV, USA). For purification of compounds, column chromatography (CC) was carried out using silica gel or Sephadex LH-20. Reversed-phase high performance liquid chromatography (RP-HPLC) was performed using a Waters 1525 Binary HPLC Pump equipped with a Welch Ultimate AQ-C18 column (250 × 10 mm, 5 μm, Welch Materials Inc., Shanghai, China) and a Waters 2489 UV/Visible Detector (Shimadzu, Kyoto, Japan).

### Bacterial strains and fermentation

*Streptomyces* sp*.* CB02414 was grown on the GYM agar plate containing per liter: 4 g yeast extract, 4 g glucose, 10 g malt extract, 2 g CaCO_3_, 20 g agar, pH 7.2) and incubated at 30 °C to obtain spores after 3–5 days. *Escherichia coli* DH5α and S17-1 were grown in liquid Luria–Bertani medium with antibiotic added (apramycin or kanamycin) and incubated at 30 °C for 12 h. The final concentration of antibiotic was 50 μg/mL.

*Streptomyces* sp. CB02414 was inoculated into 250-mL Erlenmeyer flasks containing 50 mL tryptic soy broth (TSB) medium and some glass beads, then cultivated at 30 °C on a rotary shaker at 220 rpm for 36 h. Then 10% (v/v) seed culture was inoculated into 50 mL production medium (B medium, g/L: 40 dextrin, 7.5 tomato paste, 2.5 NZ-Amine, 5 yeast extract, pH 7.2 ± 0.2; C medium, g/L: 25 glucose, 25 corn flour, 5 yeast extract, pH 7.2 ± 0.2; F medium, g/L: 100 sucrose, 10 glucose, 5 yeast extract, 0.1 casamino acids, 21 MOPS, trace elements 1 mL, 0.25 K_2_SO_4_, 1 MgCl_2_·6H_2_O, pH 7.2 ± 0.2), and then cultured for 7 days at 30 °C on a rotary shaker at 220 rpm. After fermentation for 6 days, 2% (m/v) XAD-16 resin was added into each flask and then incubated overnight on a rotary shaker at 220 rpm. For large-scale fermentation (8-L), 50 mL of seed culture was inoculated into a 2-L Erlenmeyer flask containing 500 mL of production medium and 16 flasks were used for the fermentation. After the fermentation, the crude extract was exposed to sunlight in air for 2 h, following the same method used previously, to simplify the purification steps of the photosensitive compounds [14].

### Extraction, isolation, and purification of rubiginones

For the analytical-scale fermentation, the supernatant was discarded after centrifugation (3000 rpm, 10 min) at room temperature and the resins and bacteria were air-dried for 2 days. The resins and bacteria were extracted with MeOH (30 mL × 3) and evaporated in rotary evaporator at 35 °C, further extracted three times with 30 mL ethyl acetate (EtOAc)/H_2_O (1:1, v/v), the organic phase was evaporated in rotary evaporator at 35 °C and dissolved in 1 mL MeOH for HPLC analysis. For the large-scale fermentation (8-L), the same approach was used to afford the crude extracts (6.7 g). The crude extracts were subjected to silica gel CC and eluted with a gradient of dichloromethane (DCM)/EtOAc (9:1, 7:1, 5:1, 3:1, 1:1, v/v) and a gradient of EtOAc/MeOH (8:1, 6:1, 3:1, 1:1, v/v), and pure MeOH, to give ten fractions (Fr. 1–Fr. 10).

Fr. 1 was subjected to silica gel CC and eluted with a gradient of DCM/EtOAc (40:1, 35:1, 30:1, 20:1, 10:1, 8:1, 5:1, 2:1, 1:1, v/v), then purified by Sephadex LH-20 chromatography and semi-preparative RP-C18 HPLC with a flow rate of 3 mL/min and a gradient elution of CH_3_CN/H_2_O in 15 min (10% for 3 min, 10% to 100% for 4 min, followed by 100% for 5 min, and 100% to 10% for 2 min, followed by 10% for 1 min) to obtain compound **6** (1.2 mg).

Fr. 2 was also subjected to silica gel CC and eluted with a gradient of petroleum ether (PE)/EtOAc (10:1, 6:1, 3:1, 2:1, 1:1, 1:3, v/v), and pure EtOAc, then purified by Sephadex LH-20 chromatography and semi-preparative RP-C18 HPLC with a flow rate of 3 mL/min and a gradient elution of CH_3_CN/H_2_O in 25 min (10% for 2 min, 10% to 80% for 6 min, followed by 80% for 2 min, then 80% to 10% for 14 min, followed by 10% for 1 min), to afford compound **3** (12.2 mg).

Fr. 4 were subjected to silica gel CC, with a gradient of PE/EtOAc (10:1, 8:1, 5:1, 1:1, v/v), and pure EtOAc, and a gradient of EtOAc/MeOH (10:1, 8:1, 1:1, v/v), then purified by Sephadex LH-20 chromatography and semi-preparative RP-C18 HPLC with a flow rate of 3 mL/min and a gradient elution of CH_3_CN/H_2_O in 25 min (30% for 2 min, 30% to 70% for 18 min, then 70% to 100% for 1 min, followed by 100% for 1 min, and 100% to 30% for 1 min, followed by 30% for 2 min) to obtain compounds **4** (25.6 mg), **7** (3.1 mg) and **8** (2.6 mg).

Fr. 5 were subjected to silica gel CC, with a gradient of EtOAc/MeOH (15:1, 12:1, 8:1, 6:1, 3:1, v/v), compounds **2** (3.2 mg) and **5** (1.6 mg) were purified by Sephadex LH-20 chromatography and semi-preparative RP-C18 HPLC with a flow rate of 3 mL/min and a gradient elution of CH_3_CN/H_2_O in 25 min (30% for 2 min, 30% to 50% for 16 min, then 50% to 100% for 2 min, followed by 100% for 2 min, and 100% to 30% for 1 min, followed by 30% for 2 min). Compound **1** (58.8 mg) was purified by silica gel CC, with a gradient of DCM/EtOAc (9:1, 4:1, 7:3, 1:1, 3:7, v/v), pure EtOAc, and a gradient of EtOAc/MeOH (4:1, 3:2, 3:7, v/v).

### Physicochemical data of compounds 1–8

#### Rubiginone J (1)

Light-yellow needles, [α]_D_^25^ + 120° (*c* = 0.35, MeOH); UV (MeOH) *λ*_*max*_ 212.3, 265.9, 382.7 nm (Additional file 1: Fig. S22); HR-ESI–MS *m*/z 353.1022 [M+H]^+^ (*calcd* for C_20_H_16_O_6_, 353.1020) (Additional file 1: Fig. S10). ^1^H NMR (400 MHz, CDCl_3_) *δ* 8.20 (d, *J* = 12.0 Hz, 1H, H-6), 7.73 (d, *J* = 12.0 Hz, 1H, 5-H), 7.63 (t, *J* = 12.0, 24.0 Hz, 1H, 10-H), 7.58 (dd, *J* = 1.2, 11.4 Hz, 1H, 11-H), 7.23 (dd, *J* = 2.4, 12.6 Hz, 1H, 9-H), 5.29 (d, *J* = 7.8 Hz, 1H, 2-H), 4.79 (d, *J* = 5.4 Hz, 1H, 4-H), 3.94 (s, 3H, 8-OCH_3_), 2.80 (m, 1H, 3-H), 0.82 (d, *J* = 10.8 Hz, 3H, 3-CH_3_); ^13^C NMR (101 MHz, CDCl_3_) *δ* 199.66 (C-1), 183.97 (C-12), 181.19 (C-7), 160.19 (C-8), 148.14 (C-4a), 137.47 (C-11a), 136.48 (C-6a), 135.99 (C-10), 135.45 (C-12a), 134.72 (C-5), 132.34 (C-12b), 131.72 (C-6), 120.54 (C-7a), 119.96 (C-11), 117.71 (C-9), 73.39 (C-2), 73.16 (C-4), 56.77 (C-8-OCH_3_), 45.04 (C-3), 10.85 (3-CH_3_).

#### Rubiginone K (2)

Brown oil, UV (MeOH) *λ*_*max*_ 237.5, 265.7, 288.4, 337.4 nm (Additional file 1: Fig. S22); HR-ESI–MS *m*/z 357.1334 [M+H]^+^ (*calcd* for C_20_H_20_O_6_, 357.1333) (Additional file 1: Fig. S10). ^1^H NMR (400 MHz, CD_3_OD) *δ* 8.02 (d, *J* = 8.0 Hz, 1H, H-6), 7.61 (d, *J* = 8.4 Hz, 1H, 5-H), 7.57 (t, *J* = 8.0 Hz, 1H, 10-H), 7.25 (d, *J* = 7.2 Hz, 1H, 11-H), 7.08 (d, *J* = 8.4 Hz, 1H, 9-H), 6.62 (s, 1H, 12-H), 5.33 (d, *J* = 4.4 Hz, 1H, 1-H), 4.63 (d, *J* = 7.6 Hz, 1H, 4-H), 4.15 (dd, *J* = 2.8, 4.4 Hz, 1H, 2-H), 3.88 (s, 3H, 8-OCH_3_), 1.96 (m, 1H, 3-H), 1.21 (d, *J* = 7.2 Hz, 3H, 3-CH_3_); ^13^C NMR (101 MHz, CD_3_OD) *δ* 186.12 (C-7), 161.45 (C-8), 147.86 (C-11a), 142.86 (C-6a), 146.26 (C-4a), 136.60 (C-12a), 136.13 (C-12b), 134.82 (C-10), 129.47 (C-5), 127.47 (C-6), 123.16 (C-11), 120.87 (C-7a), 112.91 (C-9), 72.69 (C-4), 72.06 (C-2), 68.87 (C-1), 65.18 (C-12), 56.38 (C-8-OCH_3_), 41.92 (C-3), 14.77 (3-CH_3_).

#### Rubiginone B_2_ (3)

Light yellow needles, UV (MeOH) *λ*_*max*_ 264.7, 377.9 nm (Additional file 1: Fig. S22); ^1^H NMR (400 MHz, CDCl_3_) and ^13^C NMR (101 MHz, CDCl_3_) see Additional file 1: Figs. S3, S4; HR-ESI–MS *m*/z 321.1135 [M+H]^+^ (*calcd* for C_20_H_16_O_4_, 321.1121) (Additional file 1: Fig. S10).

#### Rubiginone A_2_ (4)

Yellow needles, UV (MeOH) *λ*_*max*_ 264.7, 381.5 nm (Additional file 1: Fig. S22); ^1^H NMR (400 MHz, DMSO-*d*_6_) and ^13^C NMR (101 MHz, DMSO-*d*_6_) see Additional file 1: Figs. S5, S6; HR-ESI–MS *m*/z 337.1077 [M+H]^+^ (*calcd* for C_20_H_16_O_5_, 337.1071) (Additional file 1: Fig. S10).

#### Rubiginone L (5)

Brownish-red oil, UV (MeOH) *λ*_*max*_ 224.5, 263.4, 292.0, 336.2 nm (Additional file 1: Fig. S22); HR-ESI–MS *m*/z 341.1390 [M+H]^+^ (*calcd* for C_20_H_20_O_5_, 341.1384) (Additional file 1: Fig. S30). ^1^H NMR (500 MHz, CD_3_OD) *δ* 8.03 (d, *J* = 8.0 Hz, 1H, H-6), 7.72 (d, *J* = 8.0 Hz, 1H, 5-H), 7.60 (t, *J* = 8.0 Hz, 1H, 10-H), 7.25 (d, *J* = 7.5 Hz, 1H, 11-H), 7.12 (d, *J* = 8.5 Hz, 1H, 9-H), 6.51 (s, 1H, 12-H), 5.49 (t, *J* = 7.0 Hz, 1H, 1-H), 4.35 (d, *J* = 9.5 Hz, 1H, 4-H), 3.91 (s, 3H, 8-OCH_3_), 2.36 (m, 1H, 2-H), 1.73 (m, 1H, 3-H), 1.60 (m, 1H, 2-H), 1.19 (d, *J* = 7.0 Hz, 3H, 3-CH_3_); ^13^C NMR (126 MHz, CD_3_OD) *δ* 186.33 (C-7), 161.49 (C-8), 148.53 (C-4a), 147.72 (C-11a), 141.67 (C-12a), 138.51 (C-12b), 136.13 (C-10), 134.51 (C-6a), 127.74 (C-5), 127.37 (C-6), 123.18 (C-11), 121.02 (C-7a), 113.06 (C-9), 75.11 (C-4), 66.61 (C-1), 65.25 (C-12), 56.41 (C-8-OCH_3_), 40.15 (C-2), 37.24 (C-3), 19.38 (3-CH_3_).

#### Ochromycinoe (6)

Yellow powder, UV (MeOH) *λ*_*max*_ 265.9, 399.6 nm (Additional file 1: Fig. S22); ^1^H NMR (500 MHz, CDCl_3_) and ^13^C NMR (126 MHz, CDCl_3_) see Additional file 1: Figs. S7, S8; HR-ESI–MS *m*/z 307.0959 [M+H]^+^ (*calcd* for C_19_H_14_O_4_, 307.0965) (Additional file 1: Fig. S30).

#### Rubiginone M (7)

Brown powder, [α]_D_^25^ + 130° (*c* = 0.135, MeOH); UV (MeOH) *λ*_*max*_ 265.9, 379.1 nm (Additional file 1: Fig. S22); HR-ESI–MS *m*/z 337.1072 [M+H]^+^ (*calcd* for C_20_H_16_O_5_, 337.1071) (Additional file 1: Fig. S30). ^1^H NMR (600 MHz, CD_3_OD) *δ* 8.24 (d, *J* = 8.4 Hz, 1H, H-6), 7.80 (dd, *J* = 7.8, 8.4 Hz, 1H, 10-H), 7.65 (t, *J* = 7.2, 15.0 Hz, 1H, 11-H), 7.65 (t, *J* = 7.2, 15.0 Hz, 1H, 5-H), 7.51 (d, *J* = 8.4 Hz, 1H, 9-H), 4.84 (d, *J* = 4.2 Hz, 1H, 2-H), 4.01 (s, 3H, 8-OCH_3_), 3.41 (dd, *J* = 4.8, 17.4 Hz, 1H, 4-H), 3.01 (dd, *J* = 4.2, 18.0 Hz, 1H, 4-H), 2.66 (m, 1H, 3-H), 0.95 (d, *J* = 7.2 Hz, 3H, 3-CH_3_); ^13^C NMR (151 MHz, CD_3_OD) *δ* 201.07 (C-1), 185.42 (C-12), 182.69 (C-7), 161.39 (C-8), 150.34 (C-4a), 138.78 (C-11a), 136.98 (C-10), 136.86 (C-6a), 136.06 (C-12a), 135.29 (C-5), 134.99 (C-12b), 130.80 (C-6), 121.41 (C-7a), 120.16 (C-11), 119.04 (C-9), 78.22 (C-2), 55.90 (C-8-OCH_3_), 39.04 (C-3), 36.13 (C-4), 13.73 (3-CH_3_).

#### Rubiginone N (8)

Brown powder, [α]_D_^25^ + 112° (*c* = 0.05, MeOH); UV (MeOH) *λ*_*max*_ 264.7, 382.7 nm (Additional file 1: Fig. S22); HR-ESI–MS *m*/z 395.1132 [M+H]^+^ (*calcd* for C_22_H_18_O_7_, 395.1125) (Additional file 1: Fig. S30). ^1^H NMR (500 MHz, CD_3_OD) *δ* 8.36 (d, *J* = 8.0 Hz, 1H, H-6), 7.83 (m, 1H, 5-H), 7.83 (m, 1H, 11-H), 7.69 (d, *J* = 7.5 Hz, 1H, 10-H), 7.54 (d, *J* = 8.5 Hz, 1H, 9-H), 6.08 (d, *J* = 3.0 Hz, 1H, 4-H), 5.16 (d, *J* = 4.5 Hz, 1H, 2-H), 4.02 (s, 3H, 8-OCH_3_), 2.76 (m, 1H, 3-H), 2.13 (s, 1H, 4-OCOCH_3_), 0.95 (d, *J* = 7.0 Hz, 3H, 3-CH_3_); ^13^C NMR (126 MHz, CD_3_OD) *δ* 199.59 (C-1), 185.02 (C-12), 182.24 (C-7), 171.78 (4-OCOCH_3_), 161.54 (C-8), 145.65 (C-4a), 138.68 (C-11a), 137.92 (C-10), 137.16 (C-6a), 136.82 (C-12a), 135.90 (C-5), 135.72 (C-12b), 131.61 (C-6), 121.48 (C-7a), 120.21 (C-11), 119.21 (C-9), 75.35 (C-2), 74.79 (C-4), 56.95 (C-8-OCH_3_), 44.22 (C-3), 20.94 (4-OCOCH_3_), 11.10 (3-CH_3_).

### Gene inactivations in *Streptomyces* sp. CB02414

A pOJ260-based plasmid Y0002 was constructed to generate the Δ*rubF1/rubF2* replacement mutant in CB02414 via a double crossover homologous recombination. To inactivate *rubF1/rubF2*, a partial fragment spanning these two genes was replaced with the kanamycin-resistance gene using the Seamless Cloning and Assembly kit (TSINGKE, China), and the mutated *rubF1/rubF2* was cloned into pOJ260 between the *Xba*I and *Hind*III restriction sites. This plasmid was introduced into CB02414 (wild-type) and Z0001 (i.e., CB02414Δ*orf3/orf4*) by intergeneric conjugation, then selected for kanamycin-resistance and apramycin-sensitive phenotype to obtain the desired double-crossover mutants Z0002 (i.e., CB02414Δ*rubF1/rubF2*) and Z0003 (i.e., Z0001Δ*rubF1/rubF2*), respectively. Deletion of *orf3/orf4*, *rubN1*, *rubN2* and *rubM4* in CB02414 was conducted via in-frame deletion, to obtain mutants Z0001, Z0004, Z0005 and Z0008, respectively. The mutants were confirmed by PCR analysis and DNA sequencing. The strains and plasmids were shown in Additional file 1: Table S9, the PCR primers were shown in Additional file 1: Table S10, schematic diagram for the gene inactivations and PCR confirmation of the mutant strains were shown in Additional file 1: Figs. S1, S53, respectively.

### Complementation in Z0004, Z0005 and Z0008.

For the complementation of *rubN1*, a pSET152-based plasmid Y0005, in which *rubN1* was cloned under the control of the constitutive promoter ermE^*^ was constructed. The *rubN1* was PCR-amplified by the Q5 high-fidelity DNA polymerase using genomic DNA of CB02414 wild-type as template, and the resultant PCR fragment was inserted into the pSET152 between *Xba*I and *Bam*HI restriction sites. This plasmid Y0005 was then introduced into Z0004 (i.e., CB02414Δ*rubN1*) by intergeneric conjugation and selected for apramycin resistant conjugants to afford Z0006 (i.e., Z0004::*rubN1*). The complementation strains Z0007 (i.e., Z0005::*rubN2*) and Z0009 (i.e., Z0008::*rubM4*) were obtained by using the same method. The PCR primers used in the complementation experiments are shown in Additional file 1: Table S10.

## Supplementary Information

**Additional file 1. **Additional Tables S1–S10, Figures S1–S53.

## Data Availability

All data generated or analyzed during this study are included in this published article.
