# Supplementary material for: Genome mining of novel rubiginones from Streptomyces sp. CB02414 and characterization of the post-PKS modification steps in rubiginone biosynthesis
Source: Microb Cell Fact. 2021 Oct 2;20:192. doi: 10.1186/s12934-021-01681-5 (PMC8487521; doi:10.1186/s12934-021-01681-5)
Supplement: Supplementary file 1 — Additional file 1. Additional Tables S1–S10, Figures S1–S53. [file 12934_2021_1681_MOESM1_ESM.docx]

**Additional Information**

**Genome mining of novel rubiginones from *Streptomyces* sp. CB02414 and characterization of the post-PKS oxidation steps in rubiginone biosynthesis**

Jingyan Zhang^1^, Ying Sun^2^, Yeji Wang^1^, Xin Chen^1^, Lu Xue^2^, Jingjing Zhang^2^, Xiangcheng Zhu^1,3,4^, Yanwen Duan^1,3,4*^, Xiaohui Yan^1,2*^

^1^Xiangya International Academy of Translational Medicine, Central South University, Changsha, Hunan, China

^2^State Key Laboratory of Component-based Chinese Medicine, Tianjin University of Traditional Chinese Medicine, Tianjin, China

^3^Hunan Engineering Research Center of Combinatorial Biosynthesis and Natural Product Drug Discovery, Changsha, Hunan, China

^4^National Engineering Research Center of Combinatorial Biosynthesis for Drug Discovery, Changsha, Hunan, China

*To whom correspondents should be addressed: Prof. Xiaohui Yan, Central South University, Tongzipo Road，#172，Yuelu District，Changsha, Hunan 410013，China. Tel: (86) 731 8265 0539; Fax: (86) 731 8265 0551; Email: yanxh@tjutcm.edu.cn or Prof. Yanwen Duan, Email: ywduan66@sina.com.

**Table S1.** The antiSMASH analysis of *Streptomyces* sp. CB02414.

| **NO.** | **Cluster type** | **Predicted product** | **Gene similarity (%)** | **Gene cluster location** |
| --- | --- | --- | --- | --- |
| 1 | Ectoine | Ectoine | 100 | 76207–86605 |
| 2 | Melanin | Melanin | 60 | 964785–975252 |
| 3 | Siderophore | Desferrioxamine B | 83 | 1061432–1073204 |
| 4 | NRPS | Bleomycin | 9 | 1245333–1293712 |
| 5 | T II PKS | Spore pigment | 66 | 65768–138313 |
| 6 | Terpene | Albaflavenone | 100 | 178233–199255 |
| 7 | Lantipeptide-class-i | Planosporicin | 80 | 988630–1013311 |
| 8 | Lantipeptide-class-v | Zorbamycin | 6 | 344004–390012 |
| 9 | NRPS | Coelichelin | 100 | 95223–146124 |
| 10 | Redox-cofactor | / | / | 273477–296251 |
| 11 | Lantipeptide-class-iii | SapB | 100 | 558691–581273 |
| 12 | Terpene | Hopene | 92 | 78301–104171 |
| 13 | Terpene | Versipelostatin | 5 | 398683–419714 |
| 14 | RiPP-like | Informatipeptin | 28 | 435695–445910 |
| 15 | T I PKS, butyrolactone | C-1027 | 32 | 85490–131378 |
| 16 | Terpene | 2-methylisoborneol | 100 | 216948–234814 |
| 17 | Indole | 5-isoprenylindole-3-carboxylate β-D-glycosyl ester | 23 | 458091–479218 |
| 18 | Terpene | Isorenieratene | 63 | 539755–574642 |
| 19 | Siderophore | Grincamycin | 5 | 53913–67066 |
| 20 | T II PKS, lanthipeptide-class-i | Lugdunomycin | 70 | 159573–232064 |
| 21 | T III PKS | Herboxidiene | 8 | 220885-261976 |
| 22 | Siderophore | / | / | 57938–69674 |
| 23 | NRPS | Coelibactin | 100 | 136428–190557 |
| 24 | RiPP-like | / | / | 9438–20775 |
| 25 | Terpene | Geosmin | 100 | 37534–59714 |
| 26 | Lantipeptide-class-i | Tetrocarcin A | 4 | 69679–91834 |
| 27 | T I PKS | Carbapenem MM455 | 6 | 24859–72235 |

**Table S2.** ^1^H (400 MHz) and ^13^C (101 MHz) NMR data of compound **1** in CDCl_3_.


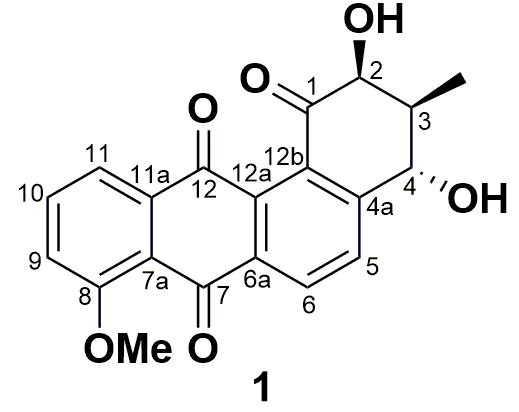


| **Rubiginone J (1)** | | | |
| --- | --- | --- | --- |
| **Position** | ***δ*_C_, type** | ***δ*_H_, mult. (*J* in Hz)** | **HMBC** |
| 1 | 199.66, C |  |  |
| 2 | 73.39, CH | 5.29, d (7.8) | 1, 3, 4, 3-CH_3_, 12b |
| 3 | 45.04, CH | 2.80, m | 1, 2, 3-CH_3_, 4a |
| 3-CH_3_ | 10.85, CH_3_ | 0.82, d (10.8) | 3, 4 |
| 4 | 73.16, CH | 4.79, d (5.4) | 1, 3, 3-CH_3_, 4a, 12b |
| 4a | 148.14, C |  |  |
| 5 | 134.72, CH | 7.73, d (12.0) | 1, 4, 4a |
| 6 | 131.72, CH | 8.20, d (12.0) | 4a, 5, 7 |
| 6a | 136.48, C |  |  |
| 7 | 181.19, C |  |  |
| 7a | 120.54, C |  |  |
| 8 | 160.19, C |  |  |
| 8-OCH_3_ | 56.77, OCH_3_ | 3.94, s | 9 |
| 9 | 117.71, CH | 7.23, dd (2.4, 12.6) | 7, 8, 11, 11a |
| 10 | 135.99, CH | 7.63, t (12.0, 24.0) | 8, 11a |
| 11 | 119.96, CH | 7.58, dd (1.8, 11.4) | 7a, 8, 10, 12 |
| 11a | 137.47, C |  |  |
| 12 | 183.97, C |  |  |
| 12a | 135.45, C |  |  |
| 12b | 132.34, C |  |  |

**Table S3.** ^1^H (400 MHz) and ^13^C (101 MHz) NMR data of compound **2** in CD_3_OD.

**
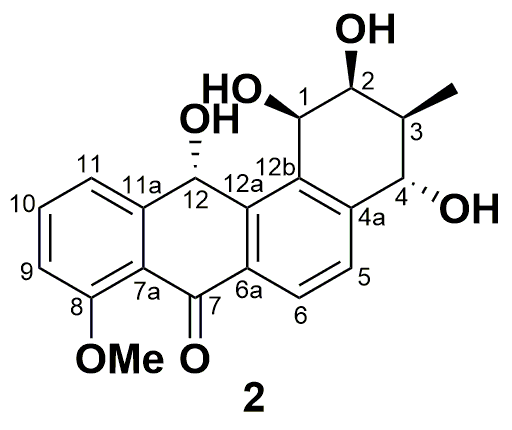
**

| **Rubiginone K (2)** | | | |
| --- | --- | --- | --- |
| **Position** | ***δ*_C_, type** | ***δ*_H_, mult. (*J* in Hz)** | **HMBC** |
| 1 | 68.87, CH | 5.33, d (4.4) | 2, 3, 4a, 12b |
| 2 | 72.06, CH | 4.15, dd (2.8, 4.4) | 1, 3, 3-CH_3_, 4, 12b |
| 3 | 41.92, CH | 1.96, m | 1, 2, 3-CH_3_, 4, 4a |
| 3-CH_3_ | 14.77, CH_3_ | 1.21, d (7.2) | 2, 3, 4 |
| 4 | 72.69, CH | 4.63, d (7.6) | 2, 3, 3-CH_3_, 4a, 5, 12b |
| 4a | 146.26, C |  |  |
| 5 | 129.47, CH | 7.61, d (8.4) | 4, 4a, 6, 6a, 7, 12b |
| 6 | 127.47, CH | 8.02, d (8.0) | 4a, 5, 6a, 7, 12, 12a |
| 6a | 142.86, C |  |  |
| 7 | 186.12, C |  |  |
| 7a | 120.87, C |  |  |
| 8 | 161.45, C |  |  |
| 8-OCH_3_ | 56.38, OCH_3_ | 3.88, s | 8, 9 |
| 9 | 112.91, CH | 7.08, d (8.4) | 7, 7a, 8, 10, 11, 11a |
| 10 | 134.82, CH | 7.57, t (8.0) | 7, 8, 9, 11, 11a |
| 11 | 123.16, CH | 7.25, d (7.2) | 7, 7a, 8, 9, 10, 11a, 12 |
| 11a | 147.86, C |  |  |
| 12 | 65.18, CH | 6.62, s | 6a, 7, 7a, 11, 11a, 12a |
| 12a | 136.60, C |  |  |
| 12b | 136.13, C |  |  |

**Table S4.** Crystal data for compound **2** (30% probability displacement ellipsoids).

| **Bond precision** | C-C = 0.0030 A | Wavelength = 1.54184 |
| --- | --- | --- |
| **Cell** | a = 9.0008 (2), b = 8.84879 (15), c = 11.4042 (2) | |
|  | alpha = 90, beta = 109.833 (3), gamma = 90 | |
| **Temperature** | 100 K | |
|  | Calculated | Reported |
| **Volume** | 854.43 (3) | 854.43 (3) |
| **Space group** | P 21 | P 1 21 1 |
| **Hall group** | P 2yb | P 2yb |
| **Moiety formula** | C_20_H_20_O_6_ | C_20_H_20_O_6_ |
| **Sum formula** | C_20_H_20_O_6_ | C_20_H_20_O_6_ |
| **Mr** | 356.36 | 356.36 |
| **Dx, g cm^-3^** | 1.385 | 1.385 |
| **Z** | 2 | 2 |
| **Mu (mm^-1^)** | 0.851 | 0.851 |
| **F000** | 376.0 | 376.0 |
| **F000´** | 377.27 | |
| **h, k, lmax** | 11, 11, 14 | 10, 10, 14 |
| **Nref** | 3448 [1841] | 3297 |
| **Tmin, Tmax** | 0.895, 0.918 | 0.827, 1.000 |
| **Tmin´** | 0.895 | |
| Correction method = # Reported T Limits: Tmin = 0.827, Tmax =1.000 | | |
| AbsCorr = MULTI-SCAN | | |
| Data completeness = 1.79/0.96 Theta (max) = 73.577 | | |
| *R* (reflections) = 0.0278 ( 3270) wR2 (reflections) = 0.0760 ( 3297) | | |
| **S =** 1.059 | Npar = 247 | |
| **CCDC** | 2093018 | |

**Table S5.** ^1^H (500 MHz) and ^13^C (126 MHz) NMR data of compound **5** in CD_3_OD.

**
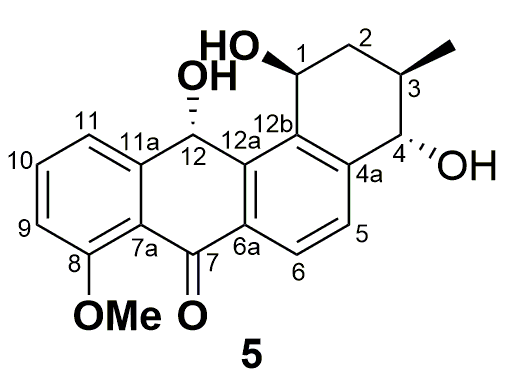
**

| **Rubiginone L (5)** | | | |
| --- | --- | --- | --- |
| **Position** | ***δ*_C_, type** | ***δ*_H_, mult. (*J* in Hz)** | **HMBC** |
| 1 | 66.61, CH | 5.49, t (7.0) | 2, 3, 4a, 12b |
| 2 | 40.15, CH_2_ | 2.36, m; 1.60, m | 1, 3, 3-CH_3_, 4, 12b |
| 3 | 37.24, CH | 1.73, m | 1, 2, 3-CH_3_, 4, 4a |
| 3-CH_3_ | 19.38, CH_3_ | 1.19, d (7.0) | 2, 3, 4 |
| 4 | 75.11, CH | 4.35, d (9.5) | 3, 3-CH_3_, 4a, 5, 12b |
| 4a | 148.53, C |  |  |
| 5 | 127.74, CH | 7.72, d (8.0) | 4, 6, 6a, 12b |
| 6 | 127.37, CH | 8.03. d (8.0) | 4a, 5, 7, 7a, 12, 12a |
| 6a | 134.51, C |  |  |
| 7 | 186.33, C |  |  |
| 7a | 121.02, C |  |  |
| 8 | 161.49, C |  |  |
| 8-OCH_3_ | 56.41, OCH_3_ | 3.91, s | 8, 9 |
| 9 | 113.06, CH | 7.12, d (8.5) | 7, 7a, 8, 10, 11, 11a |
| 10 | 136.13, CH | 7.60, t (8.0) | 7, 7a, 8, 9, 11, 11a |
| 11 | 123.18, CH | 7.25, d (7.5) | 7, 7a, 8, 9, 10, 11a, 12 |
| 11a | 147.72, C |  |  |
| 12 | 65.25, CH | 6.51, s | 6a, 7a, 11, 11a, 12a |
| 12a | 141.67, C |  |  |
| 12b | 138.51, C |  |  |

**Table S6** Crystal data for compound **5** (30% probability displacement ellipsoids).

| **Bond precision** | C-C = 0.0039 A | Wavelength = 1.54184 |
| --- | --- | --- |
| **Cell** | a = 8.0351 (2), b = 14.7742 (4), c = 27.5878 (9) | |
|  | alpha = 90, beta = 90, gamma = 90 | |
| **Temperature** | 293 K | |
|  | Calculated | Reported |
| **Volume** | 3275.01 (16) | 3275.01 (17) |
| **Space group** | P 21 21 21 | P 21 21 21 |
| **Hall group** | P 2ac 2ab | P 2ac 2ab |
| **Moiety formula** | C_20_H_20_O_5_ | C_20_H_20_O_5_ |
| **Sum formula** | C_20_H_20_O_5_ | C_20_H_20_O_5_ |
| **Mr** | 340.36 | 340.36 |
| **Dx, g cm^-3^** | 1.381 | 1.381 |
| **Z** | 8 | 8 |
| **Mu (mm^-1^)** | 0.814 | 0.814 |
| **F000** | 1440.0 | 1440.0 |
| **F000´** | 1444.71 | |
| **h, k, lmax** | 10, 18, 34 | 10, 18, 33 |
| **Nref** | 6625 [3752] | 6284 |
| **Tmin, Tmax** | 0.907, 0.937 | 0.795, 1.000 |
| **Tmin´** | 0.907 | |
| Correction method = # Reported T Limits: Tmin = 0.795, Tmax = 1.000 | | |
| AbsCorr = MULTI-SCAN | | |
| Data completeness = 1.67/0.95 Theta (max) = 73.916 | | |
| R (reflections) = 0.0401 ( 5818) wR2 (reflections) = 0.1074 ( 6284) | | |
| **S** = 1.023 | Npar = 464 | |
| **CCDC** number | 2093031 | |

**Table S7.** ^1^H (600 MHz) and ^13^C (151 MHz) NMR data of compound **7** in CD_3_OD.


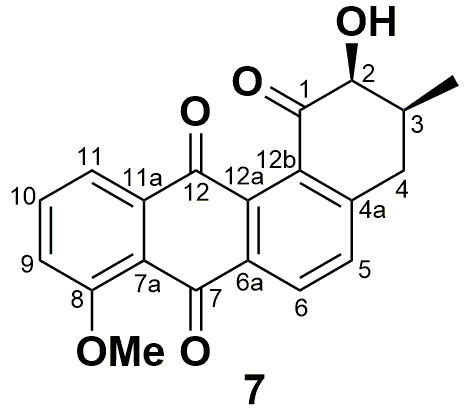


| **Rubiginone M (7)** | | | |
| --- | --- | --- | --- |
| **Position** | ***δ*_C_, type** | ***δ*_H_, mult. (*J* in Hz)** | **HMBC** |
| 1 | 201.07, C |  |  |
| 2 | 78.22, CH | 4.84, d (4.2) | 1, 3, 3-CH_3_, 4 |
| 3 | 39.04, CH | 2.66, m | 2, 4, 4a |
| 3-CH_3_ | 13.73, CH_3_ | 0.95, d (7.2) | 2, 3, 4 |
| 4 | 36.13, CH_2_ | 3.41, dd (4.8, 17.4); 3.01, dd (4.2, 18.0) | 1, 2, 3, 3-CH_3_, 4a, 5 |
| 4a | 150.34, C |  |  |
| 5 | 135.29, CH | 7.65, t (7.2, 15.0) | 4, 6a, 12b |
| 6 | 130.80, CH | 8.24, d (8.4) | 4a, 12, 12a |
| 6a | 136.86, C |  |  |
| 7 | 182.69, C |  |  |
| 7a | 121.41, C |  |  |
| 8 | 161.39, C |  |  |
| 8-OCH_3_ | 55.90, OCH_3_ | 4.01, s | 8, 11 |
| 9 | 119.04, CH | 7.51, d (8.4) | 6a, 10 |
| 10 | 136.98, CH | 7.80, dd (7.8, 8.4) | 8, 11, 11a, 12, 12a |
| 11 | 120.16, CH | 7.65, t (7.2, 15.0) | 1, 4, 4a, 7a, 9, 12 |
| 11a | 138.78, C |  |  |
| 12 | 185.42, C |  |  |
| 12a | 136.06, C |  |  |
| 12b | 134.99, C |  |  |

**Table S8.** ^1^H (500 MHz) and ^13^C (126 MHz) NMR data of compound **8** in CD_3_OD.

**
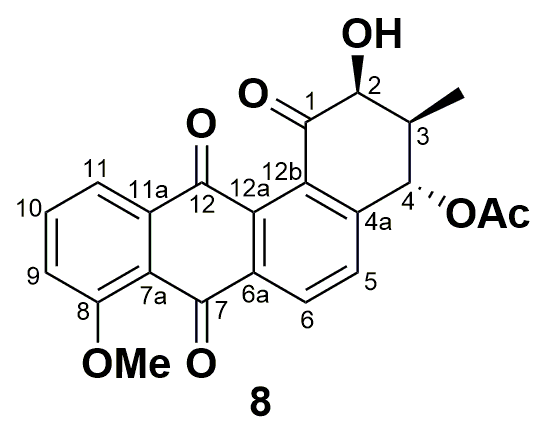
**

| **Rubiginone N (8)** | | | |
| --- | --- | --- | --- |
| **Position** | ***δ*_C_, type** | ***δ*_H_, mult. (*J* in Hz)** | **HMBC** |
| 1 | 199.59, C |  |  |
| 2 | 75.35, CH | 5.16, d (4.5) | 1, 3, 3-CH_3_, 4 |
| 3 | 44.22, CH | 2.76, m | 3-CH_3_ |
| 3-CH_3_ | 11.10, CH_3_ | 0.95, d (7.0) | 3, 4 |
| 4 | 74.79, CH | 6.08, d (3.0) | 2, 3, 3-CH_3_, 4-OCOCH_3_, 4a, 5 |
| 4-OCOCH_3_ | 171.78, C |  |  |
| 4-OCOCH_3_ | 20.94, CH_3_ | 2.13, s | 4, 4-OCOCH_3_, 4a |
| 4a | 145.65, C |  |  |
| 5 | 135.90, CH | 7.83, m | 4, 6, 6a |
| 6 | 131.61, CH | 8.36, d (8.0) | 4a, 7, 12a |
| 6a | 137.16, C |  |  |
| 7 | 182.24, C |  |  |
| 7a | 121.48, C |  |  |
| 8 | 161.54, C |  |  |
| 8-OCH_3_ | 56.95, OCH_3_ | 4.02, s | 8, 9 |
| 9 | 119.21, CH | 7.54, d (8.5) | 7a, 8 |
| 10 | 137.92, CH | 7.69, d (7.5) | 8, 11, 12 |
| 11 | 120.21, CH | 7.83, m | 8, 10 |
| 11a | 138.68, C |  |  |
| 12 | 185.02, C |  |  |
| 12a | 136.82, C |  |  |
| 12b | 135.72, C |  |  |

**Table S9.** The strains and plasmids used in this study.

| **Strains/Plasmids** | **Genotype description** | **Source** |
| --- | --- | --- |
| ***Streptomyces* strains** |  |  |
| *Streptomyces* sp. CB02414 | wild-type | This work |
| Z0001 | *orf3/orf4* gene replacement mutant strain of CB02414 | This work |
| Z0002 | *rubF1*/*rubF2* gene inactivation mutant strain of CB02414 | This work |
| Z0003 | *rubF1*/*rubF2* gene replacement mutant strain of Z0001 | This work |
| Z0004 | *rubN1* gene inactivation mutant strain of CB02414 | This work |
| Z0005 | *rubN2* gene inactivation mutant strain of CB02414 | This work |
| Z0006 | *rubN1* mutation complement of Z0004 | This work |
| Z0007  Z0008 | *rubN2* mutation complement of Z0005  *rubM4* gene inactivation mutant strain of CB02414 | This work  This work |
| Z0009 | *rubM4* mutation complement of Z0008 | This work |
|  |  |  |
| ***E. coli* strains** |  |  |
| DH5α | *E. coli* host for general cloning | Commercial |
| S17-1 | *E*. *coli*-*Streptomyces* intergeneric conjugation | Commercial |
|  |  |  |
| **Plasmids** |  |  |
| pOJ260 | *E. coli-Streptomyces* shuttle vector | Commercial |
| pSET152 | *E. coli-Streptomyces* shuttle vector | Commercial |
| Y0001 | pOJ260 derived plasmid for inactivation of *orf3/orf4* in CB02414 | This work |
| Y0002 | pOJ260 derived plasmid for inactivation of *rubF1*/*rubF2* in CB02414/Z0001 | This work |
| Y0003 | pOJ260 derived plasmid for inactivation of *rubN1* in CB02414 | This work |
| Y0004 | pOJ260 derived plasmid for inactivation of *rubN2* in CB02414 | This work |
| Y0005 | pSET152 derived plasmid for complement of *rubN1* in Z0003 | This work |
| Y0006 | pSET152 derived plasmid for complement of *rubN2* in Z0004 | This work |
| Y0007 | pOJ260 derived plasmid for inactivation of *rubM4* in CB02414 | This work |
| Y0008 | pSET152 derived plasmid for complement of *rubM4* in Z0008 | This work |

**Table S10.** The primers used in this study.

| **Name** | **Sequence (from 5´ to 3´)** | **Function** |
| --- | --- | --- |
| Y0001-up-F | GACGGCCAGTGCCAAGCTTGGCCGGATGACGGTCTCTC | Construction of Y0001 |
| Y0001-up-R | GAGTGCCGTACGGGACAGCAGCCCCCAGAACTG |  |
| Y0001-down-F | CTGCTGTCCCGTACGGCACTCGTCCTGG |  |
| Y0001-down-R | GCGGCCGCGGATCCTCTAGAGGATGTCGATGCCCTCCTC |  |
| Y0002-up-F | CGACGGCCAGTGCCAAGCTTGGCAAGGTGGTCTGCTTGTAC | Construction of Y0002 |
| Y0002-up-R | GTTCTTCTGAGTTACGACATCGATCTGGTCAC |  |
| Y0002*-*kana*-*F | ATGTCGTAACTCAGAAGAACTCGTCAAGAAGG |  |
| Y0002-kana*-*R | CGTCGTCACCCTGTGGAATGTGTGTCAGTTAG |  |
| Y0002-down-F | CATTCCACAGGGTGACGACGACTCGG |  |
| Y0002-down-R | GCGGCCGCGGATCCTCTAGAGTTCCCTTGTCGAGTTGAC |  |
| Y0003-up-F | CAGTGCCAAGCTTGGCCGGCGATGACCGCCGCGTC | Construction of Y0003 |
| Y0003-up-R | CTACCAGGTGGTGAAGTCGGTGTGGGGAACC |  |
| Y0003-down-F | CACCGACTTCACCACCTGGTAGTTCTCCCGGTAC |  |
| Y0003-down-R | CGCGGCCGCGGATCCTCTAGATCAGCGCGACCGTCG |  |
| Y0004-up-F | CGACGGCCAGTGCCAAGCTTACCACTCGGGGGTCTCC | Construction of Y0004 |
| Y0004-up-R | CCCCGCATTTCGCAACATCTACGGGCTCTTCAC |  |
| Y0004-down-F | GTAGATGTTGCGAAATGCGGGGGCTTCGG |  |
| Y0004-down-R | GCGGCCGCGGATCCTCTAGAAGCGGCATGGCGAGGG |  |
| Y0005-F | CTTGGGCTGCAGGTCGACTCTAGACTACCAGGTGACCGGTAGTTCACG | Construction of Y0005 |
| Y0005-R | TCGTGCCGGTTGGTAGGATCCCGCGAGGCGGTCCGGAC |  |
| Y0006-F | CTTGGGCTGCAGGTCGACTCTAGATCACCACTCCACCGGGAG | Construction of Y0006 |
| Y0006-R | CGTGCCGGTTGGTAGGATCCTTGTCAAAGGTTTGCGAAGTCG |  |
| Y0007-up-F | CGACGGCCAGTGCCAAGCTTTCGTACTCGCTCCGGTCAC | Construction of Y0007 |
| Y0007-up-R | CCTTGTGACCGATGCTGGGGGTTCGTTGCGTTC |  |
| Y0007-down-F | CAACGAACCCCCAGCATCGGTCACAAGGACCGTTGA |  |
| Y0007-down-R | GCGGCCGCGGATCCTCTAGAGGTGGTGATCAGCGTCTCG |  |
| Y0008-F | GGCTGCAGGTCGACTCTAGATCAACGGTCCTTGTGACCGAT | Construction of Y0008 |
| Y0008-R | CGTGCCGGTTGGTAGGATCCCCATGAACGCAACGAACC |  |

**Fig. S1.** Confirmation of gene inactivation in Z0001–Z0003. (**a**) Gene inactivation of *orf3*/*orf4*. (**b**) Gene inactivation of *rubF1*/*rubF2*.


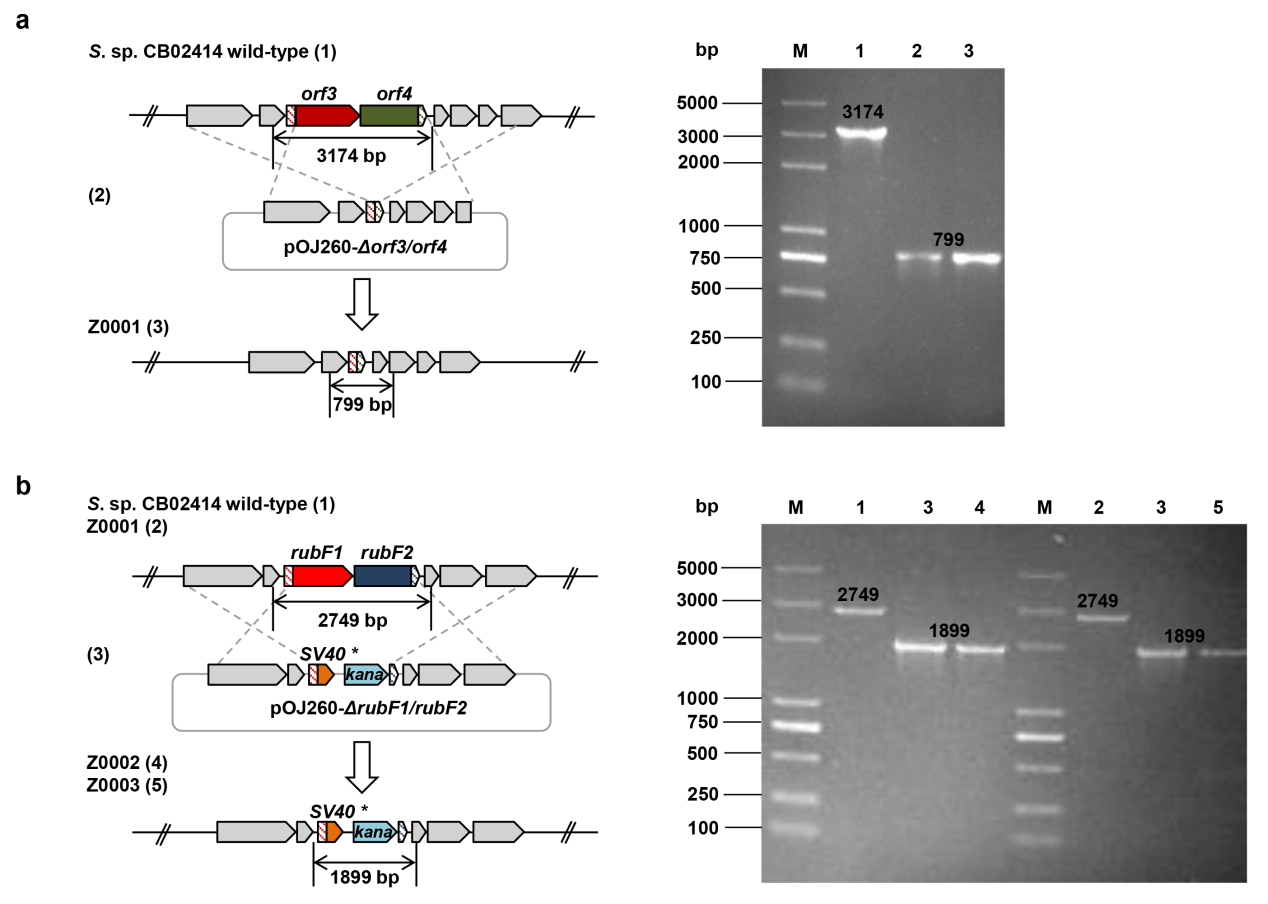


(**a**) M: DNA marker; amplification product of *orf3*/*orf4* from the CB02414 wild-type (**1**); the amplicon using plasmid Y0001 as a template (**2**); amplification product using the genomic DNA of Z0001 as a template (**3**).

(**b**) M: DNA marker; amplification product of *rubF1*/*rubF2* from CB02414 wild-type (**1**); amplification product of *rubF1*/*rubF2* from Z0001 (**2**); the amplicon using plasmid Y0002 as a template (**3**); amplification product using the genomic DNA of Z0002 as a template (**4**); amplification product using the genomic DNA of Z0003 as a template (**5**).

**Fig. S2.** Comparison of the metabolic profiles of *Streptomyces* sp. CB02414 in different fermentation media (B/C/F).


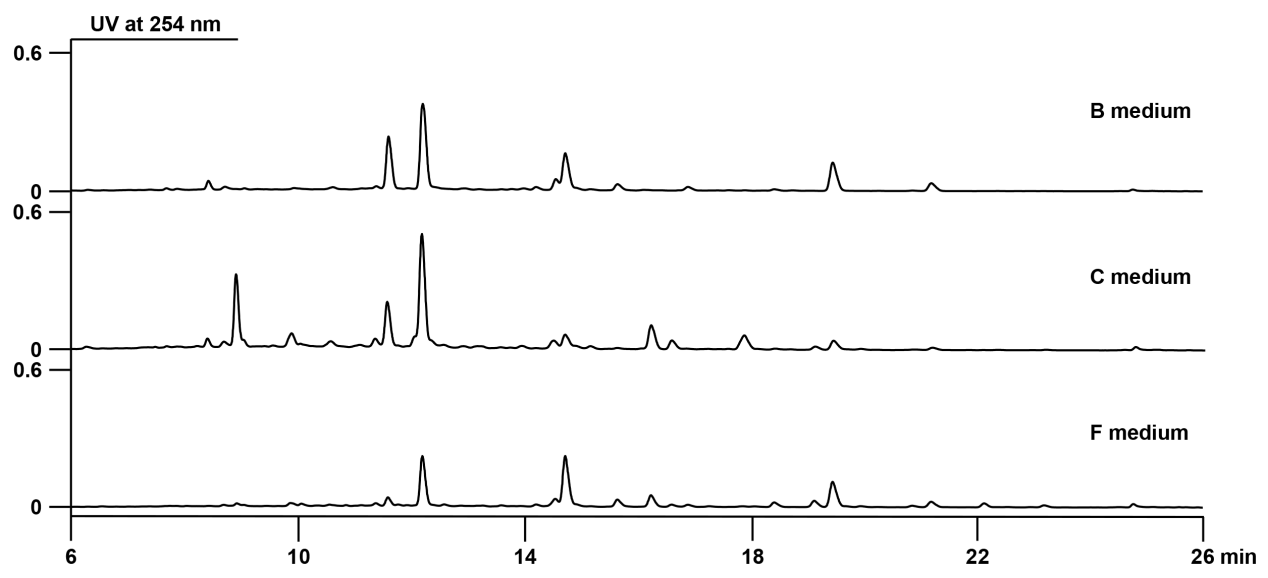


**Fig. S3.** ^1^H NMR spectrum of compound **3** in CDCl_3_ (400 MHz).

**Fig. S4.** ^13^C NMR spectrum of compound **3** in CDCl_3_ (101 MHz).

**Fig. S5.** ^1^H NMR spectrum of compound **4** in DMSO-*d_6_* (400 MHz).

**Fig. S6.** ^13^C NMR spectrum of compound **4** in DMSO-*d_6_* (101 MHz).

**Fig. S7.** ^1^H NMR spectrum of compound **6** in CDCl_3_ (500 MHz).

**Fig. S8.** ^13^C NMR spectrum of compound **6** in CDCl_3_ (126 MHz).

**Fig. S9.** The peak marked with an asterisk converted into compound **1**.

**
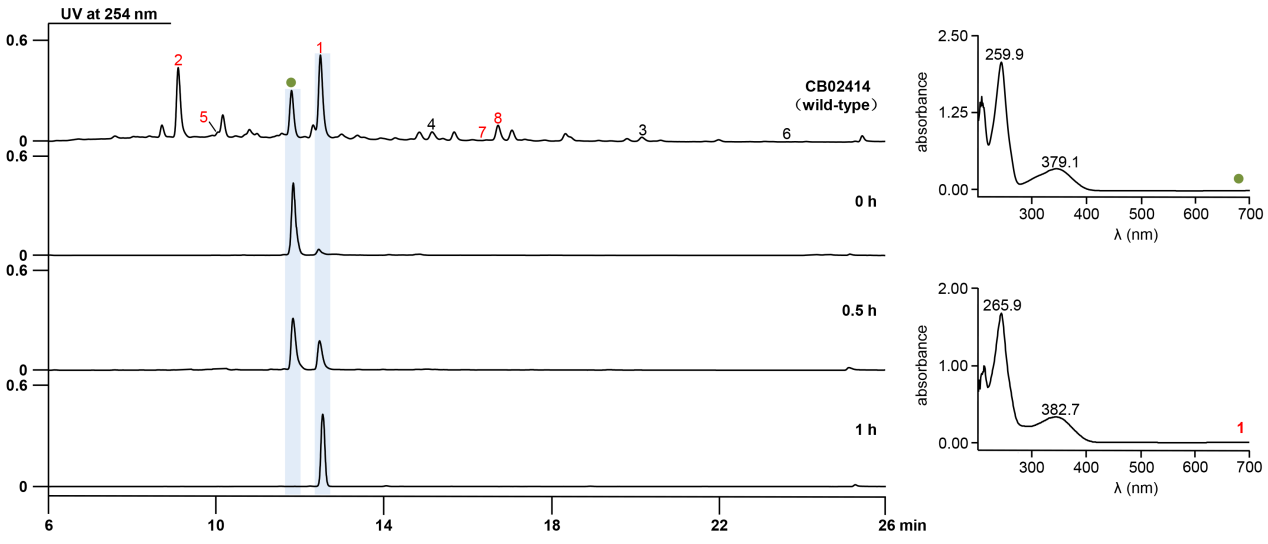
**

**Fig. S10.** HRESIMS spectrum of compounds **1**–**4**.

**Fig. S11.** ^1^H NMR spectrum of compound **1** in CDCl_3_ (400 MHz).

**Fig. S12.** ^13^C NMR spectrum of compound **1** in CDCl_3_ (101 MHz).

**Fig. S13.** DEPT 135 spectrum of compound **1** in CDCl_3_ (CDCl_3_).

**Fig. S14.** ^1^H-^1^H COSY and spectrum of compound **1** (CDCl_3_).

**Fig. S15.** ^1^H-^13^C HSQC spectrum of compound **1** (CDCl_3_).

**Fig. S16.** ^1^H-^13^C HMBC spectrum of compound **1** (CDCl_3_).

**Fig. S17.** ^1^H-^1^H NOESY spectrum of compound **1** (CDCl_3_).

**Fig. S18.** The energy lowest conformers and populations of rubiginone J (**1**) 2*S*, 3*S*, 4*R* and optimized coordinates.

A random conformational search was performed for rubiginone J (**1**) 2*S*, 3*S*, 4*R*, rubiginone J (**1**) 2*S*, 3*S*, 4*S*, rubiginone J (**1**) 2*R*, 3*S*, 4*R* and rubiginone J (**1**) 2*R*, 3*S*, 4*S* using the SYBYL 2.0 software package with MMFF94s molecular mechanics force field after the energy minimization [1], which yielded 25 conformers for rubiginone J (**1**) 2*S*, 3*S*, 4*R*, 20 for rubiginone J (**1**) 2*S*, 3*S*, 4*S*, 20 for rubiginone J (**1**) 2*R*, 3*S*, 4*R* and 24 for rubiginone J (**1**) 2*R*, 3*S*, 4*S* respectively, within a 10 kcal/mol energy window. The following geometry optimizations at the B3LYP/6-31 + G (d) level afforded 2 conformers for rubiginone J (**1**) 2*S*, 3*S*, 4*R* (Fig. S18), 4 for rubiginone J (**1**) 2*S*, 3*S*, 4*S* (Fig. S19), 3 for rubiginone J (**1**) 2*R*, 3*S*, 4*R* (Fig. S20) and 3 for rubiginone J (**1**) 2*R*, 3*S*, 4*S* (Fig. S21) respectively, within a 10 kcal/mol energy window. Subsequently, considering the lowest 100 excited states with the CPCM solvent model for methanol, the conformers were collected and submitted for ECD calculation at the B3LYP/6-31 + G (d) level through time-dependent density functional theory (TDDFT) calculation. According to the conformation distribution, the ECD spectra of all conformers were weighted by Boltzmann statistics. The comparison of the experimental ECD spectrum of rubiginone J (**1**) with the calculated ones (Fig. 5c) revealed that the Cotton effects (CEs) of rubiginone J (**1**) 2*S*, 3*S*, 4*S* were in good accordance with the experimental CEs of rubiginone J (**1**) in the region 200–450 nm.


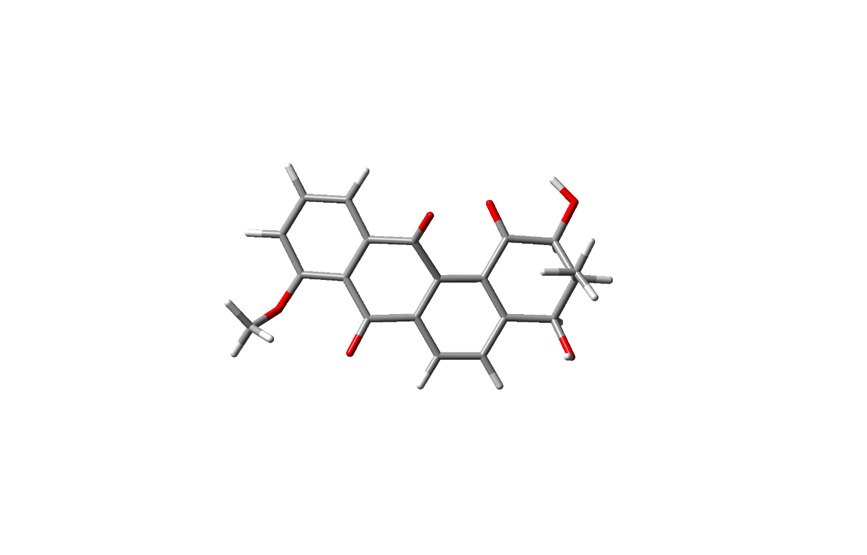

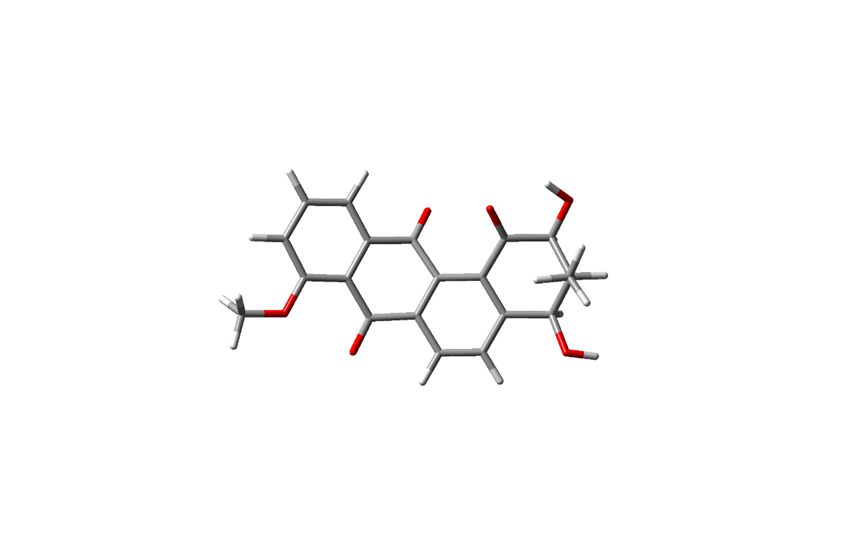


Rubiginone J (**1**) 2*S*, 3*S*, 4*R*_con1: 6.80% Rubiginone J (**1**) 2*S*, 3*S*, 4*R*_con2: 93.20%

Standard orientation of rubiginone J (**1**) 2*S*, 3*S*, 4*R*_con1:


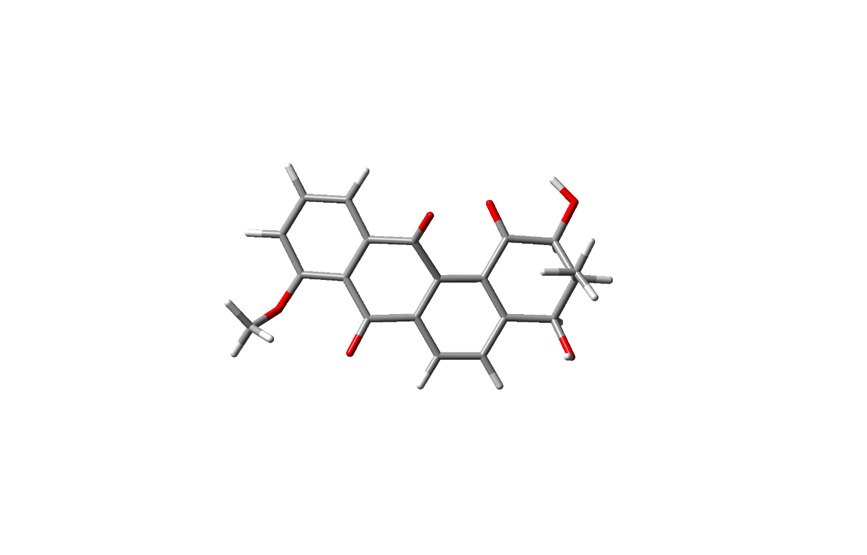


| C | 4.17081 | -2.39238 | -0.02611 |
| --- | --- | --- | --- |
| C | 4.87943 | -1.20062 | -0.15116 |
| C | 4.21701 | 0.03934 | -0.08144 |
| C | 2.80522 | 0.07215 | 0.09957 |
| C | 2.1153 | -1.16002 | 0.21529 |
| C | 2.78952 | -2.38065 | 0.16758 |
| C | 2.03703 | 1.33776 | 0.19768 |
| C | 0.53967 | 1.25446 | 0.08812 |
| C | -0.13565 | 0.02654 | 0.05396 |
| C | 0.63786 | -1.19245 | 0.42465 |
| C | -0.19152 | 2.45054 | -0.00269 |
| C | -1.57314 | 2.41814 | -0.115 |
| C | -2.26439 | 1.19685 | -0.19591 |
| C | -1.53528 | -0.00517 | -0.15522 |
| C | -3.79135 | 1.22863 | -0.25962 |
| C | -4.41418 | -0.07531 | -0.8031 |
| C | -3.74414 | -1.29172 | -0.13938 |
| C | -2.26779 | -1.25843 | -0.52479 |
| O | 0.09443 | -2.16357 | 0.94493 |
| O | 2.5638 | 2.44212 | 0.33234 |
| O | -1.77279 | -2.13186 | -1.22317 |
| O | -3.91223 | -1.17389 | 1.28277 |
| C | -5.94272 | -0.10209 | -0.68583 |
| O | -4.29138 | 1.58104 | 1.04196 |
| O | 4.87149 | 1.21342 | -0.19449 |
| C | 6.28815 | 1.21381 | -0.41889 |
| H | 4.70509 | -3.33651 | -0.08163 |
| H | 5.95129 | -1.2373 | -0.29949 |
| H | 2.22968 | -3.30342 | 0.26993 |
| H | 0.34199 | 3.39432 | 0.02874 |
| H | -2.13373 | 3.34812 | -0.15587 |
| H | -4.09931 | 2.05746 | -0.90412 |
| H | -4.15377 | -0.12634 | -1.87003 |
| H | -4.17864 | -2.22913 | -0.50363 |
| H | -3.57966 | -1.97421 | 1.7216 |
| H | -6.34389 | -0.98852 | -1.18985 |
| H | -6.37704 | 0.78474 | -1.16169 |
| H | -6.26783 | -0.12135 | 0.35768 |
| H | -4.16085 | 0.79962 | 1.61243 |
| H | 6.53781 | 0.70612 | -1.35663 |
| H | 6.56491 | 2.26607 | -0.48567 |
| H | 6.81837 | 0.74594 | 0.41735 |

Standard orientation of rubiginone J (**1**) 2*S*, 3*S*, 4*R*_con2:


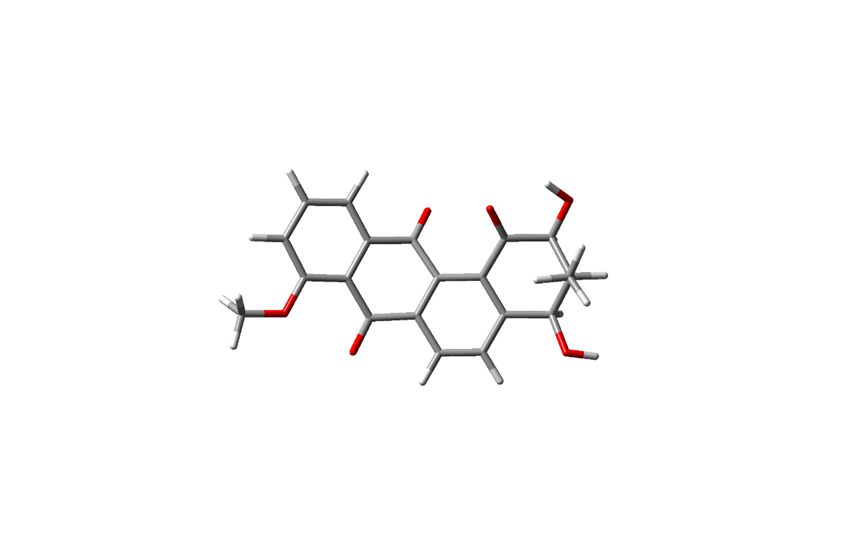


| C | -4.06895 | 2.40161 | 0.18439 |
| --- | --- | --- | --- |
| C | -4.76234 | 1.21425 | 0.40508 |
| C | -4.12384 | -0.03013 | 0.24776 |
| C | -2.75017 | -0.07068 | -0.12094 |
| C | -2.07218 | 1.15607 | -0.32272 |
| C | -2.7251 | 2.3818 | -0.18928 |
| C | -2.00923 | -1.33778 | -0.33836 |
| C | -0.5074 | -1.26444 | -0.36713 |
| C | 0.17515 | -0.04091 | -0.391 |
| C | -0.63306 | 1.17168 | -0.71637 |
| C | 0.22326 | -2.46321 | -0.34332 |
| C | 1.61011 | -2.44277 | -0.34694 |
| C | 2.31192 | -1.22619 | -0.31319 |
| C | 1.58793 | -0.01577 | -0.29063 |
| C | 3.83253 | -1.23119 | -0.36933 |
| C | 4.47297 | 0.00473 | 0.29619 |
| C | 3.81035 | 1.25454 | -0.33852 |
| C | 2.33302 | 1.23796 | 0.01473 |
| O | -0.15925 | 2.11044 | -1.34922 |
| O | -2.5575 | -2.43247 | -0.46203 |
| O | 1.85139 | 2.17721 | 0.63974 |
| O | 4.41354 | 2.45826 | 0.08818 |
| C | 4.39167 | -0.01193 | 1.82779 |
| O | 4.30443 | -2.45904 | 0.18673 |
| O | -4.76148 | -1.20156 | 0.45158 |
| C | -6.13279 | -1.19454 | 0.87236 |
| H | -4.58553 | 3.34898 | 0.3091 |
| H | -5.80435 | 1.2585 | 0.69591 |
| H | -2.17731 | 3.30109 | -0.36411 |
| H | -0.31603 | -3.40436 | -0.3355 |
| H | 2.16987 | -3.37093 | -0.35051 |
| H | 4.09812 | -1.20709 | -1.43917 |
| H | 5.53064 | 0.03021 | 0.00313 |
| H | 3.90515 | 1.18047 | -1.4332 |
| H | 3.71903 | 2.9626 | 0.55893 |
| H | 4.9701 | -0.84967 | 2.22703 |
| H | 4.80511 | 0.91523 | 2.23685 |
| H | 3.36236 | -0.11619 | 2.19163 |
| H | 5.23223 | -2.57587 | -0.07516 |
| H | -6.24598 | -0.68512 | 1.8353 |
| H | -6.40302 | -2.24527 | 0.97835 |
| H | -6.77242 | -0.72398 | 0.11822 |

**Fig. S19**. The energy lowest conformers and populations of rubiginone J (**1**) 2*S*, 3*S*, 4*S* and optimized coordinates.


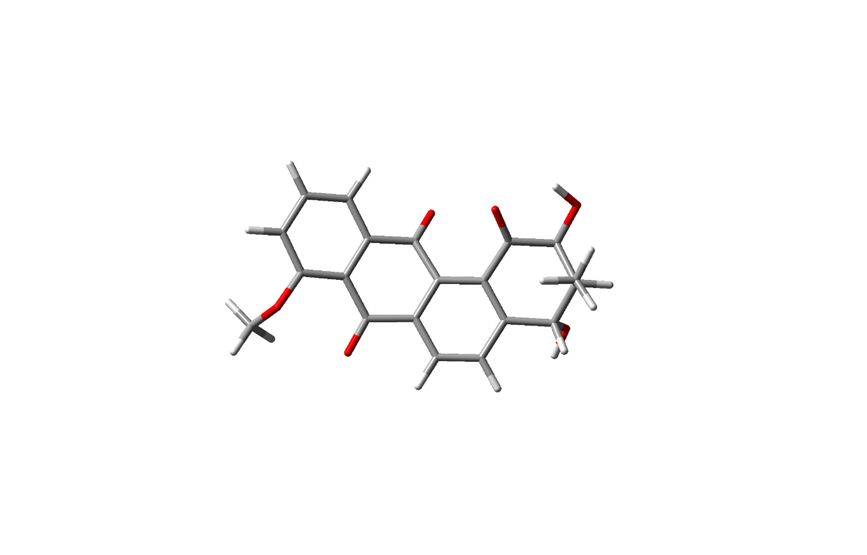

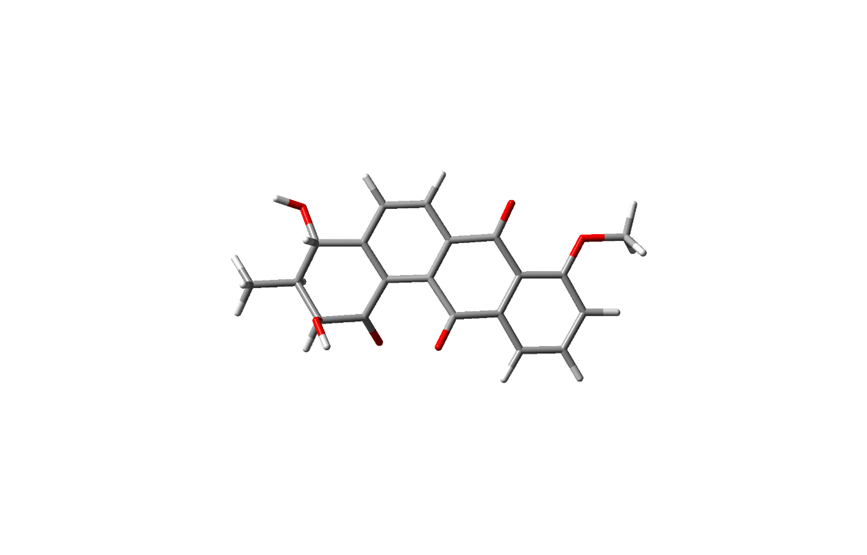


Rubiginone J (**1**) 2*S*, 3*S*, 4*S*_con1: 0.36% Rubiginone J (**1**) 2*S*, 3*S*, 4*S*_con2: 0.73%


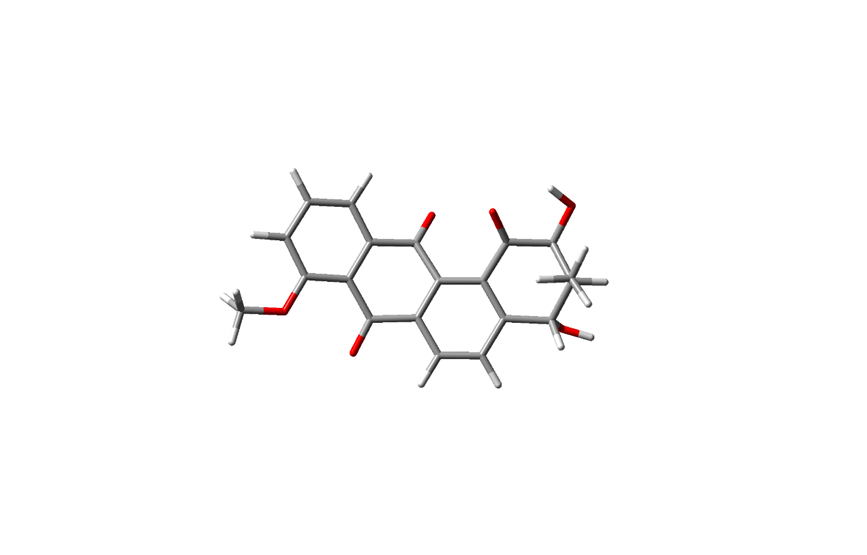

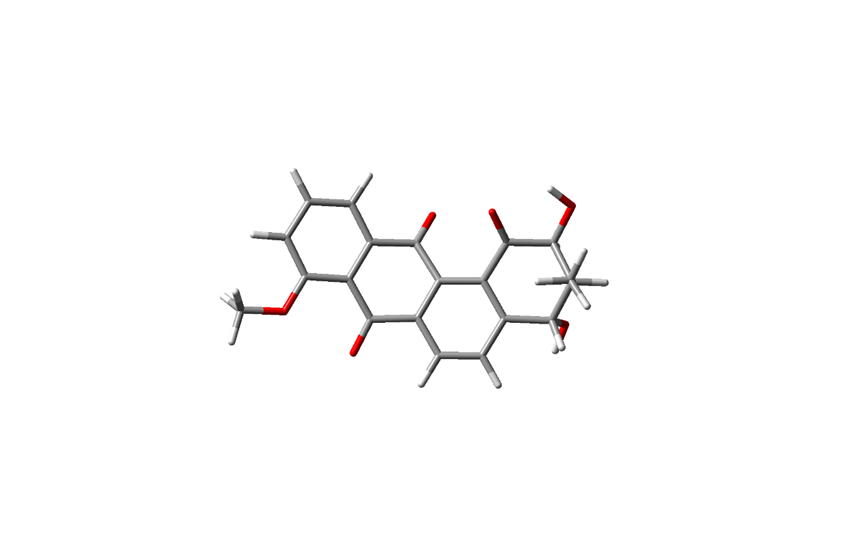


Rubiginone J (**1**) 2*S*, 3*S*, 4*S*_con3: 43.00% Rubiginone J (**1**) 2*S*, 3*S*, 4*S*_con4: 55.91%

Standard orientation of rubiginone J (**1**) 2*S*, 3*S*, 4*S*_con1:


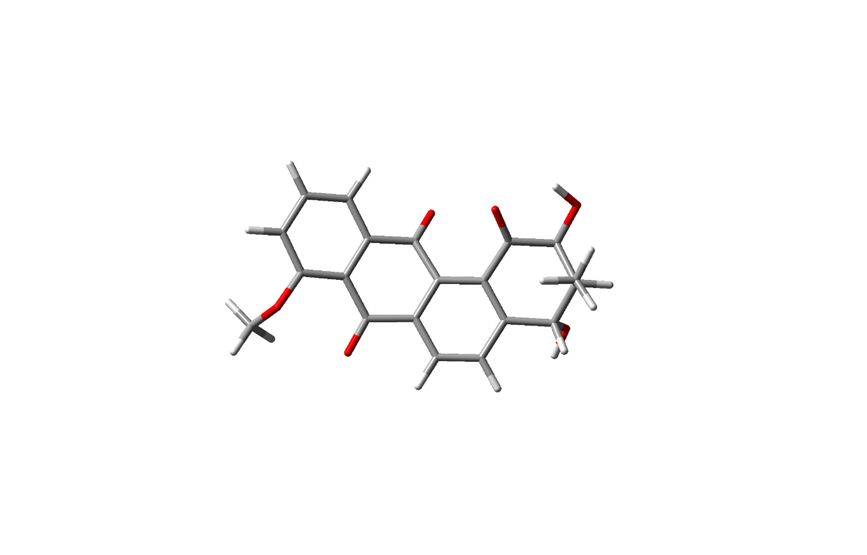


| C | -4.00845 | 2.56376 | 0.1013 |
| --- | --- | --- | --- |
| C | -4.73194 | 1.42199 | 0.44119 |
| C | -4.13289 | 0.15584 | 0.40537 |
| C | -2.77255 | 0.02681 | 0.04454 |
| C | -2.04847 | 1.20218 | -0.27314 |
| C | -2.66591 | 2.4547 | -0.26534 |
| C | -2.08005 | -1.28811 | -0.03633 |
| C | -0.57867 | -1.27734 | -0.0568 |
| C | 0.1488 | -0.08718 | -0.21423 |
| C | -0.60864 | 1.12573 | -0.65243 |
| C | 0.10038 | -2.4937 | 0.11081 |
| C | 1.48791 | -2.52459 | 0.10542 |
| C | 2.23469 | -1.34266 | -0.02007 |
| C | 1.5607 | -0.10924 | -0.12492 |
| C | 3.75495 | -1.43282 | -0.11176 |
| C | 4.47321 | -0.16877 | 0.37492 |
| C | 3.83596 | 1.06071 | -0.32549 |
| C | 2.36926 | 1.13295 | 0.05968 |
| O | -0.08925 | 1.98743 | -1.35457 |
| O | -2.68002 | -2.36 | -0.06432 |
| O | 1.93758 | 2.13314 | 0.62277 |
| O | 4.49884 | 2.26587 | 0.00265 |
| C | 4.47348 | -0.05832 | 1.9058 |
| O | 4.13229 | -1.62073 | -1.49181 |
| O | -4.87601 | -0.91954 | 0.8134 |
| C | -5.70664 | -1.5238 | -0.19591 |
| H | -4.48766 | 3.53787 | 0.13287 |
| H | -5.77156 | 1.49215 | 0.74737 |
| H | -2.08623 | 3.33315 | -0.52773 |
| H | -0.47553 | -3.4043 | 0.23577 |
| H | 2.00521 | -3.47371 | 0.2199 |
| H | 4.09868 | -2.2948 | 0.47305 |
| H | 5.51117 | -0.23368 | 0.02822 |
| H | 3.89731 | 0.90463 | -1.41101 |
| H | 3.84084 | 2.83001 | 0.45673 |
| H | 3.4614 | -0.07437 | 2.32835 |
| H | 4.95608 | 0.87176 | 2.21977 |
| H | 5.02989 | -0.89425 | 2.34369 |
| H | 3.75724 | -2.45737 | -1.81359 |
| H | -6.2506 | -2.32632 | 0.3047 |
| H | -6.41573 | -0.79176 | -0.5998 |
| H | -5.08882 | -1.93721 | -0.99884 |

Standard orientation of rubiginone J (**1**) 2*S*, 3*S*, 4*S*_con2:


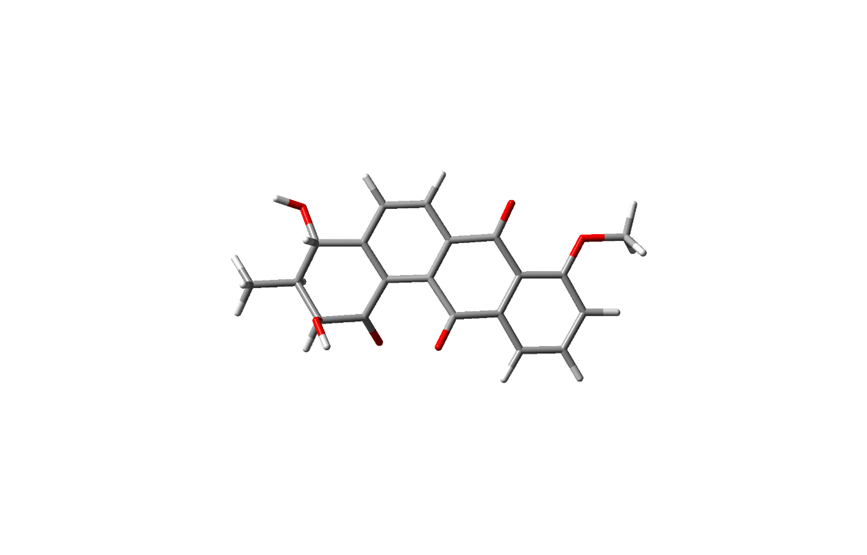


| C | 4.20831 | -2.34637 | -0.24506 |
| --- | --- | --- | --- |
| C | 4.89251 | -1.13529 | -0.30401 |
| C | 4.21225 | 0.08332 | -0.12207 |
| C | 2.80701 | 0.07581 | 0.10623 |
| C | 2.14153 | -1.17423 | 0.15054 |
| C | 2.83395 | -2.37514 | -0.00891 |
| C | 2.02168 | 1.31527 | 0.32848 |
| C | 0.52397 | 1.21251 | 0.25546 |
| C | -0.13251 | -0.02103 | 0.14635 |
| C | 0.67194 | -1.24945 | 0.40159 |
| C | -0.23154 | 2.3957 | 0.2806 |
| C | -1.61554 | 2.34737 | 0.20542 |
| C | -2.28912 | 1.12292 | 0.05046 |
| C | -1.53682 | -0.06417 | -0.02264 |
| C | -3.81277 | 1.12034 | 0.02568 |
| C | -4.43882 | -0.15928 | -0.56683 |
| C | -3.7097 | -1.39997 | -0.02319 |
| C | -2.25442 | -1.30169 | -0.46398 |
| O | 0.16216 | -2.2662 | 0.86579 |
| O | 2.53485 | 2.41461 | 0.53743 |
| O | -1.76367 | -2.10769 | -1.24302 |
| O | -3.81795 | -1.40598 | 1.40427 |
| C | -5.95282 | -0.23395 | -0.33061 |
| O | -4.24773 | 2.2844 | -0.69173 |
| O | 4.84273 | 1.27506 | -0.16992 |
| C | 6.25112 | 1.31894 | -0.43745 |
| H | 4.75631 | -3.27351 | -0.38654 |
| H | 5.95948 | -1.14082 | -0.48803 |
| H | 2.29314 | -3.31344 | 0.04224 |
| H | 0.28506 | 3.34474 | 0.37519 |
| H | -2.19472 | 3.26345 | 0.24435 |
| H | -4.1353 | 1.20988 | 1.07291 |
| H | -4.25505 | -0.13564 | -1.65032 |
| H | -4.14055 | -2.31264 | -0.45112 |
| H | -3.47197 | -2.24823 | 1.74197 |
| H | -6.46354 | 0.6185 | -0.79123 |
| H | -6.18893 | -0.24298 | 0.739 |
| H | -6.36464 | -1.14475 | -0.77958 |
| H | -5.10667 | 2.56623 | -0.34065 |
| H | 6.81594 | 0.80311 | 0.34634 |
| H | 6.47952 | 0.88429 | -1.41646 |
| H | 6.50751 | 2.37847 | -0.43704 |

Standard orientation of rubiginone J (**1**) 2*S*, 3*S*, 4*S*_con3:


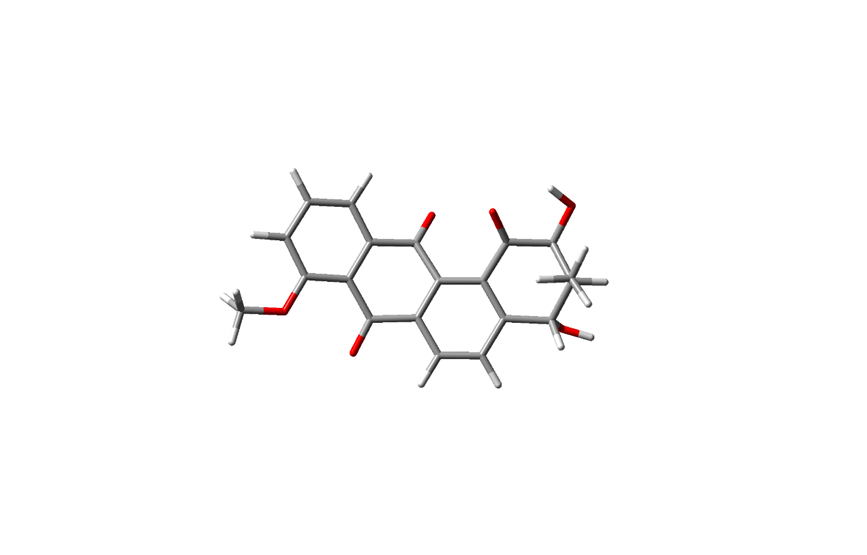


| C | -4.03142 | 2.45515 | -0.03149 |
| --- | --- | --- | --- |
| C | -4.76296 | 1.2978 | 0.2211 |
| C | -4.1453 | 0.03381 | 0.17695 |
| C | -2.75327 | -0.05694 | -0.10684 |
| C | -2.03851 | 1.14172 | -0.35033 |
| C | -2.67027 | 2.38537 | -0.32884 |
| C | -2.03148 | -1.34972 | -0.18692 |
| C | -0.52668 | -1.30882 | -0.1794 |
| C | 0.18394 | -0.10286 | -0.25726 |
| C | -0.58218 | 1.1086 | -0.67251 |
| C | 0.1716 | -2.52148 | -0.06432 |
| C | 1.55817 | -2.52844 | -0.03294 |
| C | 2.28531 | -1.32797 | -0.05476 |
| C | 1.59427 | -0.10448 | -0.12444 |
| C | 3.8064 | -1.39605 | -0.06363 |
| C | 4.4879 | -0.11822 | 0.45741 |
| C | 3.84831 | 1.10751 | -0.24342 |
| C | 2.37481 | 1.14481 | 0.11949 |
| O | -0.05665 | 2.00914 | -1.32034 |
| O | -2.59612 | -2.44241 | -0.22481 |
| O | 1.91798 | 2.11813 | 0.70879 |
| O | 4.48252 | 2.32135 | 0.10953 |
| C | 4.44492 | -0.02913 | 1.99004 |
| O | 4.15448 | -1.66335 | -1.43892 |
| O | -4.82248 | -1.10922 | 0.40884 |
| C | -6.21764 | -1.0494 | 0.73739 |
| H | -4.53184 | 3.41849 | 0.00619 |
| H | -5.81833 | 1.38014 | 0.44849 |
| H | -2.09352 | 3.28095 | -0.5308 |
| H | -0.39104 | -3.44715 | -0.01162 |
| H | 2.09356 | -3.47173 | 0.02938 |
| H | 4.12342 | -2.24677 | 0.55253 |
| H | 5.53967 | -0.1519 | 0.14534 |
| H | 3.9341 | 0.96438 | -1.32912 |
| H | 3.81075 | 2.86047 | 0.5737 |
| H | 3.42222 | -0.07505 | 2.38351 |
| H | 4.89756 | 0.90645 | 2.33173 |
| H | 5.00784 | -0.85925 | 2.43094 |
| H | 5.08716 | -1.93614 | -1.46877 |
| H | -6.38021 | -0.48196 | 1.65986 |
| H | -6.79543 | -0.61119 | -0.08324 |
| H | -6.51927 | -2.08638 | 0.88568 |

Standard orientation of rubiginone J (**1**) 2*S*, 3*S*, 4*S*_con4:


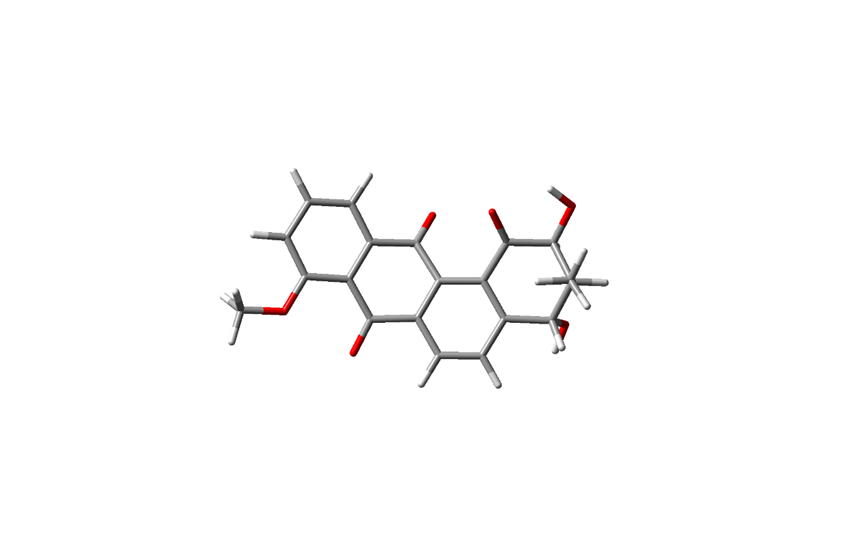


| C | -4.02432 | 2.45771 | -0.03348 |
| --- | --- | --- | --- |
| C | -4.75641 | 1.30153 | 0.22282 |
| C | -4.14041 | 0.03673 | 0.17853 |
| C | -2.74926 | -0.05612 | -0.10892 |
| C | -2.03395 | 1.14149 | -0.35657 |
| C | -2.66421 | 2.38593 | -0.33502 |
| C | -2.02902 | -1.35002 | -0.18694 |
| C | -0.5244 | -1.31037 | -0.17945 |
| C | 0.18769 | -0.10527 | -0.26773 |
| C | -0.57896 | 1.1062 | -0.68386 |
| C | 0.17262 | -2.52101 | -0.045 |
| C | 1.56011 | -2.52914 | -0.01283 |
| C | 2.2901 | -1.33083 | -0.06239 |
| C | 1.597 | -0.10636 | -0.13604 |
| C | 3.81417 | -1.39126 | -0.10215 |
| C | 4.49124 | -0.13506 | 0.4586 |
| C | 3.85451 | 1.10995 | -0.2137 |
| C | 2.37472 | 1.14083 | 0.1252 |
| O | -0.05442 | 2.00413 | -1.3359 |
| O | -2.59483 | -2.44221 | -0.22222 |
| O | 1.90692 | 2.11058 | 0.7126 |
| O | 4.48332 | 2.31252 | 0.18419 |
| C | 4.43733 | -0.08662 | 1.99183 |
| O | 4.24575 | -1.52 | -1.47336 |
| O | -4.81831 | -1.10514 | 0.41382 |
| C | -6.2124 | -1.04331 | 0.74667 |
| H | -4.52351 | 3.42168 | 0.00456 |
| H | -5.81097 | 1.38541 | 0.45336 |
| H | -2.08704 | 3.28051 | -0.54013 |
| H | -0.39055 | -3.44518 | 0.02573 |
| H | 2.09001 | -3.47427 | 0.0751 |
| H | 4.15261 | -2.26903 | 0.46192 |
| H | 5.54143 | -0.1677 | 0.14585 |
| H | 3.95452 | 0.99904 | -1.30194 |
| H | 3.79898 | 2.84729 | 0.63495 |
| H | 4.8903 | 0.83875 | 2.35943 |
| H | 3.41182 | -0.13884 | 2.37728 |
| H | 4.99492 | -0.92933 | 2.41505 |
| H | 3.9117 | -2.35755 | -1.8353 |
| H | -6.51454 | -2.07974 | 0.89765 |
| H | -6.79232 | -0.60592 | -0.07289 |
| H | -6.3715 | -0.47414 | 1.66867 |

**Fig. S20**. The energy lowest conformers and populations of rubiginone J (**1**) 2*R*, 3*S*, 4*R* and optimized coordinates.


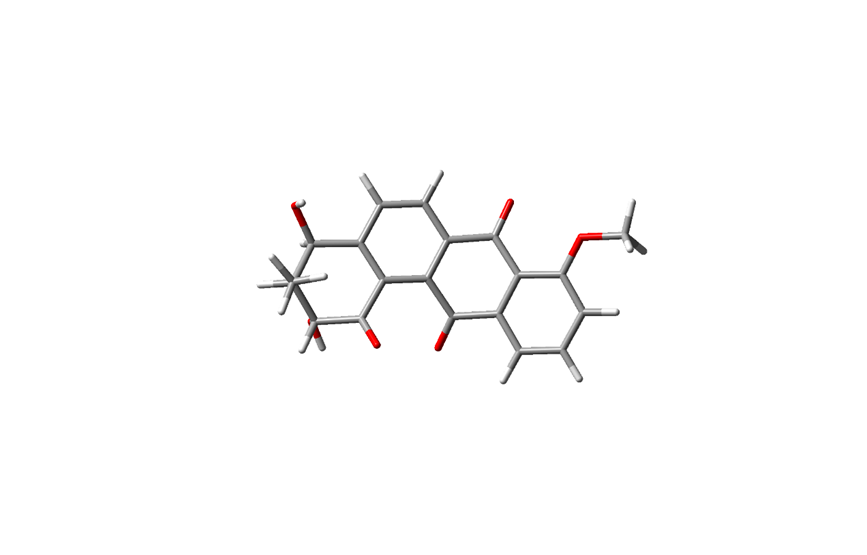

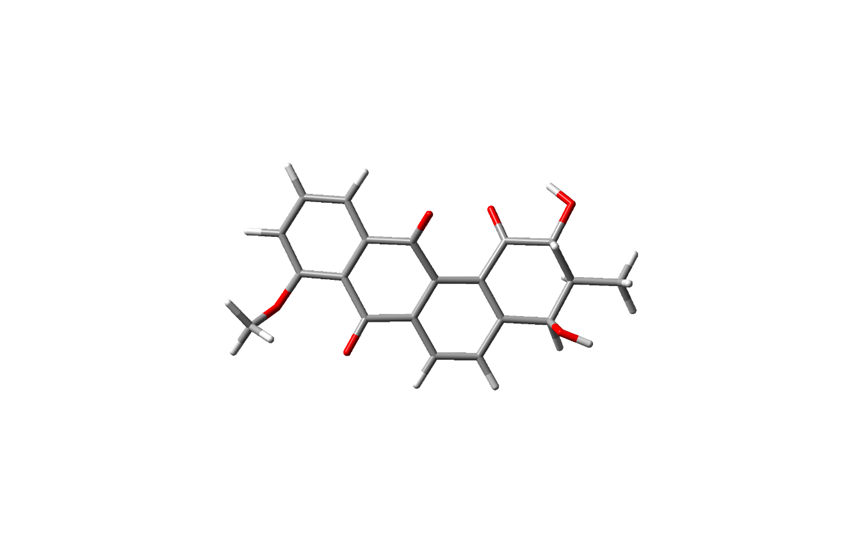


Rubiginone J (**1**) 2*R*, 3*S*, 4*R*_con1: 15.11% Rubiginone J (**1**) 2*R*, 3*S*, 4*R*_con2: 25.57%


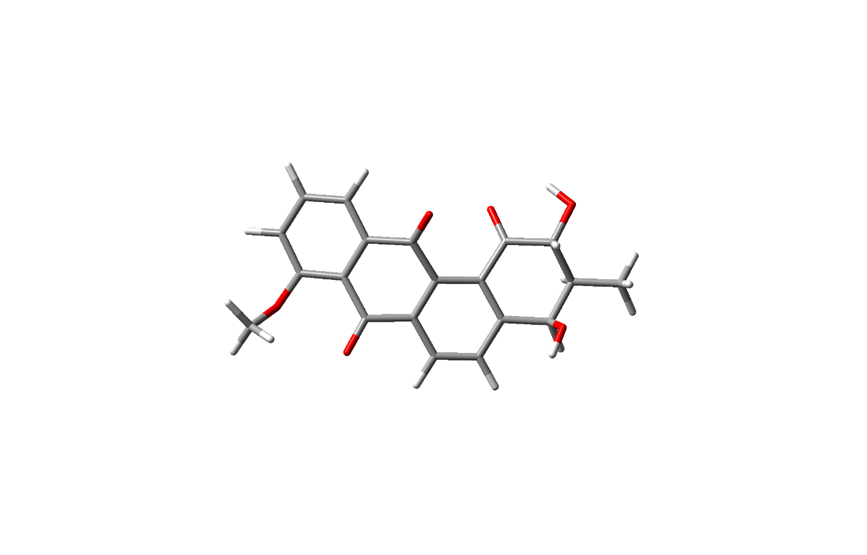


Rubiginone J (**1**) 2*R*, 3*S*, 4*R*_con3: 59.32%

Standard orientation of rubiginone J (**1**) 2*R*, 3*S*, 4*R*_con1:


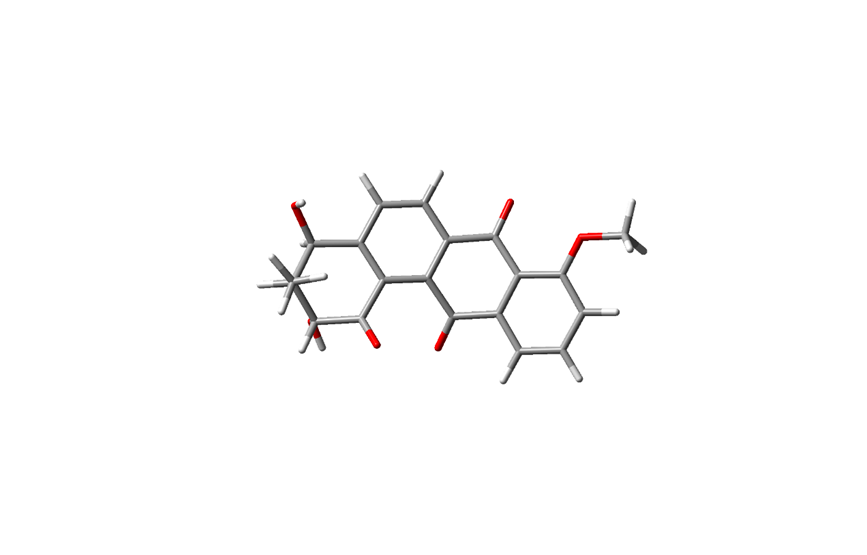


| C | 4.13236 | -2.33126 | 0.29757 |
| --- | --- | --- | --- |
| C | 4.80393 | -1.11589 | 0.4018 |
| C | 4.12606 | 0.09924 | 0.19056 |
| C | 2.73588 | 0.08271 | -0.11418 |
| C | 2.08175 | -1.17072 | -0.19957 |
| C | 2.77271 | -2.36836 | -0.01182 |
| C | 1.95412 | 1.31601 | -0.37883 |
| C | 0.45544 | 1.20581 | -0.35024 |
| C | -0.19752 | -0.03242 | -0.27365 |
| C | 0.62728 | -1.2498 | -0.52661 |
| C | -0.30362 | 2.38628 | -0.38079 |
| C | -1.68901 | 2.33158 | -0.33947 |
| C | -2.36042 | 1.10369 | -0.21097 |
| C | -1.60533 | -0.08427 | -0.13741 |
| C | -3.88952 | 1.08738 | -0.23181 |
| C | -4.51548 | -0.17024 | 0.41261 |
| C | -3.79065 | -1.41345 | -0.13083 |
| C | -2.3224 | -1.33129 | 0.27815 |
| O | 0.15041 | -2.25374 | -1.04854 |
| O | 2.46916 | 2.41433 | -0.58729 |
| O | -1.81729 | -2.16964 | 1.01282 |
| O | -3.9231 | -1.41324 | -1.55687 |
| C | -4.51177 | -0.14182 | 1.95002 |
| O | -4.45466 | 2.29702 | 0.27473 |
| O | 4.74251 | 1.29593 | 0.28289 |
| C | 6.13335 | 1.34881 | 0.62867 |
| H | 4.67879 | -3.25547 | 0.46242 |
| H | 5.85924 | -1.11592 | 0.64419 |
| H | 2.24235 | -3.31021 | -0.09725 |
| H | 0.21136 | 3.3383 | -0.45199 |
| H | -2.27073 | 3.24612 | -0.38852 |
| H | -4.17686 | 1.09052 | -1.28878 |
| H | -5.5561 | -0.23188 | 0.07096 |
| H | -4.2199 | -2.32551 | 0.29894 |
| H | -3.57322 | -2.24982 | -1.90536 |
| H | -3.51271 | 0.03127 | 2.36832 |
| H | -4.87869 | -1.09384 | 2.34896 |
| H | -5.1768 | 0.64293 | 2.3238 |
| H | -4.22226 | 2.39436 | 1.21346 |
| H | 6.31068 | 0.90806 | 1.61553 |
| H | 6.74481 | 0.84361 | -0.12647 |
| H | 6.38066 | 2.41026 | 0.6506 |

Standard orientation of rubiginone J (**1**) 2*R*, 3*S*, 4*R*_con2:


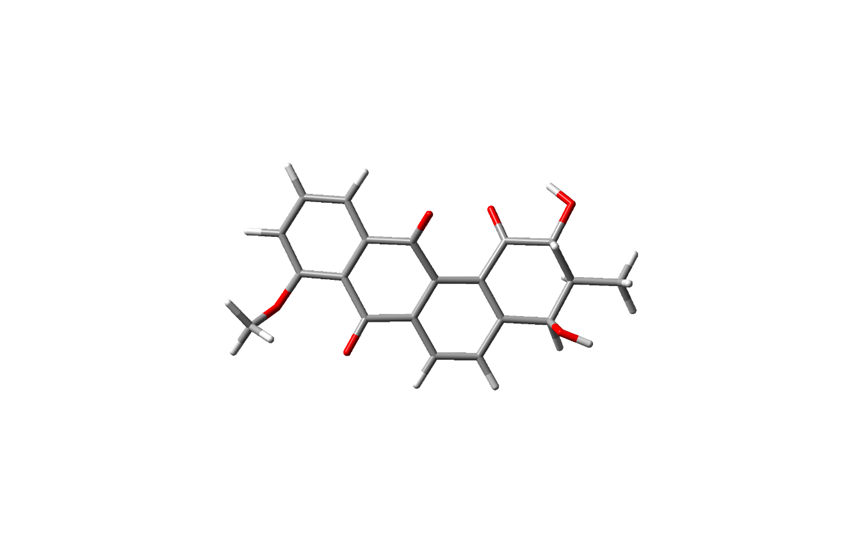


| C | -4.10671 | 2.54047 | -0.05509 |
| --- | --- | --- | --- |
| C | -4.8286 | 1.39357 | -0.38078 |
| C | -4.21727 | 0.13277 | -0.365 |
| C | -2.84771 | 0.01494 | -0.03817 |
| C | -2.12498 | 1.19578 | 0.26109 |
| C | -2.75356 | 2.44265 | 0.27416 |
| C | -2.14385 | -1.29471 | 0.03184 |
| C | -0.64256 | -1.27278 | 0.00723 |
| C | 0.07831 | -0.07606 | 0.1315 |
| C | -0.67305 | 1.1311 | 0.59345 |
| C | 0.04227 | -2.48721 | -0.16071 |
| C | 1.42823 | -2.50603 | -0.19813 |
| C | 2.1659 | -1.31344 | -0.1282 |
| C | 1.48802 | -0.08794 | -0.00538 |
| C | 3.68469 | -1.39501 | -0.12694 |
| C | 4.37714 | -0.09936 | -0.58996 |
| C | 3.7572 | 1.11214 | 0.14655 |
| C | 2.27912 | 1.16101 | -0.20444 |
| O | -0.1374 | 1.99809 | 1.27693 |
| O | -2.73539 | -2.37008 | 0.08794 |
| O | 1.82447 | 2.15541 | -0.75961 |
| O | 4.38145 | 2.33045 | -0.21358 |
| C | 5.89963 | -0.15952 | -0.42254 |
| O | 4.02922 | -1.74489 | 1.23031 |
| O | -4.95719 | -0.94924 | -0.76089 |
| C | -5.78311 | -1.54816 | 0.25519 |
| H | -4.59526 | 3.51033 | -0.07049 |
| H | -5.87594 | 1.45626 | -0.66118 |
| H | -2.17473 | 3.32583 | 0.52212 |
| H | -0.52954 | -3.40469 | -0.24867 |
| H | 1.95489 | -3.45052 | -0.30155 |
| H | 3.99297 | -2.21212 | -0.79138 |
| H | 4.1538 | 0.01532 | -1.66089 |
| H | 3.86132 | 0.96006 | 1.2307 |
| H | 3.68308 | 2.88376 | -0.61954 |
| H | 6.36591 | 0.72481 | -0.86457 |
| H | 6.30711 | -1.04454 | -0.92685 |
| H | 6.18978 | -0.19716 | 0.63418 |
| H | 4.93131 | -2.10498 | 1.23721 |
| H | -6.49084 | -0.81494 | 0.65927 |
| H | -6.32906 | -2.35353 | -0.23862 |
| H | -5.16095 | -1.95699 | 1.05728 |

Standard orientation of rubiginone J (**1**) 2*R*, 3*S*, 4*R*_con3:


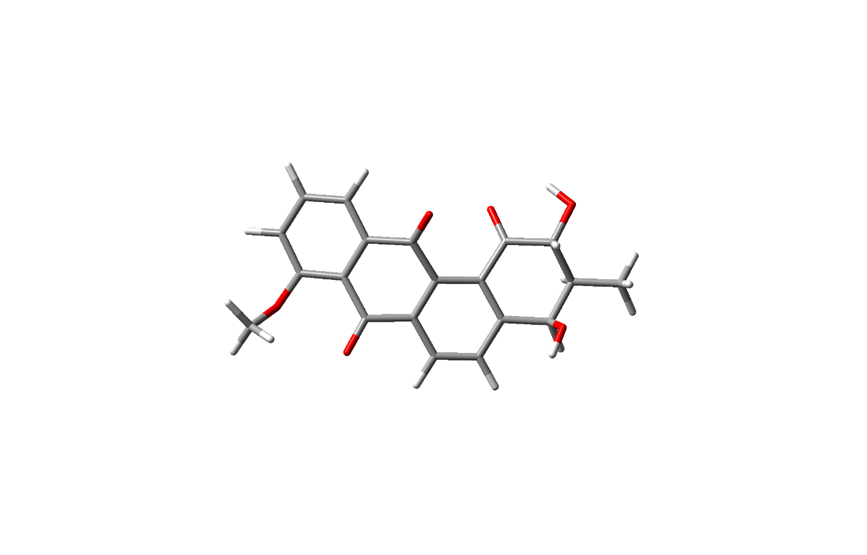


| C | -4.09628 | 2.54641 | -0.0462 |
| --- | --- | --- | --- |
| C | -4.82019 | 1.40292 | -0.3795 |
| C | -4.21229 | 0.14045 | -0.36806 |
| C | -2.84378 | 0.01751 | -0.03843 |
| C | -2.11921 | 1.1949 | 0.27003 |
| C | -2.74461 | 2.44338 | 0.28731 |
| C | -2.14277 | -1.29412 | 0.02222 |
| C | -0.64169 | -1.27492 | -0.00064 |
| C | 0.08233 | -0.08119 | 0.1422 |
| C | -0.66938 | 1.12426 | 0.61015 |
| C | 0.03994 | -2.48647 | -0.19451 |
| C | 1.42699 | -2.50893 | -0.22858 |
| C | 2.16896 | -1.32187 | -0.12006 |
| C | 1.49134 | -0.09432 | 0.00988 |
| C | 3.6897 | -1.39877 | -0.07091 |
| C | 4.38058 | -0.13173 | -0.59207 |
| C | 3.76986 | 1.10625 | 0.11039 |
| C | 2.28224 | 1.15215 | -0.20372 |
| O | -0.13602 | 1.98348 | 1.30484 |
| O | -2.73666 | -2.36874 | 0.06803 |
| O | 1.8145 | 2.14814 | -0.74624 |
| O | 4.38837 | 2.30827 | -0.30969 |
| C | 5.9044 | -0.19194 | -0.44252 |
| O | 4.09799 | -1.58559 | 1.2998 |
| O | -4.95477 | -0.93741 | -0.77058 |
| C | -5.77731 | -1.54416 | 0.24367 |
| H | -4.58228 | 3.5176 | -0.05844 |
| H | -5.86662 | 1.46956 | -0.66239 |
| H | -2.1644 | 3.3237 | 0.54222 |
| H | -0.53395 | -3.39992 | -0.3076 |
| H | 1.94709 | -3.45421 | -0.36128 |
| H | 4.02548 | -2.25767 | -0.6654 |
| H | 4.13879 | -0.05569 | -1.66179 |
| H | 3.89603 | 0.99447 | 1.19715 |
| H | 3.67394 | 2.86567 | -0.68073 |
| H | 6.20012 | -0.22179 | 0.61081 |
| H | 6.36628 | 0.68264 | -0.90875 |
| H | 6.29833 | -1.0893 | -0.93458 |
| H | 3.76228 | -2.44027 | 1.61621 |
| H | -5.15277 | -1.95847 | 1.04102 |
| H | -6.48418 | -0.81406 | 0.65487 |
| H | -6.3243 | -2.34623 | -0.25435 |

**Fig. S21.** The energy lowest conformers and populations of rubiginone J (**1**) 2*R*, 3*S*, 4*S* and optimized coordinates.


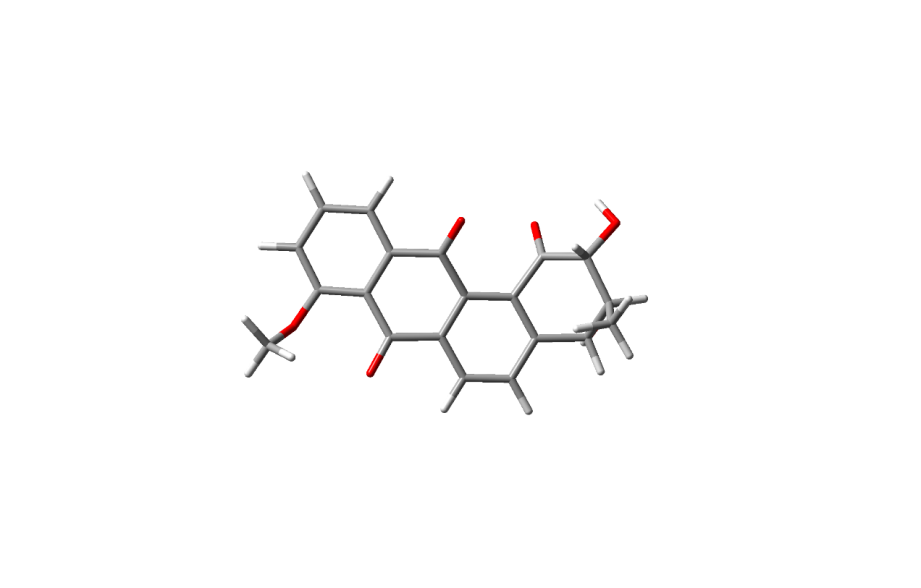

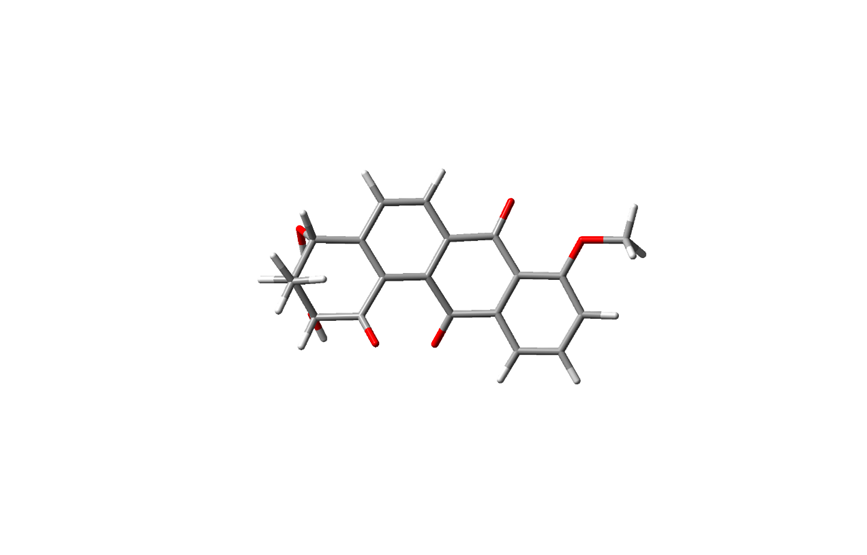


Rubiginone J (**1**) 2*R*, 3*S*, 4*S*_con1: 0.03% Rubiginone J (**1**) 2*R*, 3*S*, 4*S*_con2: 3.48%


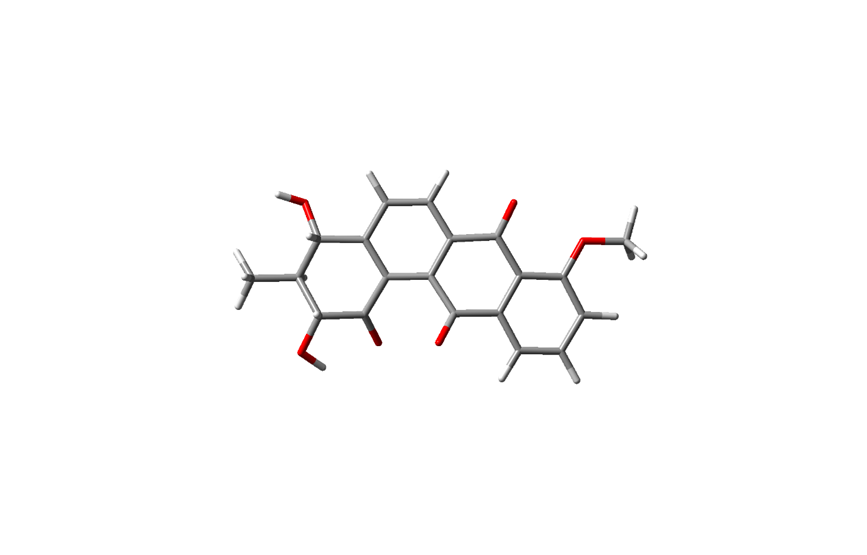


Rubiginone J (**1**) 2*R*, 3*S*, 4*S*_con3: 96.49%

Standard orientation of rubiginone J (**1**) 2*R*, 3*S*, 4*S*_con1:


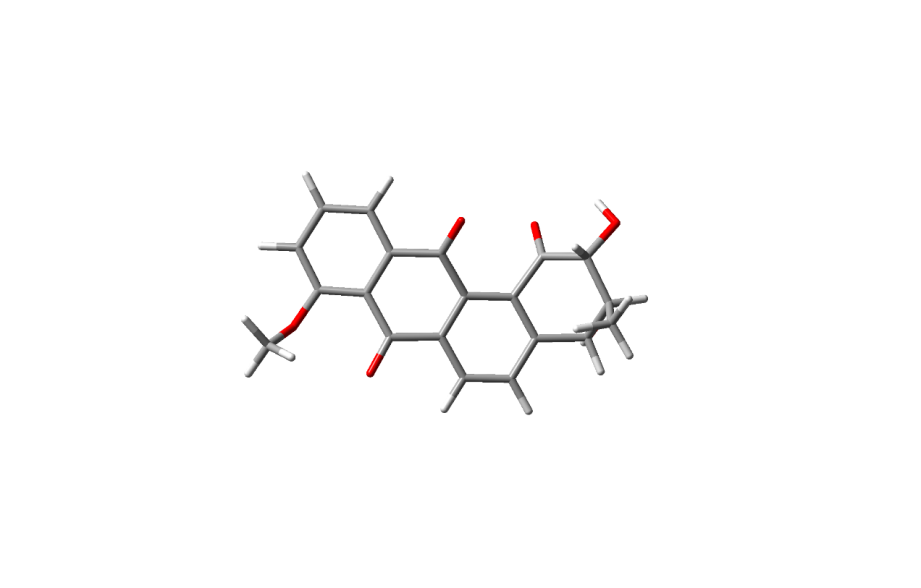


| C | -4.05968 | 2.54515 | -0.12239 |
| --- | --- | --- | --- |
| C | -4.79539 | 1.37681 | -0.31093 |
| C | -4.1756 | 0.12127 | -0.25374 |
| C | -2.78423 | 0.02721 | -0.02325 |
| C | -2.04921 | 1.22885 | 0.1374 |
| C | -2.68514 | 2.47218 | 0.10735 |
| C | -2.07409 | -1.27822 | 0.07578 |
| C | -0.57758 | -1.2614 | -0.02943 |
| C | 0.14684 | -0.05542 | -0.0008 |
| C | -0.5746 | 1.20073 | 0.35298 |
| C | 0.10027 | -2.48197 | -0.15114 |
| C | 1.48784 | -2.50907 | -0.25496 |
| C | 2.21826 | -1.31502 | -0.26811 |
| C | 1.54438 | -0.08531 | -0.16364 |
| C | 3.72737 | -1.28673 | -0.44002 |
| C | 4.38832 | -0.29709 | 0.53573 |
| C | 3.70574 | 1.10627 | 0.49044 |
| C | 2.41719 | 1.1168 | -0.32653 |
| O | 0.02005 | 2.16155 | 0.83503 |
| O | -2.66113 | -2.34713 | 0.22698 |
| O | 2.18534 | 2.0302 | -1.10675 |
| O | 4.59578 | 2.09836 | 0.00528 |
| C | 4.44068 | -0.86532 | 1.96146 |
| O | 4.06945 | -0.84204 | -1.76631 |
| O | -4.93916 | -0.98448 | -0.51684 |
| C | -5.67293 | -1.51816 | 0.60117 |
| H | -4.55474 | 3.51074 | -0.16805 |
| H | -5.86142 | 1.41719 | -0.51446 |
| H | -2.09437 | 3.37108 | 0.24595 |
| H | -0.47402 | -3.40215 | -0.15452 |
| H | 2.00854 | -3.4595 | -0.33553 |
| H | 4.13392 | -2.29132 | -0.27144 |
| H | 5.41179 | -0.14314 | 0.17734 |
| H | 3.39418 | 1.37207 | 1.51518 |
| H | 4.06872 | 2.68183 | -0.57669 |
| H | 3.43685 | -1.0367 | 2.36828 |
| H | 4.98182 | -1.81781 | 1.98227 |
| H | 4.95794 | -0.16755 | 2.62901 |
| H | 3.64376 | -1.42617 | -2.41635 |
| H | -6.34758 | -0.76048 | 1.01666 |
| H | -4.98392 | -1.8763 | 1.37219 |
| H | -6.25472 | -2.35275 | 0.20685 |

Standard orientation of rubiginone J (**1**) 2*R*, 3*S*, 4*S*_con2:


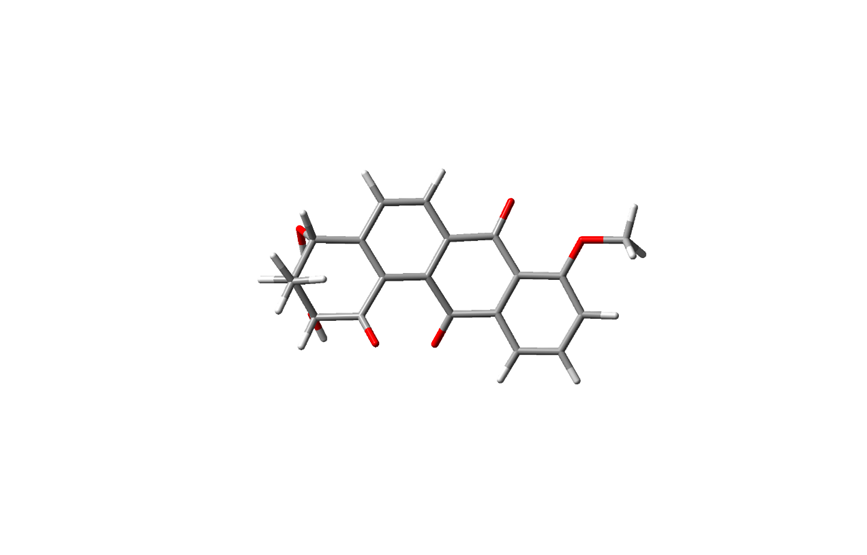


| C | 4.09664 | -2.39152 | 0.03909 |
| --- | --- | --- | --- |
| C | 4.80241 | -1.20125 | 0.19098 |
| C | 4.1423 | 0.03967 | 0.11607 |
| C | 2.73502 | 0.07499 | -0.09766 |
| C | 2.04796 | -1.15599 | -0.24103 |
| C | 2.72042 | -2.3773 | -0.18763 |
| C | 1.96994 | 1.34187 | -0.20215 |
| C | 0.46967 | 1.25798 | -0.13907 |
| C | -0.20663 | 0.03026 | -0.13054 |
| C | 0.57641 | -1.18662 | -0.48837 |
| C | -0.26388 | 2.45384 | -0.06569 |
| C | -1.6484 | 2.42151 | 0.00207 |
| C | -2.34214 | 1.20035 | 0.05652 |
| C | -1.612 | -0.00195 | 0.03674 |
| C | -3.87111 | 1.23145 | 0.06122 |
| C | -4.52687 | -0.06785 | 0.57442 |
| C | -3.81899 | -1.28934 | -0.03693 |
| C | -2.35298 | -1.25549 | 0.3869 |
| O | 0.0468 | -2.15333 | -1.03062 |
| O | 2.50005 | 2.448 | -0.30566 |
| O | -1.87189 | -2.13309 | 1.08963 |
| O | -3.93694 | -1.1841 | -1.46612 |
| C | -4.55861 | -0.13313 | 2.11022 |
| O | -4.3217 | 1.56964 | -1.26332 |
| O | 4.79482 | 1.21214 | 0.25331 |
| C | 6.20661 | 1.20978 | 0.5068 |
| H | 4.62912 | -3.33638 | 0.09931 |
| H | 5.87047 | -1.2397 | 0.36415 |
| H | 2.16286 | -3.29878 | -0.3117 |
| H | 0.2706 | 3.39753 | -0.07578 |
| H | -2.2099 | 3.35149 | 0.02839 |
| H | -4.20321 | 2.06692 | 0.68418 |
| H | -5.55991 | -0.07365 | 0.20604 |
| H | -4.26623 | -2.22366 | 0.31865 |
| H | -3.54594 | -1.96746 | -1.88803 |
| H | -5.16321 | 0.68959 | 2.50583 |
| H | -3.55943 | -0.05539 | 2.55457 |
| H | -5.00488 | -1.0741 | 2.44993 |
| H | -4.17683 | 0.77787 | -1.81606 |
| H | 6.4826 | 2.26134 | 0.58654 |
| H | 6.43668 | 0.69554 | 1.44597 |
| H | 6.75343 | 0.74717 | -0.32164 |

Standard orientation of rubiginone J (**1**) 2*R*, 3*S*, 4*S*_con3:


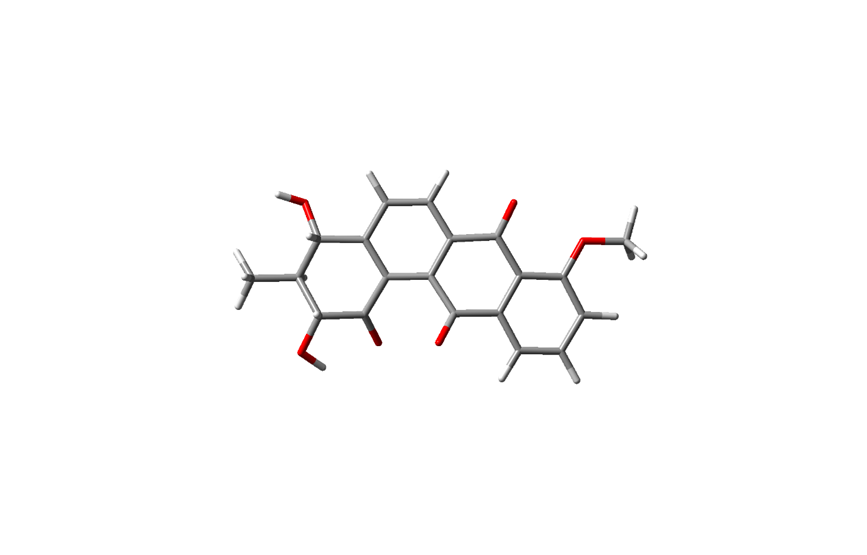


| C | -4.15938 | 2.40999 | -0.12682 |
| --- | --- | --- | --- |
| C | -4.86958 | 1.22444 | -0.29474 |
| C | -4.22665 | -0.02166 | -0.17156 |
| C | -2.83079 | -0.06707 | 0.10507 |
| C | -2.13916 | 1.15907 | 0.26466 |
| C | -2.79544 | 2.38608 | 0.16465 |
| C | -2.08153 | -1.33789 | 0.26032 |
| C | -0.57872 | -1.26566 | 0.25591 |
| C | 0.10796 | -0.0449 | 0.25306 |
| C | -0.68142 | 1.17709 | 0.58302 |
| C | 0.14854 | -2.4672 | 0.2318 |
| C | 1.53454 | -2.45076 | 0.2118 |
| C | 2.24032 | -1.23628 | 0.15162 |
| C | 1.51955 | -0.02741 | 0.12686 |
| C | 3.76174 | -1.25957 | 0.17535 |
| C | 4.42205 | 0.00472 | -0.40794 |
| C | 3.73518 | 1.25735 | 0.1905 |
| C | 2.26738 | 1.21661 | -0.19913 |
| O | -0.17401 | 2.13113 | 1.16604 |
| O | -2.6233 | -2.4389 | 0.3562 |
| O | 1.79683 | 2.13062 | -0.86857 |
| O | 4.32709 | 2.45538 | -0.27218 |
| C | 5.93466 | 0.02749 | -0.16219 |
| O | 4.19988 | -2.43927 | -0.50765 |
| O | -4.88427 | -1.1899 | -0.31896 |
| C | -6.28336 | -1.17846 | -0.63515 |
| H | -4.67881 | 3.35889 | -0.22523 |
| H | -5.92803 | 1.27075 | -0.51783 |
| H | -2.23512 | 3.30389 | 0.30311 |
| H | -0.39363 | -3.4065 | 0.24567 |
| H | 2.09139 | -3.38088 | 0.21639 |
| H | 4.05208 | -1.338 | 1.23589 |
| H | 4.23666 | 0.00711 | -1.49164 |
| H | 3.8133 | 1.2206 | 1.28896 |
| H | 3.62197 | 2.93586 | -0.75334 |
| H | 6.37213 | 0.94371 | -0.56758 |
| H | 6.42474 | -0.82016 | -0.6533 |
| H | 6.16192 | -0.01557 | 0.91107 |
| H | 5.06254 | -2.70476 | -0.15253 |
| H | -6.46614 | -0.67868 | -1.59237 |
| H | -6.5664 | -2.22856 | -0.70907 |
| H | -6.86154 | -0.69612 | 0.16007 |

**Fig. S22.** UV spectra of compounds **1**–**8**.


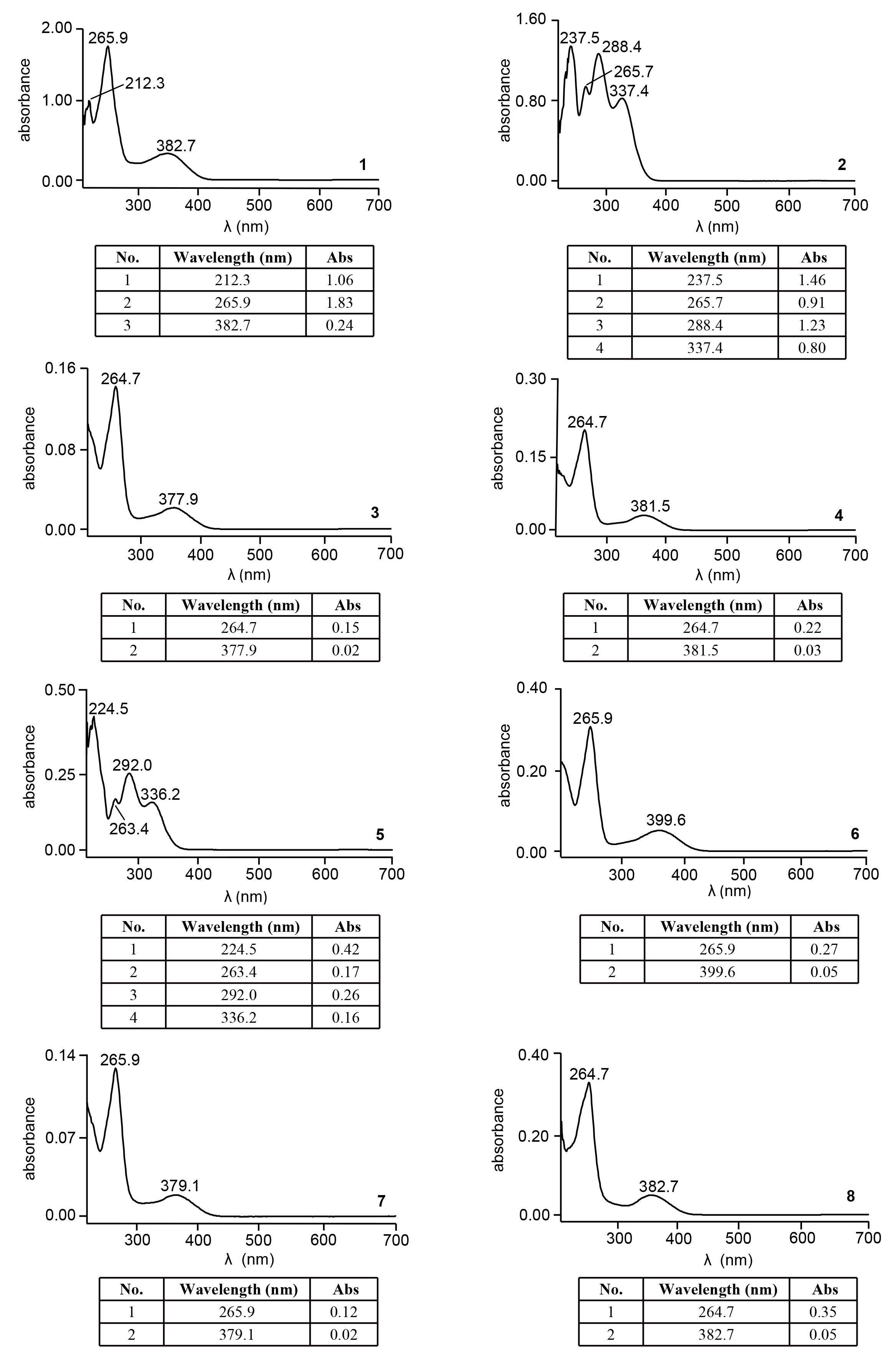


**Fig. S23.** ^1^H NMR spectrum of compound **2** in CD_3_OD (400 MHz).

**Fig. S24.** ^13^C NMR spectrum of compound **2** in CD_3_OD (101 MHz).

**Fig. S25.** DEPT 135 spectrum of compound **2** (CD_3_OD).

**Fig. S26.** ^1^H-^1^H COSY spectrum of compound **2** (CD_3_OD).


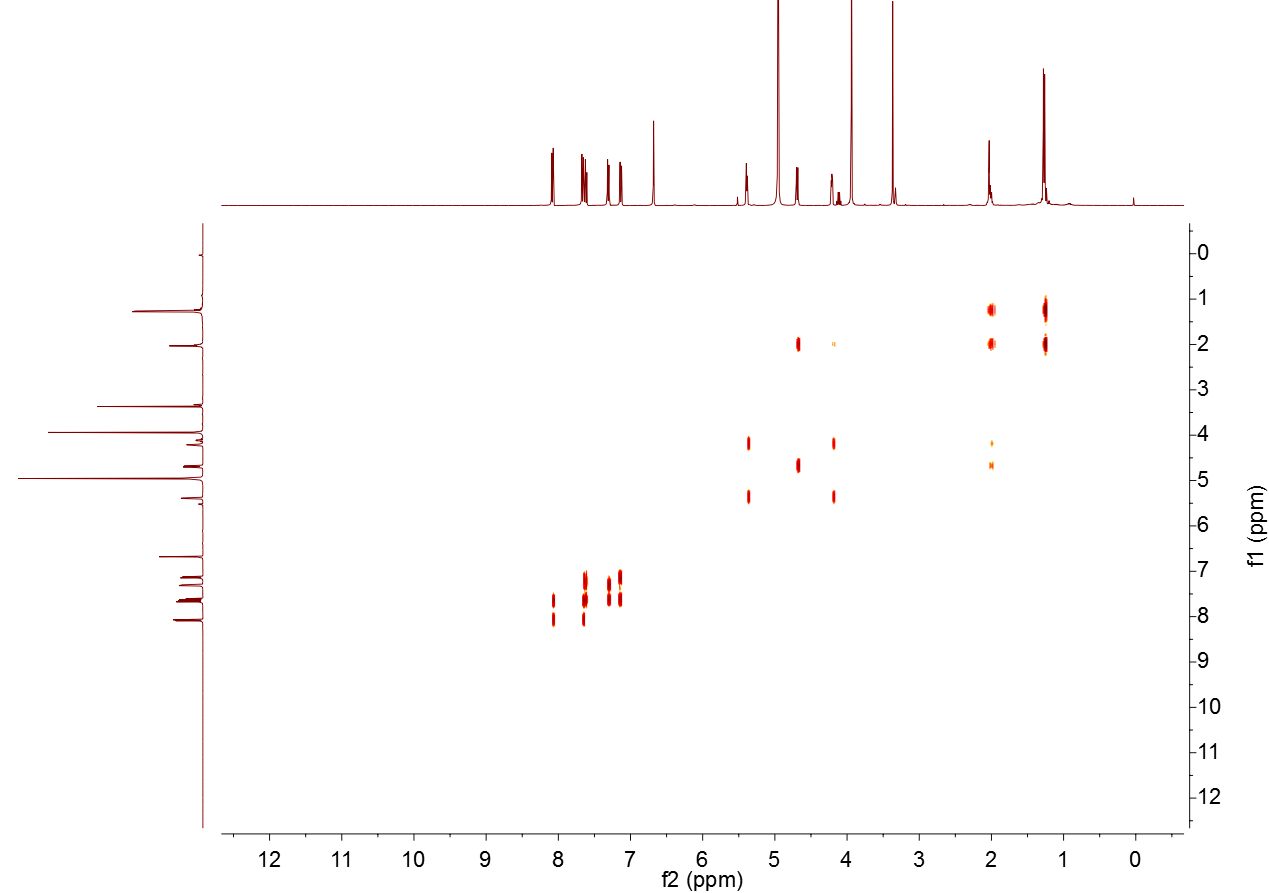


**Fig. S27.** ^1^H–^13^C HSQC spectrum of compound **2** (CD_3_OD).


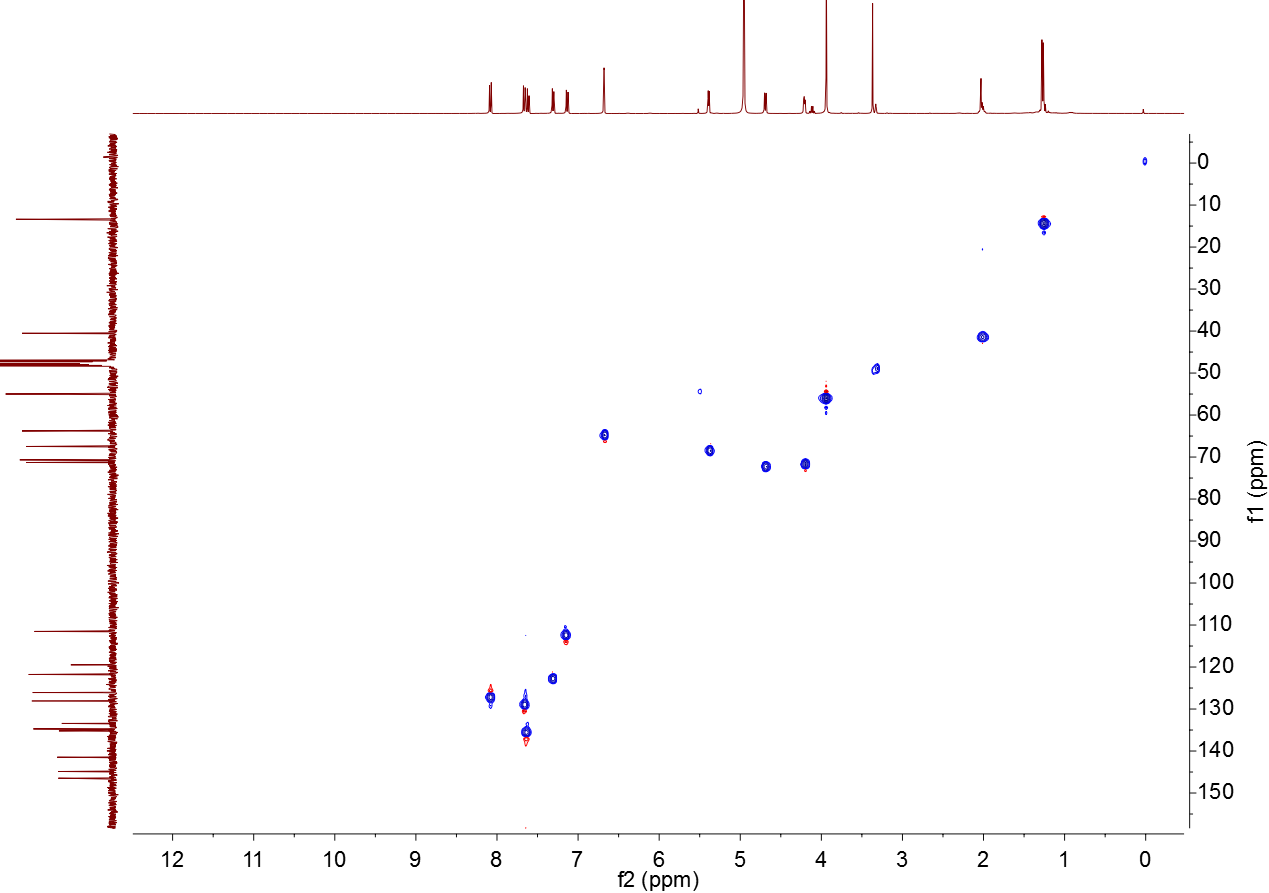


**Fig. S28.** ^1^H–^13^C HMBC spectrum of compound **2** (CD_3_OD).


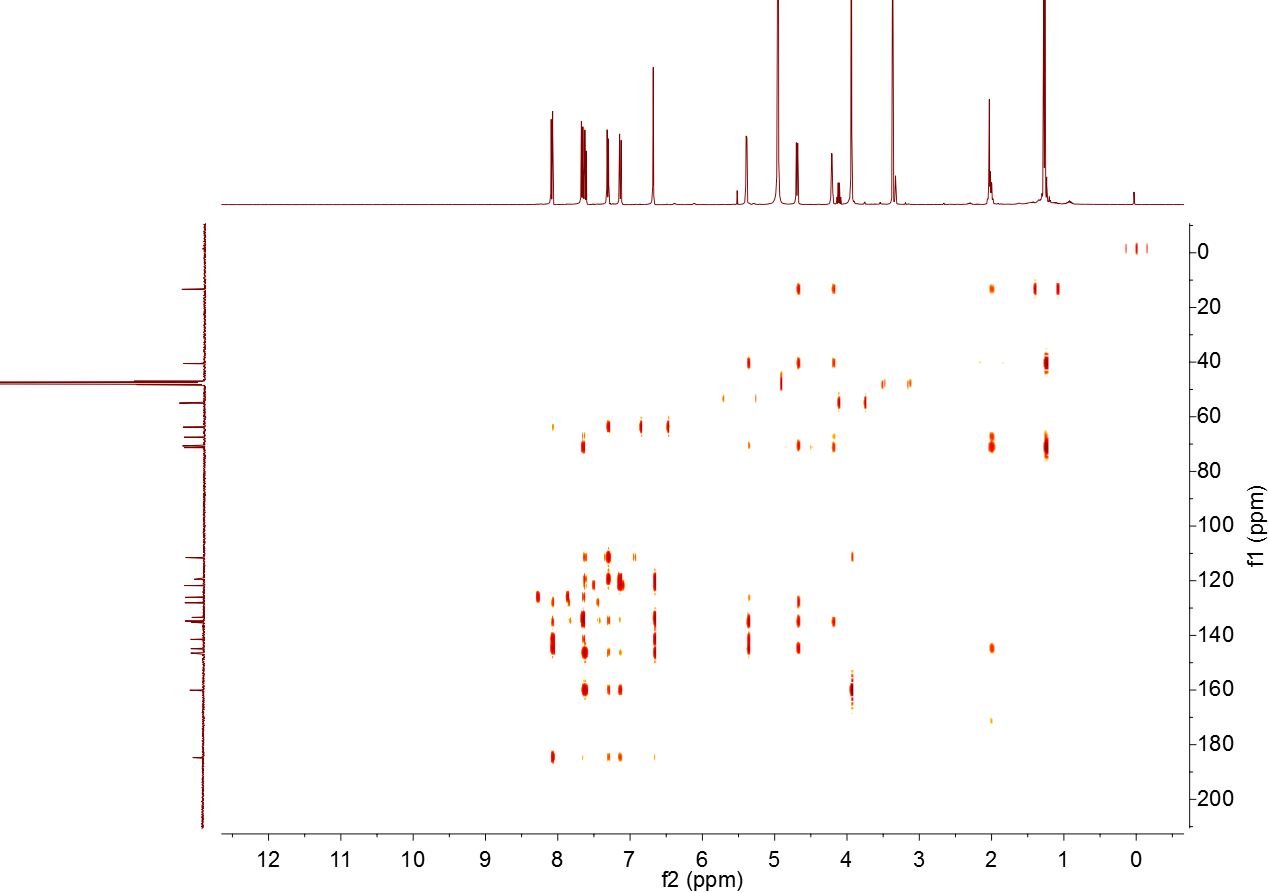


**Fig. S29.** ^1^H–^1^H NOESY spectrum of compound **2** (CD_3_OD).

**Fig. S30.** HR-ESI-MS spectra of compounds **5**–**8**.

**Fig. S31.** ^1^H NMR spectrum of compound **5** in CD_3_OD (500 MHz).

**Fig. S32.** ^13^C NMR spectrum of compound **5** in CD_3_OD (126 MHz).

**Fig. S33.** DEPT 135 spectrum of compound **5** (CD_3_OD).

**Fig. S34.** ^1^H–^1^H COSY spectrum of compound **5** (CD_3_OD).


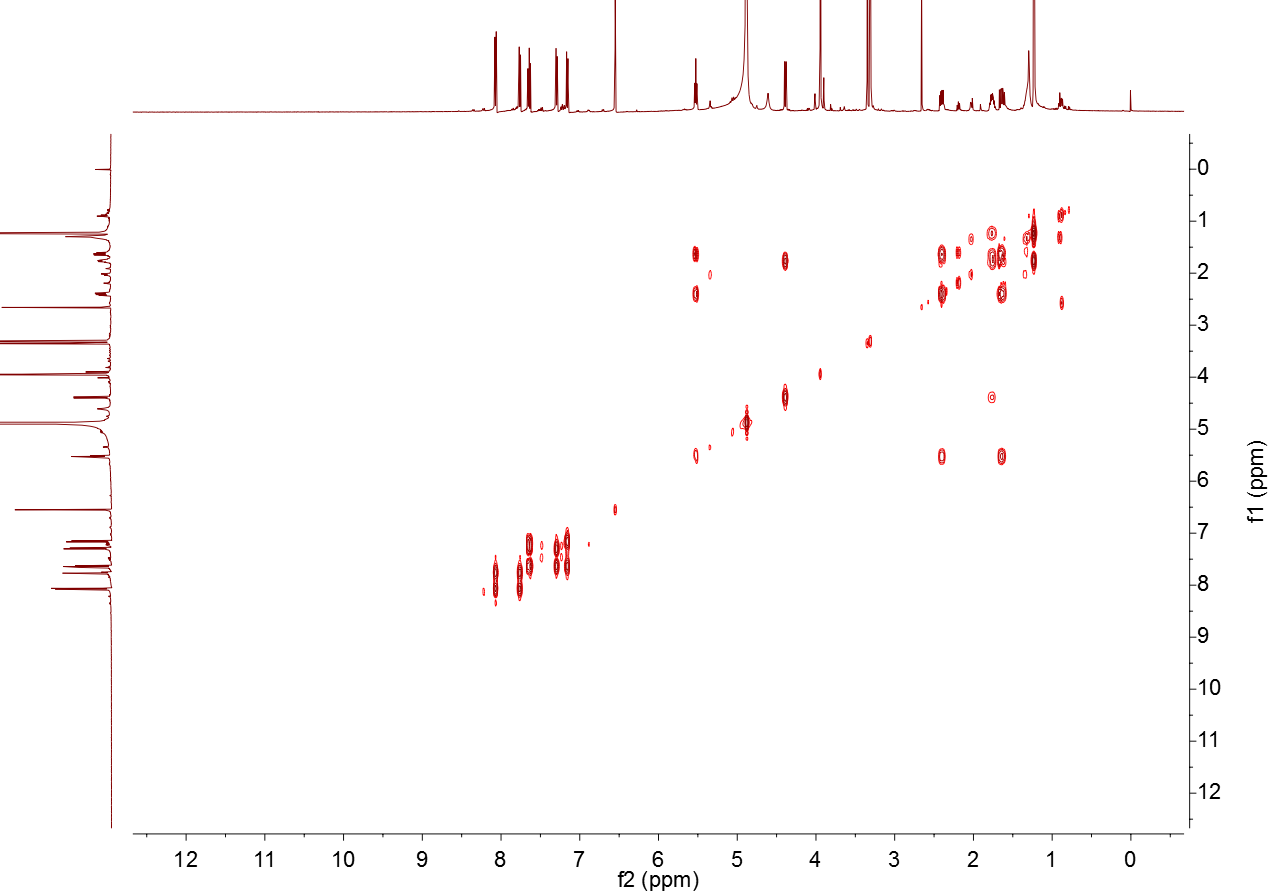


**Fig. S35.** ^1^H–^13^C HSQC spectrum of compound **5** (CD_3_OD).


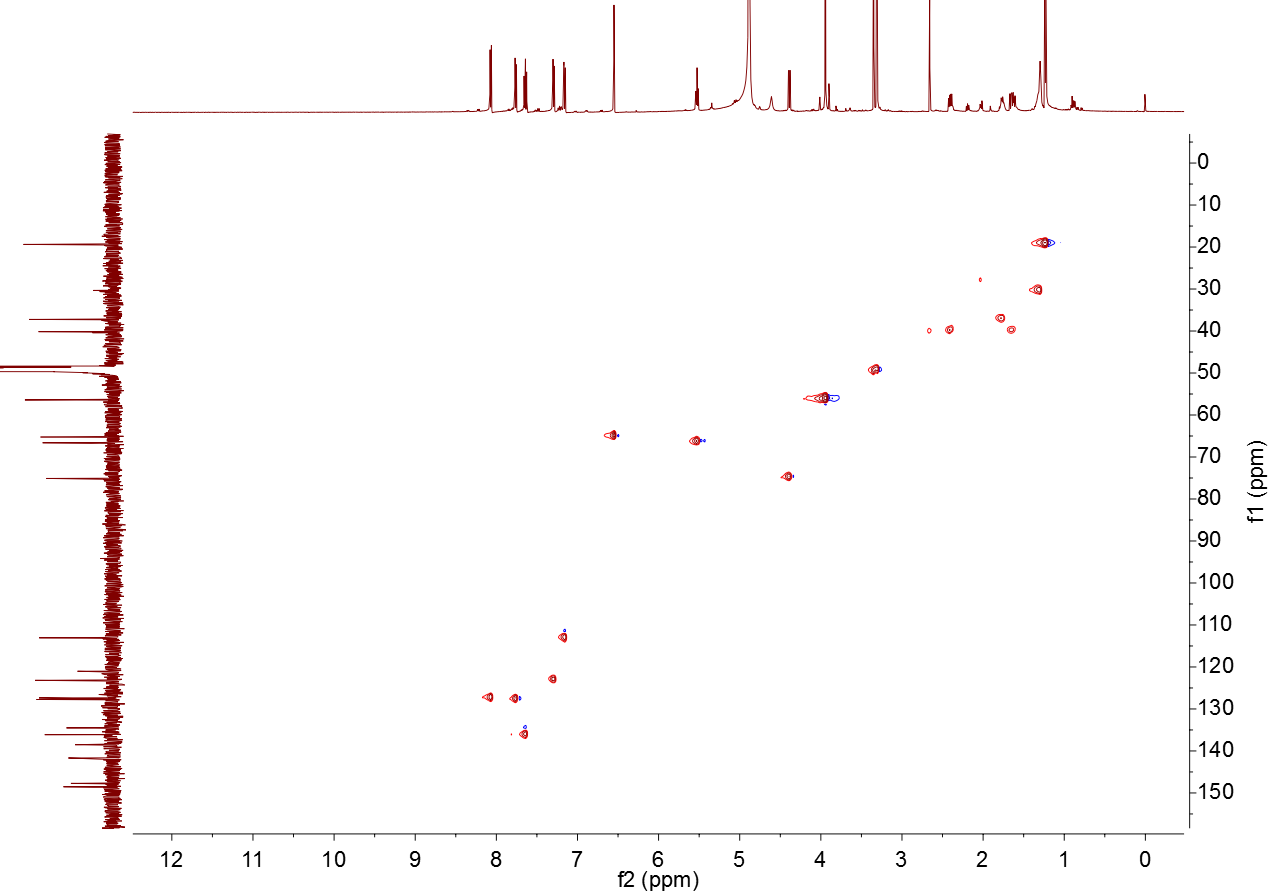


**Fig. S36.** ^1^H–^13^C HMBC spectrum of compound **5** (CD_3_OD).


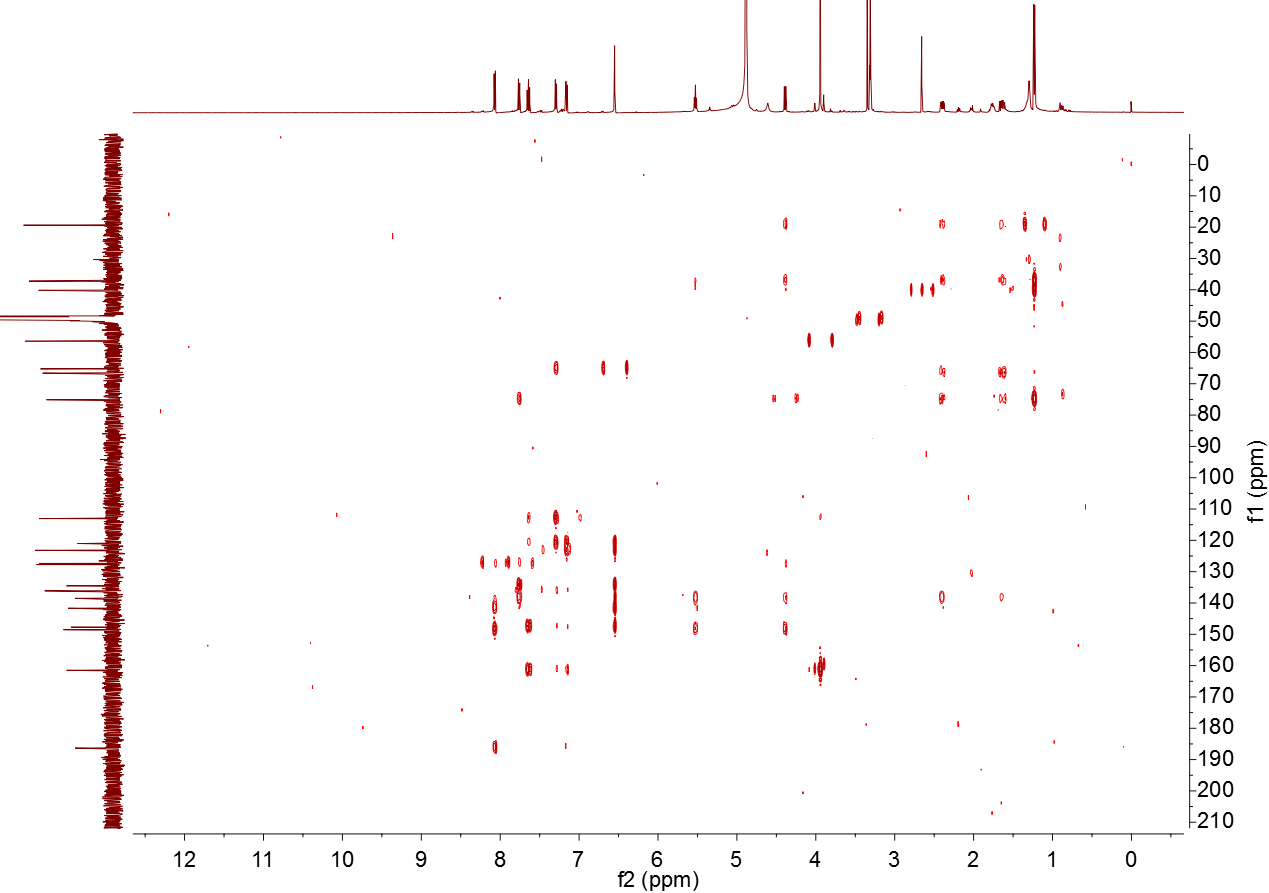


**Fig. S37.** ^13^C NMR spectrum of compound **7** in CD_3_OD (150 MHz).

**Fig. S38.** DEPT 135 spectrum of compound **7** (CD_3_OD).

**Fig. S39.** ^1^H NMR spectrum of compound **7** in CD_3_OD (600 MHz).

**Fig. S40.** ^1^H–^1^H COSY spectrum of compound **7** (CD_3_OD).

**Fig. S41.** ^1^H–^13^C HSQC spectrum of compound **7** (CD_3_OD).

**Fig. S42.** ^1^H–^13^C HMBC spectrum of compound **7** (CD_3_OD).

**Fig. S43.** ^1^H–^1^H NOESY spectrum of compound **7** (CD_3_OD).

**Fig. S44.** The energy lowest conformers and populations of rubiginone M (**7**) 2*S*, 3*S* and optimized coordinates.

A random conformational search was performed for rubiginone M (**7**) 2*S*, 3*S* and rubiginone M (**7**) 2*R*, 3*S* using SYBYL 2.0 software package with MMFF94s molecular mechanics force field after the energy minimization, which yielded 12 conformers for rubiginone M (**7**) 2*S*, 3*S* and 8 for rubiginone M (**7**) 2*R*, 3*S* respectively, within a 10 kcal/mol energy window. The following geometry optimizations at the B3LYP/6-31 + G (d) level afforded 4 conformers for rubiginone M (**7**) 2*S*, 3*S* (Fig. S44) and 4 for rubiginone M (**7**) 2*R*, 3*S* (Fig. S45), within a 10 kcal/mol energy window. Through the same process and calculation, the comparison of the experimental ECD spectrum of rubiginone M (**7**) with the calculated ones (Fig. 5c) revealed that the CEs of rubiginone M (**7**) 2*S*, 3*S* were in good accordance with the experimental CEs of rubiginone M (**7**) in the region 250–600 nm


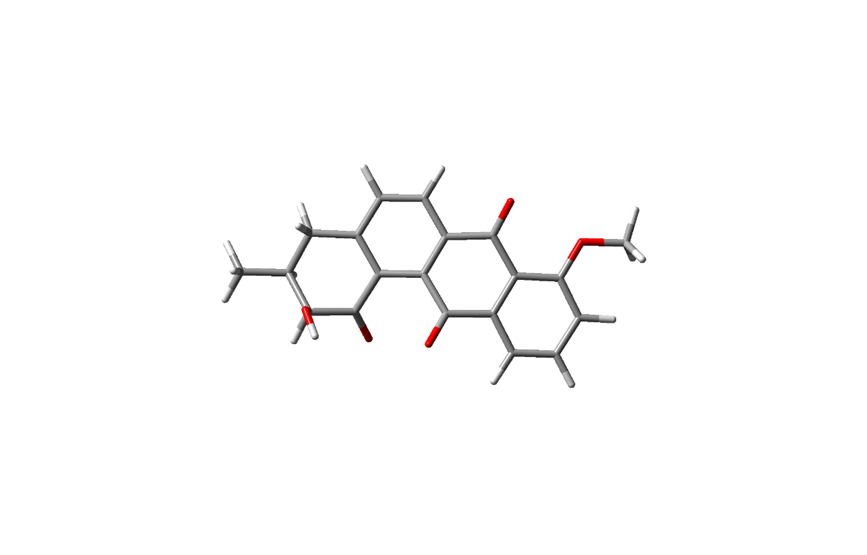

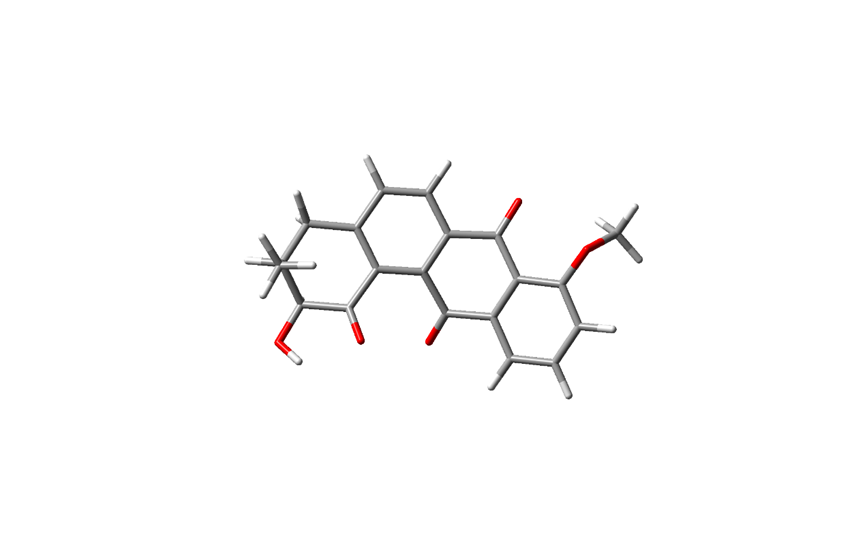


Rubiginone M **(7)** 2*S*, 3*S*_con1:0.81% Rubiginone M **(7)** 2*S*, 3*S*_con2: 0.66%


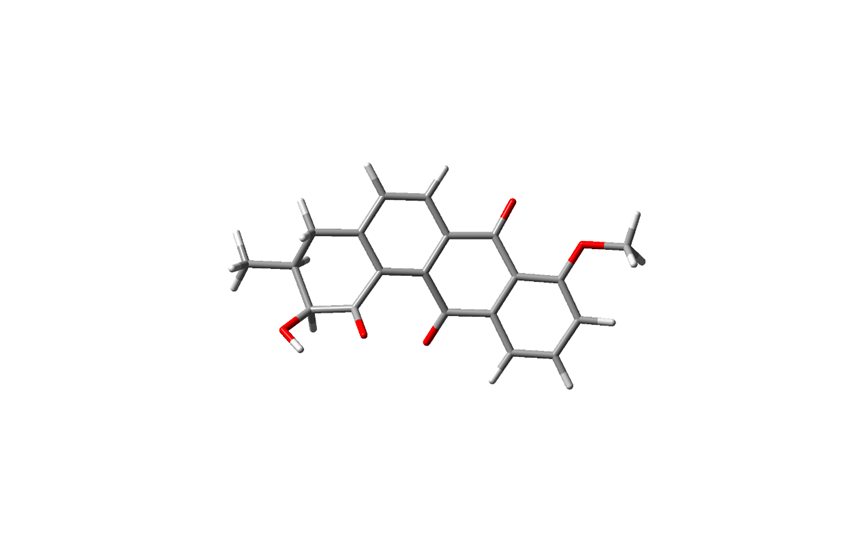

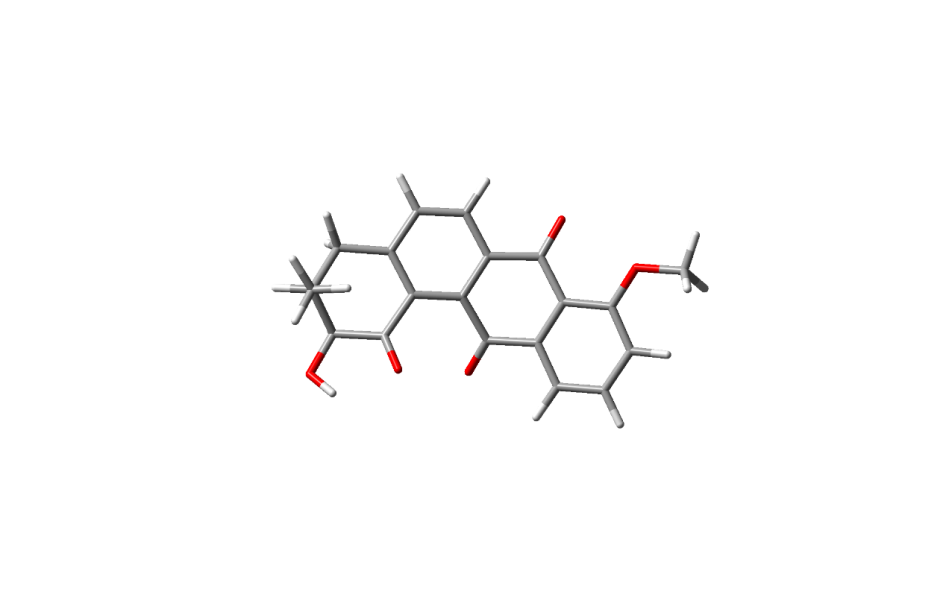


Rubiginone M **(7)** 2*S*, 3*S*_con3: 3.16% Rubiginone M **(7)** 2*S*, 3*S*_con4: 95.37%

Standard orientation of rubiginone M **(7)** 2*S*, 3*S*_con1:


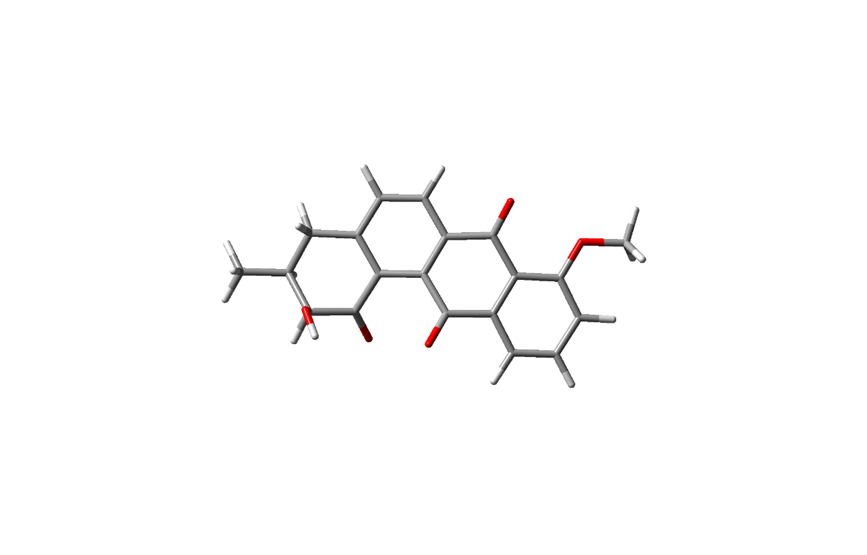


| C | 3.89419 | -2.44812 | -0.14858 |
| --- | --- | --- | --- |
| C | 4.64079 | -1.27606 | -0.23448 |
| C | 4.02198 | -0.019 | -0.10098 |
| C | 2.61512 | 0.05295 | 0.10442 |
| C | 1.88595 | -1.15925 | 0.17933 |
| C | 2.51731 | -2.39868 | 0.06826 |
| C | 1.88951 | 1.33797 | 0.26803 |
| C | 0.39108 | 1.30716 | 0.17776 |
| C | -0.32696 | 0.10543 | 0.10154 |
| C | 0.41118 | -1.1511 | 0.41242 |
| C | -0.30436 | 2.52775 | 0.15008 |
| C | -1.68711 | 2.5443 | 0.05453 |
| C | -2.4249 | 1.35162 | -0.06557 |
| C | -1.72985 | 0.12712 | -0.0847 |
| C | -3.93714 | 1.42053 | -0.13469 |
| C | -4.63419 | 0.14975 | -0.65381 |
| C | -3.97271 | -1.09812 | -0.04471 |
| C | -2.51012 | -1.08818 | -0.48137 |
| O | -0.15399 | -2.12389 | 0.90625 |
| O | 2.45631 | 2.41752 | 0.44116 |
| O | -2.05866 | -1.94897 | -1.22583 |
| O | -4.08514 | -1.03132 | 1.38085 |
| C | -6.14751 | 0.18879 | -0.4101 |
| O | 4.71483 | 1.13664 | -0.17441 |
| C | 6.12663 | 1.09896 | -0.42351 |
| H | 4.3953 | -3.4062 | -0.2533 |
| H | 5.70844 | -1.34204 | -0.40191 |
| H | 1.92786 | -3.30574 | 0.14074 |
| H | 0.25848 | 3.45266 | 0.21593 |
| H | -2.21492 | 3.49462 | 0.05602 |
| H | -4.23711 | 2.27883 | -0.74704 |
| H | -4.29711 | 1.6339 | 0.88203 |
| H | -4.4635 | 0.08188 | -1.73807 |
| H | -4.44207 | -2.01106 | -0.43027 |
| H | -3.77588 | -1.87015 | 1.75988 |
| H | -6.63633 | -0.69147 | -0.84348 |
| H | -6.37504 | 0.21612 | 0.66092 |
| H | -6.5848 | 1.08051 | -0.87455 |
| H | 6.34365 | 0.62414 | -1.38632 |
| H | 6.43841 | 2.14322 | -0.45037 |
| H | 6.65442 | 0.5779 | 0.38237 |

Standard orientation of Rubiginone M (**7**) 2*S*, 3*S*_con2:


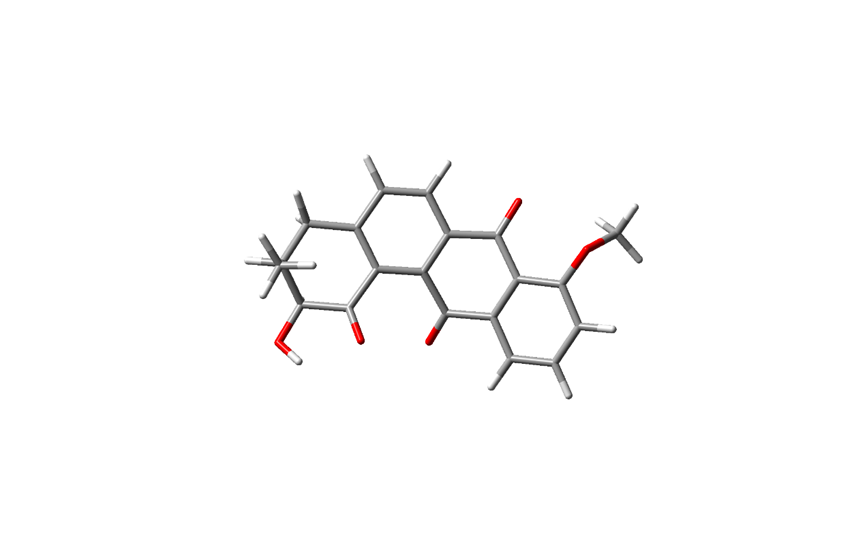


| C | 3.70673 | -2.62714 | 0.29776 |
| --- | --- | --- | --- |
| C | 4.4667 | -1.49775 | 0.59722 |
| C | 3.92346 | -0.21255 | 0.46729 |
| C | 2.58396 | -0.05005 | 0.0489 |
| C | 1.8201 | -1.21117 | -0.22237 |
| C | 2.38227 | -2.48534 | -0.12037 |
| C | 1.9524 | 1.28447 | -0.14883 |
| C | 0.45543 | 1.33425 | -0.20894 |
| C | -0.3199 | 0.16913 | -0.30203 |
| C | 0.39681 | -1.09563 | -0.653 |
| C | -0.17818 | 2.58712 | -0.15356 |
| C | -1.56064 | 2.67436 | -0.19387 |
| C | -2.3616 | 1.51889 | -0.24036 |
| C | -1.73289 | 0.25605 | -0.24891 |
| C | -3.8683 | 1.6598 | -0.32549 |
| C | -4.65776 | 0.44094 | 0.18497 |
| C | -4.04853 | -0.82493 | -0.46796 |
| C | -2.59566 | -0.94067 | -0.03477 |
| O | -0.13424 | -1.9708 | -1.32967 |
| O | 2.60294 | 2.32326 | -0.24265 |
| O | -2.22576 | -1.93919 | 0.5751 |
| O | -4.75999 | -2.00145 | -0.13905 |
| C | -4.70491 | 0.36628 | 1.71781 |
| O | 4.69452 | 0.85568 | 0.8423 |
| C | 5.61 | 1.34557 | -0.15511 |
| H | 4.14257 | -3.61643 | 0.40235 |
| H | 5.49126 | -1.59254 | 0.94481 |
| H | 1.7739 | -3.35338 | -0.35072 |
| H | 0.43275 | 3.48113 | -0.08747 |
| H | -2.03977 | 3.64939 | -0.17032 |
| H | -4.11652 | 1.82864 | -1.38452 |
| H | -4.1839 | 2.56405 | 0.20641 |
| H | -5.68669 | 0.51961 | -0.18654 |
| H | -4.0714 | -0.69037 | -1.56075 |
| H | -4.13239 | -2.57811 | 0.34217 |
| H | -5.2485 | 1.2306 | 2.11545 |
| H | -3.70388 | 0.37003 | 2.16712 |
| H | -5.22133 | -0.54044 | 2.04846 |
| H | 6.29898 | 0.55274 | -0.46904 |
| H | 6.16948 | 2.15212 | 0.32139 |
| H | 5.05882 | 1.73329 | -1.01738 |

Standard orientation of rubiginone M (**7**) 2*S*, 3*S*_con3:


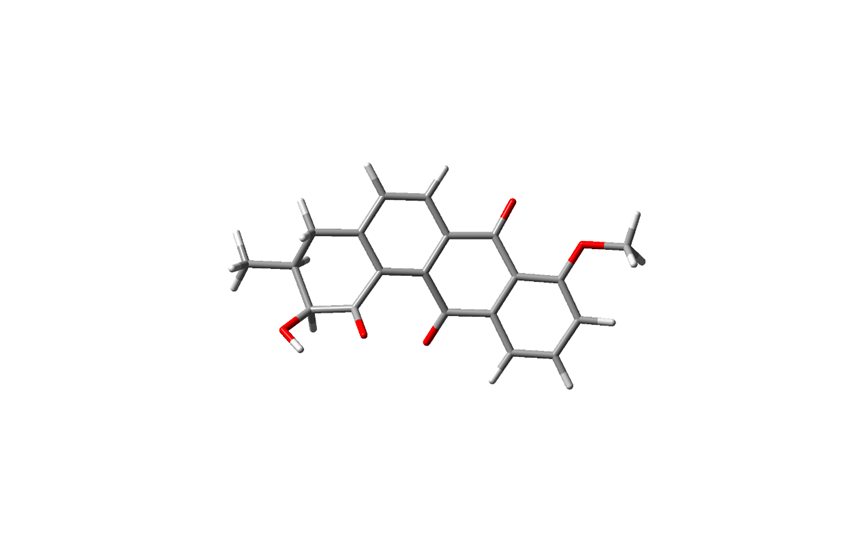


| C | -3.87901 | -2.50122 | 0.09157 |
| --- | --- | --- | --- |
| C | -4.651 | -1.35163 | -0.05121 |
| C | -4.05319 | -0.07749 | -0.02972 |
| C | -2.64227 | 0.03434 | 0.12518 |
| C | -1.88561 | -1.15748 | 0.25575 |
| C | -2.49671 | -2.41223 | 0.25237 |
| C | -1.94416 | 1.34375 | 0.17865 |
| C | -0.44826 | 1.34098 | 0.05306 |
| C | 0.29427 | 0.15045 | 0.05987 |
| C | -0.40283 | -1.11376 | 0.42071 |
| C | 0.21677 | 2.57017 | -0.08137 |
| C | 1.60012 | 2.61553 | -0.20066 |
| C | 2.35823 | 1.43423 | -0.22676 |
| C | 1.69196 | 0.1982 | -0.12575 |
| C | 3.86701 | 1.44475 | -0.33319 |
| C | 4.46648 | 0.37021 | 0.59772 |
| C | 3.94666 | -1.03649 | 0.21283 |
| C | 2.54413 | -0.99124 | -0.41095 |
| O | 0.21437 | -2.07408 | 0.87701 |
| O | -2.53197 | 2.41912 | 0.29735 |
| O | 2.22964 | -1.84827 | -1.22932 |
| O | 4.82617 | -1.69061 | -0.6953 |
| C | 5.99588 | 0.43432 | 0.66948 |
| O | -4.77078 | 1.05673 | -0.16708 |
| C | -6.18834 | 0.97638 | -0.36954 |
| H | -4.36423 | -3.47289 | 0.07405 |
| H | -5.72219 | -1.44824 | -0.17592 |
| H | -1.88658 | -3.3013 | 0.36427 |
| H | -0.36814 | 3.48364 | -0.07305 |
| H | 2.10502 | 3.57466 | -0.27898 |
| H | 4.24714 | 2.43573 | -0.06353 |
| H | 4.18247 | 1.25957 | -1.37069 |
| H | 4.07437 | 0.57078 | 1.60356 |
| H | 3.85471 | -1.64169 | 1.13096 |
| H | 4.27306 | -2.30436 | -1.21589 |
| H | 6.44953 | 0.28368 | -0.31494 |
| H | 6.31068 | 1.4135 | 1.04823 |
| H | 6.3909 | -0.33265 | 1.34571 |
| H | -6.52062 | 2.01024 | -0.46484 |
| H | -6.68233 | 0.5087 | 0.48875 |
| H | -6.42412 | 0.42673 | -1.28704 |

Standard orientation of rubiginone M (**7**) 2*S*, 3*S*_con4:


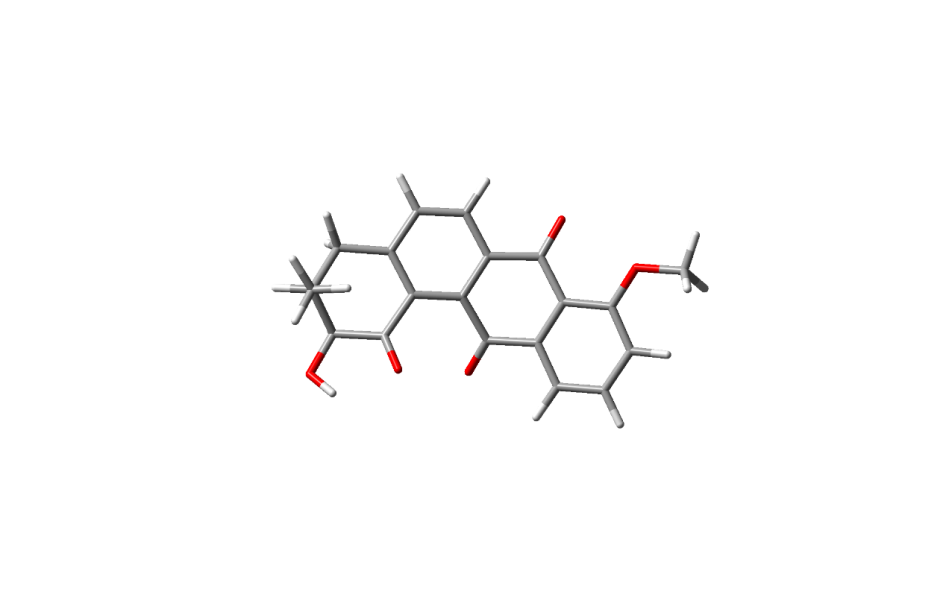


| C | 3.73578 | -2.52524 | 0.15136 |
| --- | --- | --- | --- |
| C | 4.5063 | -1.385 | 0.36332 |
| C | 3.94329 | -0.10211 | 0.22818 |
| C | 2.56634 | 0.02781 | -0.10811 |
| C | 1.81068 | -1.15347 | -0.30708 |
| C | 2.38872 | -2.41831 | -0.19461 |
| C | 1.89916 | 1.34146 | -0.28923 |
| C | 0.39674 | 1.35814 | -0.32336 |
| C | -0.36128 | 0.17976 | -0.35127 |
| C | 0.36766 | -1.08015 | -0.68063 |
| C | -0.25916 | 2.60081 | -0.2985 |
| C | -1.64362 | 2.6633 | -0.30543 |
| C | -2.42546 | 1.49396 | -0.28137 |
| C | -1.77408 | 0.2436 | -0.25855 |
| C | -3.93662 | 1.60515 | -0.32424 |
| C | -4.68625 | 0.3951 | 0.26198 |
| C | -4.07433 | -0.88609 | -0.35779 |
| C | -2.60624 | -0.95771 | 0.03231 |
| O | -0.17013 | -1.99112 | -1.30369 |
| O | 2.51038 | 2.40652 | -0.37728 |
| O | -2.19925 | -1.92343 | 0.67115 |
| O | -4.75171 | -2.06061 | 0.04306 |
| C | -4.6813 | 0.38455 | 1.79736 |
| O | 4.66025 | 1.02443 | 0.42076 |
| C | 6.03841 | 0.92757 | 0.80621 |
| H | 4.19449 | -3.50391 | 0.25956 |
| H | 5.54973 | -1.496 | 0.63025 |
| H | 1.78129 | -3.30003 | -0.36551 |
| H | 0.33722 | 3.50672 | -0.28308 |
| H | -2.1391 | 3.63053 | -0.30863 |
| H | -4.222 | 1.72449 | -1.38067 |
| H | -4.25232 | 2.5253 | 0.17965 |
| H | -5.72812 | 0.43995 | -0.07776 |
| H | -4.13513 | -0.79835 | -1.45391 |
| H | -4.09683 | -2.60452 | 0.52621 |
| H | -5.22773 | 1.25531 | 2.17672 |
| H | -3.66587 | 0.42532 | 2.21107 |
| H | -5.16899 | -0.51631 | 2.18319 |
| H | 6.14322 | 0.40669 | 1.76395 |
| H | 6.37769 | 1.95839 | 0.90943 |
| H | 6.62765 | 0.42149 | 0.03415 |

**Fig. S45**. The energy lowest conformers and populations of rubiginone M **(7)** 2*R*, 3*S* and optimized coordinates.
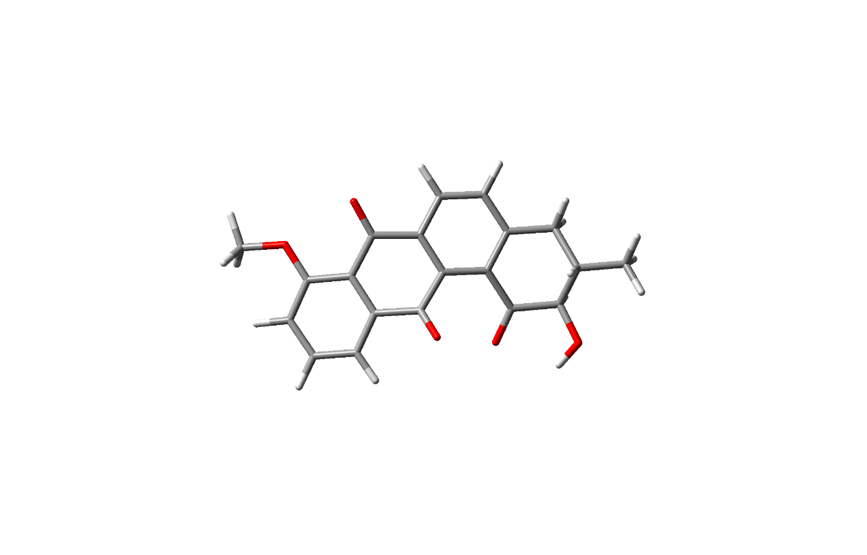

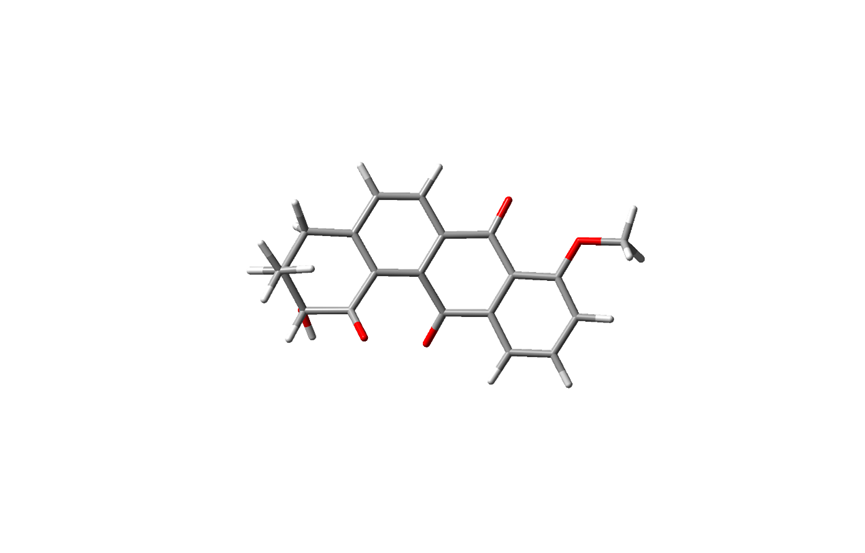


Rubiginone M (**7**) 2*R*, 3*S*_con1: 0.04% Rubiginone M (**7**) 2*R*, 3*S*_con2: 0.48%


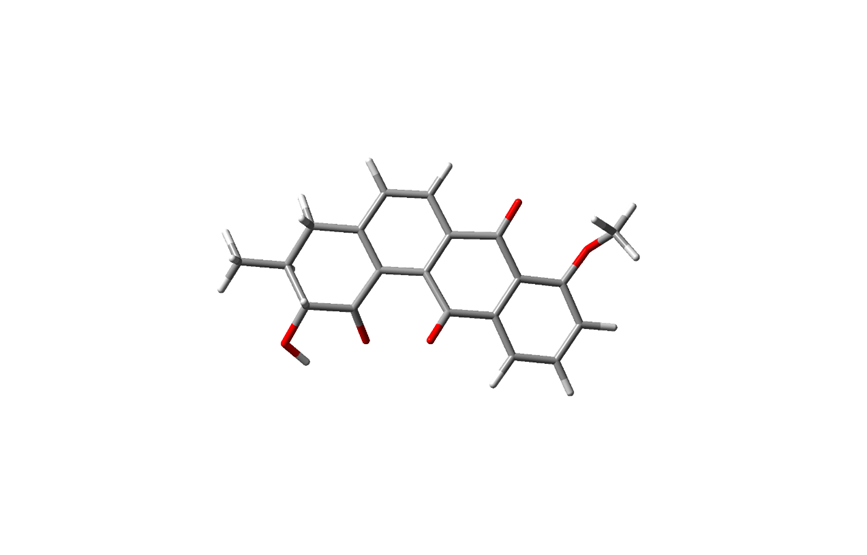

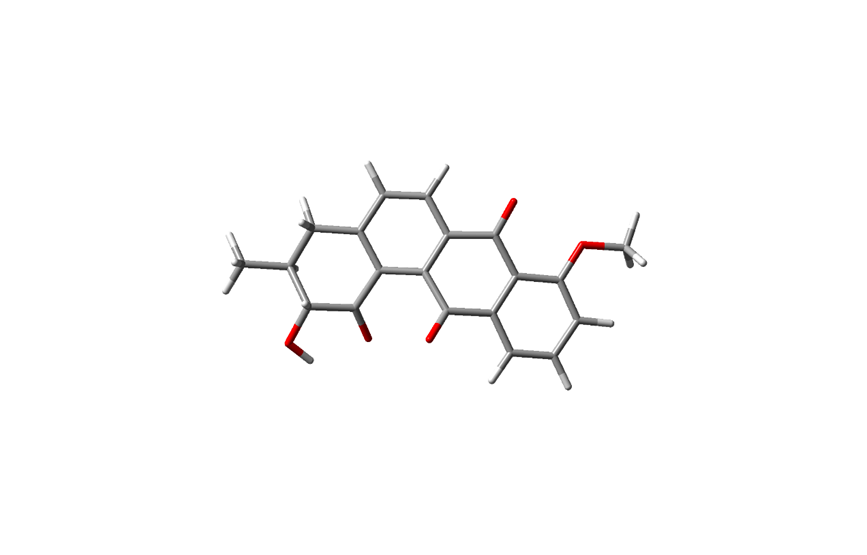


Rubiginone M (**7**) 2*R*, 3*S*_con3: 0.68% Rubiginone M (**7**) 2*R*, 3*S*_con4: 98.80%

Standard orientation of rubiginone M (**7**) 2*R*, 3*S*_con1:


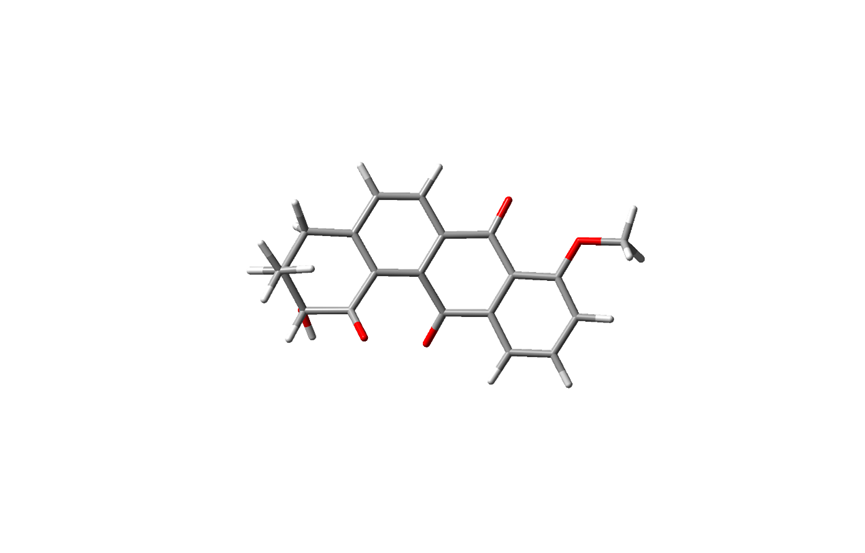


| C | 3.80903 | -2.4552 | 0.21508 |
| --- | --- | --- | --- |
| C | 4.55569 | -1.28497 | 0.32228 |
| C | 3.94626 | -0.02668 | 0.16021 |
| C | 2.5476 | 0.04881 | -0.09487 |
| C | 1.81853 | -1.16184 | -0.19294 |
| C | 2.44139 | -2.40261 | -0.05328 |
| C | 1.8315 | 1.33513 | -0.28722 |
| C | 0.33021 | 1.30697 | -0.26238 |
| C | -0.39351 | 0.10769 | -0.20875 |
| C | 0.3547 | -1.15093 | -0.48701 |
| C | -0.36299 | 2.52936 | -0.27216 |
| C | -1.74835 | 2.54965 | -0.23862 |
| C | -2.49416 | 1.35979 | -0.14122 |
| C | -1.80341 | 0.13332 | -0.08005 |
| C | -4.00814 | 1.43228 | -0.15815 |
| C | -4.73652 | 0.18636 | 0.38333 |
| C | -4.05323 | -1.08323 | -0.15047 |
| C | -2.59653 | -1.07568 | 0.30643 |
| O | -0.18971 | -2.12152 | -1.00738 |
| O | 2.4064 | 2.41466 | -0.43156 |
| O | -2.1584 | -1.94099 | 1.05419 |
| O | -4.13658 | -1.05926 | -1.58089 |
| C | -4.82776 | 0.18149 | 1.91806 |
| O | 4.64068 | 1.12676 | 0.25026 |
| C | 6.04415 | 1.08544 | 0.54237 |
| H | 4.30293 | -3.41422 | 0.34293 |
| H | 5.61638 | -1.35329 | 0.52852 |
| H | 1.85232 | -3.30819 | -0.14495 |
| H | 0.20451 | 3.45257 | -0.31858 |
| H | -2.27326 | 3.50096 | -0.27185 |
| H | -4.30299 | 1.59233 | -1.20413 |
| H | -4.33916 | 2.32272 | 0.38861 |
| H | -5.7572 | 0.18304 | -0.01958 |
| H | -4.53616 | -1.98185 | 0.25057 |
| H | -3.80117 | -1.9019 | -1.92906 |
| H | -5.41309 | 1.04236 | 2.2585 |
| H | -5.32078 | -0.72756 | 2.28153 |
| H | -3.84166 | 0.24278 | 2.39425 |
| H | 6.5939 | 0.55683 | -0.24368 |
| H | 6.23024 | 0.61651 | 1.51448 |
| H | 6.35907 | 2.12869 | 0.57163 |

Standard orientation of rubiginone M (**7**) 2*R*, 3*S*_con2:


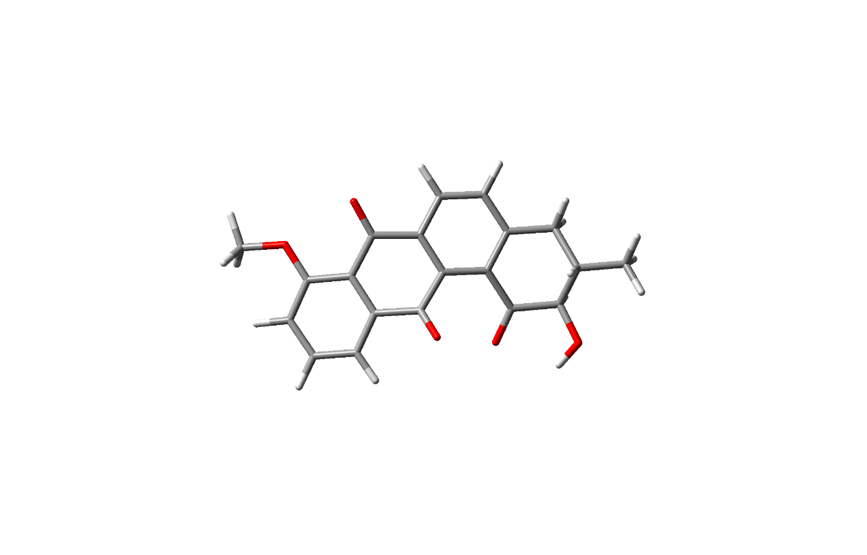


| C | 3.82094 | -2.50418 | 0.05602 |
| --- | --- | --- | --- |
| C | 4.59353 | -1.36245 | 0.25475 |
| C | 4.0204 | -0.08101 | 0.15377 |
| C | 2.63204 | 0.04379 | -0.1306 |
| C | 1.87564 | -1.13767 | -0.31589 |
| C | 2.46184 | -2.40129 | -0.24276 |
| C | 1.94682 | 1.35161 | -0.27994 |
| C | 0.44408 | 1.35591 | -0.21158 |
| C | -0.30537 | 0.17217 | -0.19413 |
| C | 0.42214 | -1.05483 | -0.64442 |
| C | -0.21507 | 2.59643 | -0.1353 |
| C | -1.59514 | 2.65008 | -0.03963 |
| C | -2.35902 | 1.47391 | 0.07098 |
| C | -1.70572 | 0.22451 | 0.04473 |
| C | -3.86056 | 1.57431 | 0.21416 |
| C | -4.59859 | 0.30366 | -0.23655 |
| C | -4.01427 | -0.8485 | 0.57477 |
| C | -2.49191 | -0.98314 | 0.41853 |
| O | -0.11338 | -1.90574 | -1.34623 |
| O | 2.54333 | 2.41978 | -0.41437 |
| O | -1.98603 | -2.0636 | 0.70627 |
| O | -4.6316 | -2.08682 | 0.25251 |
| C | -6.11504 | 0.43453 | -0.0617 |
| O | 4.73606 | 1.04877 | 0.33245 |
| C | 6.12833 | 0.95714 | 0.66488 |
| H | 4.28815 | -3.48152 | 0.13657 |
| H | 5.64624 | -1.47156 | 0.48349 |
| H | 1.85404 | -3.28461 | -0.4048 |
| H | 0.37639 | 3.505 | -0.17285 |
| H | -2.09998 | 3.61233 | -0.02479 |
| H | -4.11229 | 1.78363 | 1.26594 |
| H | -4.21195 | 2.44049 | -0.35799 |
| H | -4.37731 | 0.1251 | -1.29916 |
| H | -4.17853 | -0.62556 | 1.64687 |
| H | -4.00844 | -2.78483 | 0.53042 |
| H | -6.6303 | -0.46459 | -0.41283 |
| H | -6.49401 | 1.28956 | -0.63373 |
| H | -6.3779 | 0.58881 | 0.99294 |
| H | 6.27226 | 0.42657 | 1.61215 |
| H | 6.69107 | 0.46318 | -0.13434 |
| H | 6.46499 | 1.98896 | 0.76664 |

Standard orientation of rubiginone M (**7**) 2*R*, 3*S*_con3:


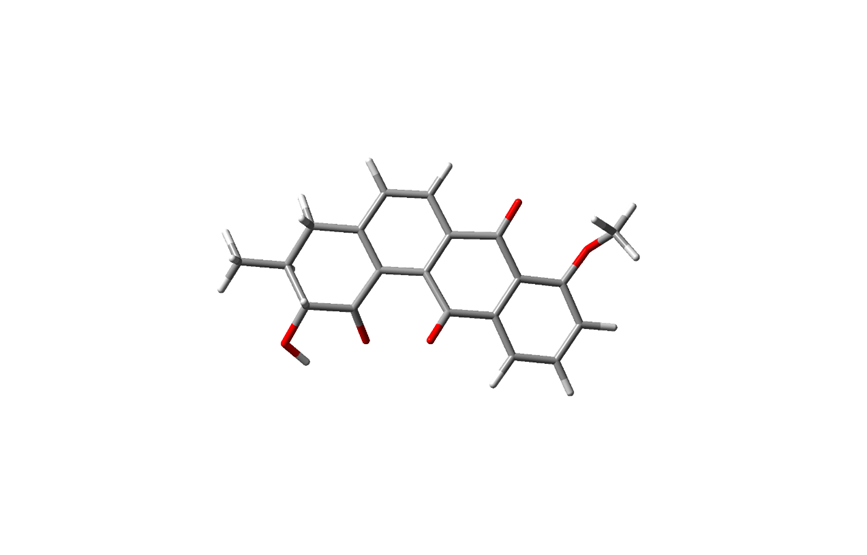


| C | 3.81953 | -2.60508 | -0.20275 |
| --- | --- | --- | --- |
| C | 4.58209 | -1.4726 | -0.48305 |
| C | 4.0225 | -0.19093 | -0.39484 |
| C | 2.66354 | -0.03507 | -0.04067 |
| C | 1.89945 | -1.19984 | 0.2144 |
| C | 2.47696 | -2.46988 | 0.1552 |
| C | 2.01059 | 1.2963 | 0.10028 |
| C | 0.51192 | 1.33129 | 0.10499 |
| C | -0.25503 | 0.15999 | 0.18534 |
| C | 0.4576 | -1.09339 | 0.58028 |
| C | -0.13142 | 2.57715 | 0.0097 |
| C | -1.51499 | 2.65113 | -0.00029 |
| C | -2.30527 | 1.48745 | 0.03145 |
| C | -1.66598 | 0.23275 | 0.07842 |
| C | -3.81332 | 1.61243 | 0.05409 |
| C | -4.58188 | 0.37113 | -0.42722 |
| C | -3.97755 | -0.88435 | 0.2412 |
| C | -2.50931 | -0.97324 | -0.15193 |
| O | -0.09489 | -1.96691 | 1.24205 |
| O | 2.64609 | 2.34494 | 0.18863 |
| O | -2.10916 | -1.9678 | -0.74932 |
| O | -4.65161 | -2.06847 | -0.14102 |
| C | -6.08249 | 0.50059 | -0.14662 |
| O | 4.80372 | 0.87781 | -0.74658 |
| C | 5.64476 | 1.4054 | 0.29613 |
| H | 4.26825 | -3.59155 | -0.27426 |
| H | 5.62233 | -1.5617 | -0.78217 |
| H | 1.86675 | -3.34036 | 0.37086 |
| H | 0.47313 | 3.47619 | -0.04553 |
| H | -2.00279 | 3.62077 | -0.05263 |
| H | -4.11885 | 2.48737 | -0.5303 |
| H | -4.10643 | 1.82758 | 1.09421 |
| H | -4.43606 | 0.26353 | -1.51214 |
| H | -4.04221 | -0.76926 | 1.33577 |
| H | -3.97875 | -2.63526 | -0.57135 |
| H | -6.27408 | 0.58911 | 0.93066 |
| H | -6.48543 | 1.3948 | -0.63686 |
| H | -6.62814 | -0.3697 | -0.52225 |
| H | 5.03352 | 1.80159 | 1.11269 |
| H | 6.22026 | 2.2115 | -0.16174 |
| H | 6.32505 | 0.63149 | 0.67058 |

Standard orientation of rubiginone M (**7**) 2*R*, 3*S* _con4:


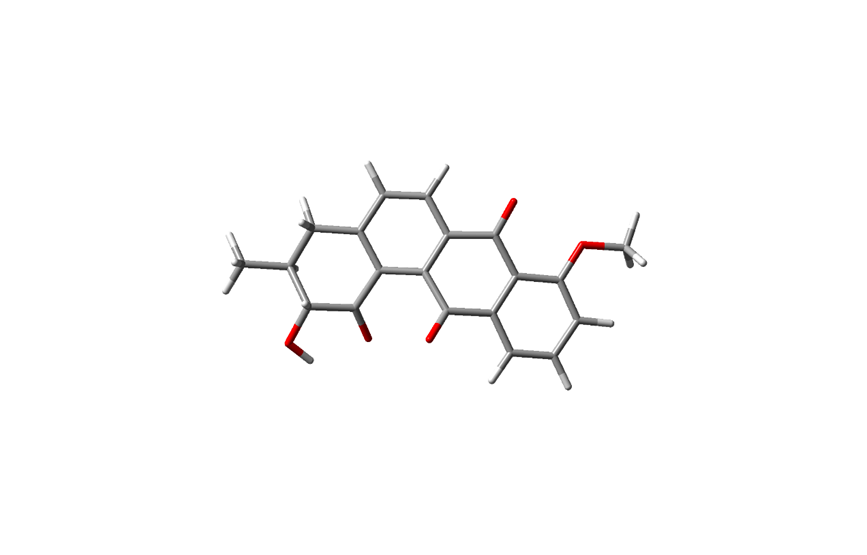


| C | 3.83357 | -2.50746 | -0.06655 |
| --- | --- | --- | --- |
| C | 4.60459 | -1.36541 | -0.26657 |
| C | 4.02921 | -0.08441 | -0.17111 |
| C | 2.64003 | 0.04104 | 0.11218 |
| C | 1.88443 | -1.14207 | 0.30022 |
| C | 2.47396 | -2.40455 | 0.22786 |
| C | 1.9592 | 1.35295 | 0.24848 |
| C | 0.45663 | 1.36096 | 0.22419 |
| C | -0.29461 | 0.17833 | 0.24143 |
| C | 0.42756 | -1.07241 | 0.61625 |
| C | -0.20525 | 2.59914 | 0.15644 |
| C | -1.58917 | 2.65273 | 0.11139 |
| C | -2.36179 | 1.47722 | 0.07552 |
| C | -1.70342 | 0.23239 | 0.09539 |
| C | -3.87184 | 1.5778 | 0.05406 |
| C | -4.60315 | 0.3433 | -0.49792 |
| C | -3.99907 | -0.92639 | 0.14321 |
| C | -2.5176 | -0.97706 | -0.20587 |
| O | -0.12993 | -1.97825 | 1.22957 |
| O | 2.56099 | 2.42258 | 0.34505 |
| O | -2.08238 | -1.94287 | -0.82597 |
| O | -4.64028 | -2.10616 | -0.30392 |
| C | -6.11413 | 0.43783 | -0.26314 |
| O | 4.74562 | 1.04415 | -0.35352 |
| C | 6.13772 | 0.95103 | -0.68619 |
| H | 4.30185 | -3.48456 | -0.14344 |
| H | 5.65783 | -1.47352 | -0.49316 |
| H | 1.86611 | -3.28788 | 0.38847 |
| H | 0.38603 | 3.5085 | 0.15034 |
| H | -2.09059 | 3.6165 | 0.08231 |
| H | -4.17333 | 2.46895 | -0.50752 |
| H | -4.20252 | 1.74903 | 1.09096 |
| H | -4.42003 | 0.27877 | -1.58067 |
| H | -4.10051 | -0.85336 | 1.23867 |
| H | -3.94342 | -2.64678 | -0.72985 |
| H | -6.34315 | 0.47907 | 0.80968 |
| H | -6.51579 | 1.34492 | -0.73029 |
| H | -6.63232 | -0.42482 | -0.69166 |
| H | 6.28123 | 0.41691 | -1.63153 |
| H | 6.70102 | 0.46026 | 0.1146 |
| H | 6.47421 | 1.98248 | -0.79214 |

**Fig. S46.** ^1^H NMR spectrum of compound **8** in CD_3_OD (500 MHz).

**Fig. S47.** ^13^C NMR spectrum of compound **8** in CD_3_OD (126 MHz).

**Fig. S48.** DEPT 135 spectrum of compound **8** (CD_3_OD).

**Fig. S49.** ^1^H–^1^H COSY spectrum of compound **8** (CD_3_OD).

**Fig. S50.** ^1^H–^13^C HSQC spectrum of compound **8** (CD_3_OD).

**Fig. S51.** ^1^H–^13^C HMBC spectrum of compound **8** (CD_3_OD).

**Fig. S52.** ^1^H–^1^H NOESY spectrum of compound **8** (CD_3_OD).

**Fig. S53.** PCR confirmation of the gene-deletion mutants in CB02414. (**a**) PCR confirmation of Z0004 and Z0005. (**b**) PCR confirmation of Z0008.


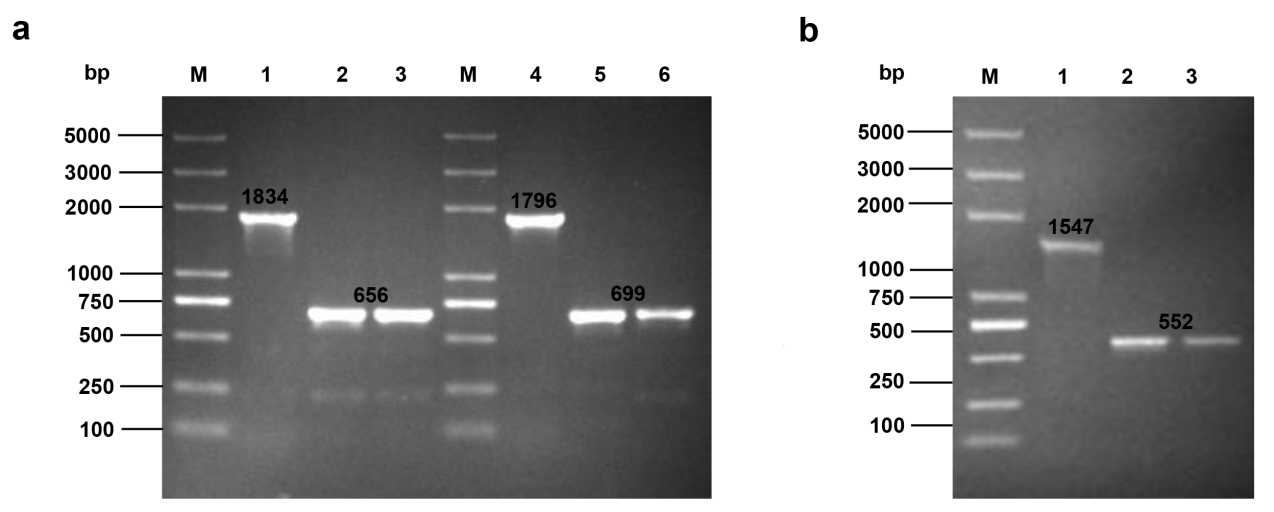


(**a**) M: DNA marker; amplification product of *rubN1* from CB02414 wild-type (**1**); the amplicon using plasmid Y0003 as a template (**2**); amplification product using the genomic DNA of Z0004 as a template (**3**); amplification product of *rubN*2 from CB02414 wild-type (**4**); the amplicon using plasmid Y0004 as a template (**5**); amplification product using the genomic DNA of Z0005 as a template (**6**).

(**b**) M: DNA marker; amplification product of *rubM4* from CB02414 wild-type (**1**); the amplicon using plasmid Y0007 as a template (**2**); amplification product using the genomic DNA of Z0008 as a template (**3**).

**Reference**

1. Bruhn T, Schaumlöffel A, Hemberger Y, Bringmann G. SpecDis: quantifying the comparison of calculated and experimental electronic circular dichroism spectra. Chirality. 2013;25(4):243–9.
